# Supplementary material for: Leading consumption patterns of psychoactive substances in Colombia: A deep neural network-based clustering-oriented embedding approach
Source: PLoS One. 2023 Aug 18;18(8):e0290098. doi: 10.1371/journal.pone.0290098 (PMC10438020; doi:10.1371/journal.pone.0290098)
Supplement: S1 Table — (DOCX) [file pone.0290098.s001.docx]

**SUPPLEMENTARY MATERIAL**

**Table S1.** Feature coherence measurements (goodness of fit).

|  | Cluster | KNN | AHC | RQ | RKNN |
| --- | --- | --- | --- | --- | --- |
| Geographical coherence (cluster) | 0 | 0.035 | 0.033 | 0.058 | 0.078 |
|  | 1 | 0.030 | 0.040 | 0.085 | 0.178 |
|  | 2 | 0.178 | 0.178 | 0.145 | 0.173 |
| CH Score |  | 18.424 | 18.445 | 8.536 | 10.240 |

CH: Calinski and Harabasz score; KNN: *k*-means clustering; AHC: Hierarchical Clustering; RQ: Regionalization with QEEN constraint; RKNN: Regionalization with KNN constraint.
