# Supplementary material for: Leading consumption patterns of psychoactive substances in Colombia: A deep neural network-based clustering-oriented embedding approach
Source: PLoS One. 2023 Aug 18;18(8):e0290098. doi: 10.1371/journal.pone.0290098 (PMC10438020; doi:10.1371/journal.pone.0290098)
Supplement: S2 Table — (DOCX) [file pone.0290098.s002.docx]

**SUPPLEMENTARY MATERIAL**

**Table S2.** Performance metrics for different models.

| Performance metric | CAE-DEC | PCA-*K*-means | CAE-Spectral |
| --- | --- | --- | --- |
| Calinski-Harabasz | 775992.45 | 128651.83 | 22468.26 |
| Davies-Bouldin | 0.2898 | 0.567 | 0.63 |
| Silhouette | 0.786 | 0.6061 | 0.62 |
