# Supplementary material for: Leading consumption patterns of psychoactive substances in Colombia: A deep neural network-based clustering-oriented embedding approach
Source: PLoS One. 2023 Aug 18;18(8):e0290098. doi: 10.1371/journal.pone.0290098 (PMC10438020; doi:10.1371/journal.pone.0290098)
Supplement: S3 Table — (DOCX) [file pone.0290098.s003.docx]

**SUPPLEMENTARY MATERIAL**

**Table S3.** Characteristics of the level of development, urbanity, rurality, and drug production in the regions of Colombia.

|  | Department | Urban | Rural | % Urban | % Rural | GDP | Drug Production |
| --- | --- | --- | --- | --- | --- | --- | --- |
| Caribbean (coastal areas) | Atlántico | 2404831 | 130686 | 94.80% | 5.20% | 52311 | - |
|  | Bolívar | 1549063 | 521047 | 74.80% | 25.20% | 41698 | - |
|  | Cesar | 903411 | 297163 | 75.20% | 24.80% | 23037 | 38 |
|  | Córdoba | 937319 | 847464 | 52.50% | 47.50% | 20570 | 2882 |
|  | La Guajira | 410636 | 469924 | 46.60% | 53.40% | 14466 | - |
|  | Magdalena | 938320 | 403426 | 69.90% | 30.10% | 15996 | 7 |
|  | San Andrés | 44893 | 16387 | 73.30% | 26.70% | 1761 | - |
|  | Sucre | 569089 | 335774 | 62.90% | 37.10% | 9659 | - |
|  |  |  |  | **68.8%*** | **31.2%*** | **179498**** | **2927**** |
| Eje Cafetero –Antioquia (Central region) | Antioquia | 4972941 | 1434161 | 77.60% | 22.40% | 176451 | - |
|  | Caldas | 740865 | 257390 | 74.20% | 25.80% | 19745 | - |
|  | Quindío | 471910 | 67994 | 87.40% | 12.60% | 9733 | - |
|  | Risaralda | 736164 | 207237 | 78.00% | 22.00% | 19263 | - |
|  |  |  |  | **79.3%*** | **20.7%*** | **225192**** | **-** |
| Pacific (Western region) | Cauca | 545902 | 918586 | 37.30% | 62.70% | 21107 | 17356 |
|  | Chocó | 243194 | 291632 | 45.50% | 54.50% | 4856 | 1248 |
|  | Nariño | 716592 | 914000 | 43.90% | 56.10% | 17971 | 36964 |
|  | Valle del Cauca | 3809542 | 666344 | 85.10% | 14.90% | 114864 | 2329 |
|  |  |  |  | **53.0%*** | **47.0%*** | **158798**** | **57897**** |
| Central-Eastern region | Boyacá | 708006 | 509370 | 58.20% | 41.80% | 30803 | - |
|  | Cundinamarca | 2090845 | 828215 | 71.60% | 28.40% | 72481 | - |
|  | Huila | 669697 | 430689 | 60,9% | 39,1% | 19473 | - |
|  | Norte de Santander | 1173712 | 317977 | 78.70% | 21.30% | 18360 | 41711 |
|  | Santander | 1655627 | 529210 | 75.80% | 24.20% | 74025 | 2 |
|  | Tolima | 907506 | 422681 | 68.20% | 31.80% | 25221 | - |
|  |  |  |  | **70.5%*** | **29.5%*** | **240363**** | **41713**** |
| Llanos orientales | Meta | 795061 | 244661 | 76.50% | 23.50% | 39459 | 1466 |
|  | Arauca | 172634 | 89540 | 65.80% | 34.20% | 6310 | - |
|  | Casanare | 295434 | 125070 | 70.30% | 29.70% | 17236 | - |
|  | Vichada | 25833 | 81975 | 24.00% | 76.00% | 775 | 245 |
|  |  |  |  | **59.2%*** | **40.8%*** | **63780**** | **1711**** |
| Southern region | Amazonas | 37047 | 39542 | 48.40% | 51.60% | 880 | 125 |
|  | Caquetá | 258280 | 143569 | 64,3% | 35,7% | 4670 | 4511,46 |
|  | Guainía | 20279 | 27835 | 42.10% | 57.90% | 431 | 22 |
|  | Guaviare | 45991 | 36776 | 55.60% | 44.40% | 931 | 3119 |
|  | Putumayo | 174539 | 173643 | 50.10% | 49.90% | 4091 | 24973 |
|  | Vaupés | 12090 | 28707 | 29.60% | 70.40% | 323 | 26 |
|  |  |  |  | **45.2%*** | **54.8%*** | **11326**** | **28265**** |

***Note:*** * average, ** sum. GPD is the Gross Domestic Product. The urban and rural areas represent the total population in each department. The production drug is measured in hectares. All records are for 2019.
