# Supplementary material for: Leading consumption patterns of psychoactive substances in Colombia: A deep neural network-based clustering-oriented embedding approach
Source: PLoS One. 2023 Aug 18;18(8):e0290098. doi: 10.1371/journal.pone.0290098 (PMC10438020; doi:10.1371/journal.pone.0290098)

**SUPPLEMENTARY MATERIAL**

**Figure S1**. Definition of the optimal number of clusters based on a Dendrogram.


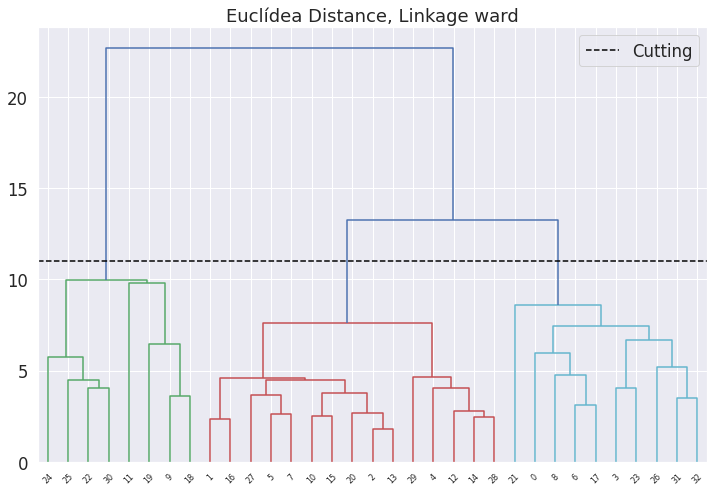

Supplement: S2 Fig — (DOCX) [file pone.0290098.s005.docx]
