# Supplementary material for: Leading consumption patterns of psychoactive substances in Colombia: A deep neural network-based clustering-oriented embedding approach
Source: PLoS One. 2023 Aug 18;18(8):e0290098. doi: 10.1371/journal.pone.0290098 (PMC10438020; doi:10.1371/journal.pone.0290098)

**SUPPLEMENTARY MATERIAL**

**Figure 2S.** Maps of Local Indicators of Spatial Association (LISA). Republished from [65] under a CC BY license, with permission from [ArcGIS Hub], original copyright [2016].


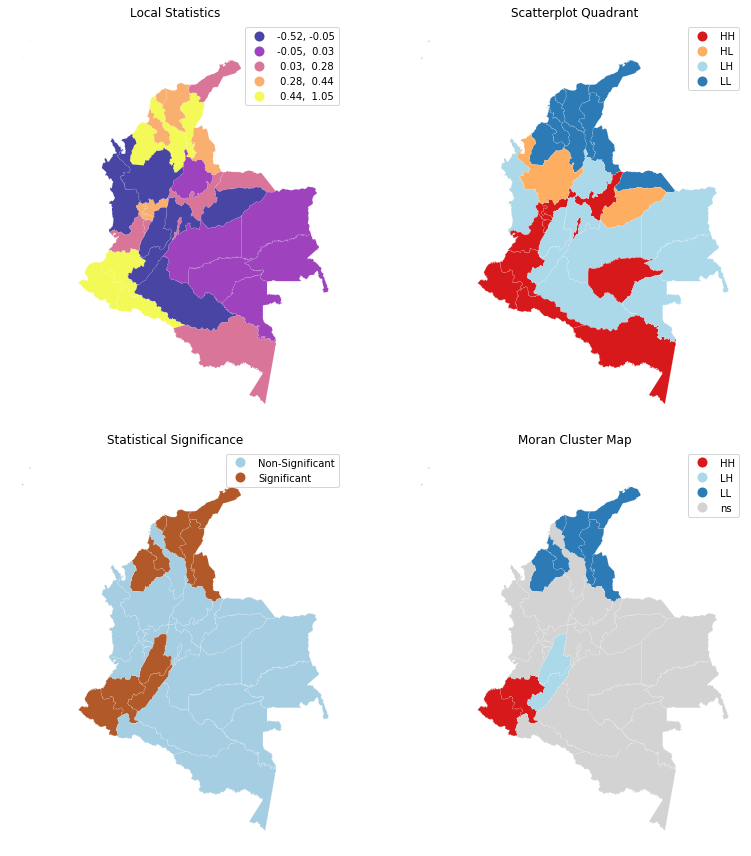

Supplement: S3 Fig — Republished from [65] under a CC BY license, with permission from [ArcGIS Hub], original copyright [2016]. (DOCX) [file pone.0290098.s006.docx]
