# Supplementary material for: Leading consumption patterns of psychoactive substances in Colombia: A deep neural network-based clustering-oriented embedding approach
Source: PLoS One. 2023 Aug 18;18(8):e0290098. doi: 10.1371/journal.pone.0290098 (PMC10438020; doi:10.1371/journal.pone.0290098)
Supplement: Attachment — Submitted filename: PONE-D-22-28262_reviewer.pdf [file pone.0290098.s007.pdf]

PLOS ONE

Leading Consumption Patterns of Psychoactive Substances in Colombia: A Deep Neural Network-based Clustering-oriented Embedding Approach

--Manuscript Draft--

**Manuscript Number:**	PONE-D-22-28262
**Article Type:**	Research Article
**Full Title:**	Leading Consumption Patterns of Psychoactive Substances in Colombia: A Deep Neural Network-based Clustering-oriented Embedding Approach
**Short Title:**	Leading Consumption Patterns of Psychoactive Substances in Colombia
**Corresponding Author:**	kevin palomino<br>Universidad del Norte<br>Barranquilla, COLOMBIA
**Keywords:**	Autoencoder; Machine learning; Spatial Analysis; clustering; Drug consumption/abuse; Psychoactive substances
**Abstract:**	The number of health-related incidents caused using illegal and legal psychoactive substances has dramatically increased over two decades worldwide. In Colombia, the use of illicit substances has increased up to 10.3%, while the consumption alcohol and tobacco has increased to 84% and 12%, respectively. To contribute to the understanding of the mental health problem caused by psychoactive substances and facilitate decision-making and national policy development processes, here we propose a deep clustering model integrating an autoencoder and spatial techniques to identify general and spatial patterns of consumption and/or abuse of drugs in Colombia. This model is capable of extracting features from the input data and identifying clusters during the learning process. As a test case, we used the largest publicly available database comprising 49,600 households. Using this approach, we estimated and geographically represented the prevalence of consumption and/or abuse of both substances and non-psychoactive substances in Colombia, while achieving statistically significant goodness-of-fit values. Our results indicate that variables such as region, sex, housing type, socioeconomic status, age, and variables related to household finances, statistically significantly contribute to explain the consumption and/or abuse patterns of psychoactive substances. We also identified three differential consumption and/or abuse patterns of psychoactive substances in the Colombian territory. These patters, at the spatial level, highlight concentrations of drug consumption in particular regions of the country, and associated with specific geographic locations as well as the inherent social and environmental contexts.
**Order of Authors:**	kevin palomino
	Carmen R. Berdugo
	Jorge I. Vélez
**Additional Information:**	
**Question**	**Response**
**Financial Disclosure**<br><br>Enter a financial disclosure statement that describes the sources of funding for the work included in this submission. Review the submission guidelines for detailed requirements. View published research articles from *PLOS ONE* for specific examples.<br><br>This statement is required for submission	The author(s) received no specific funding for this work.

Powered by Editorial Manager® and ProduXion Manager® from Aries Systems Corporation

and **will appear in the published article** if the submission is accepted. Please make sure it is accurate.<br><br>**Unfunded studies**<br>Enter: *The author(s) received no specific funding for this work.*<br><br>**Funded studies**<br>Enter a statement with the following details:<br>• Initials of the authors who received each award<br>• Grant numbers awarded to each author<br>• The full name of each funder<br>• URL of each funder website<br>• Did the sponsors or funders play any role in the study design, data collection and analysis, decision to publish, or preparation of the manuscript?<br>• **NO** - Include this sentence at the end of your statement: *The funders had no role in study design, data collection and analysis, decision to publish, or preparation of the manuscript.*<br>• **YES** - Specify the role(s) played.<br><br>* typeset	
**Competing Interests**<br><br>Use the instructions below to enter a competing interest statement for this submission. On behalf of all authors, disclose any competing interests that could be perceived to bias this work—acknowledging all financial support and any other relevant financial or non-financial competing interests.<br><br>This statement is required for submission and **will appear in the published article** if the submission is accepted. Please make sure it is accurate and that any funding sources listed in your Funding Information later in the submission form are also declared in your Financial Disclosure statement.<br><br>View published research articles from *PLOS ONE* for specific examples.	The authors have declared that no competing interests exist.

**NO authors have competing interests**<br><br>Enter: *The authors have declared that no competing interests exist.*<br><br>**Authors with competing interests**<br><br>Enter competing interest details beginning with this statement:<br><br>*I have read the journal's policy and the authors of this manuscript have the following competing interests: [insert competing interests here]*<br><br>* typeset	
**Ethics Statement**<br><br>Enter an ethics statement for this submission. This statement is required if the study involved:<br><br>• Human participants<br>• Human specimens or tissue<br>• Vertebrate animals or cephalopods<br>• Vertebrate embryos or tissues<br>• Field research<br><br>Write "N/A" if the submission does not require an ethics statement.<br><br>General guidance is provided below. Consult the submission guidelines for detailed instructions. **Make sure that all information entered here is included in the Methods section of the manuscript.**	This research does not require an ethics statement approved by an ethics committee since we are using public information where there is no identification or individual information of the people involved.

Powered by Editorial Manager® and ProduXion Manager® from Aries Systems Corporation

**Format for specific study types**<br><br>**Human Subject Research (involving human participants and/or tissue)**<br>• Give the name of the institutional review board or ethics committee that approved the study<br>• Include the approval number and/or a statement indicating approval of this research<br>• Indicate the form of consent obtained (written/oral) or the reason that consent was not obtained (e.g. the data were analyzed anonymously)<br><br>**Animal Research (involving vertebrate animals, embryos or tissues)**<br>• Provide the name of the Institutional Animal Care and Use Committee (IACUC) or other relevant ethics board that reviewed the study protocol, and indicate whether they approved this research or granted a formal waiver of ethical approval<br>• Include an approval number if one was obtained<br>• If the study involved *non-human primates*, add *additional details* about animal welfare and steps taken to ameliorate suffering<br>• If anesthesia, euthanasia, or any kind of animal sacrifice is part of the study, include briefly which substances and/or methods were applied<br><br>**Field Research**<br><br>Include the following details if this study involves the collection of plant, animal, or other materials from a natural setting:<br>• Field permit number<br>• Name of the institution or relevant body that granted permission	
**Data Availability**<br><br>Authors are required to make all data underlying the findings described fully available, without restriction, and from the time of publication. PLOS allows rare exceptions to address legal and ethical concerns. See the PLOS Data Policy and FAQ for detailed information.	Yes - all data are fully available without restriction

A Data Availability Statement describing where the data can be found is required at submission. Your answers to this question constitute the Data Availability Statement and **will be published in the article**, if accepted.<br><br>**Important:** Stating 'data available on request from the author' is not sufficient. If your data are only available upon request, select 'No' for the first question and explain your exceptional situation in the text box.<br><br>Do the authors confirm that all data underlying the findings described in their manuscript are fully available without restriction?	
**Describe where the data may be found in full sentences. If you are copying our sample text, replace any instances of XXX with the appropriate details.**<br><br>• If the data are **held or will be held in a public repository**, include URLs, accession numbers or DOIs. If this information will only be available after acceptance, indicate this by ticking the box below. For example: *All XXX files are available from the XXX database (accession number(s) XXX, XXX.).*<br>• If the data are all contained **within the manuscript and/or Supporting Information files**, enter the following: *All relevant data are within the manuscript and its Supporting Information files.*<br>• If neither of these applies but you are able to provide **details of access elsewhere**, with or without limitations, please do so. For example:<br><br>*Data cannot be shared publicly because of [XXX]. Data are available from the XXX Institutional Data Access / Ethics Committee (contact via XXX) for researchers who meet the criteria for access to confidential data.*<br><br>*The data underlying the results presented in the study are available from (include the name of the third party*	N/A

and contact information or URL).<br>• This text is appropriate if the data are owned by a third party and authors do not have permission to share the data.<br><br>* typeset	
Additional data availability information:	

Manuscript Click here to access/download;Manuscript;Manuscript.docx

Leading Consumption Patterns of Psychoactive Substances in Colombia: A Deep Neural Network-based Clustering-oriented Embedding Approach

Kevin Palomino[1,#], Carmen R. Berdugo[1], Jorge I. Vélez[1]

September 1, 2022

[1] *Universidad del Norte, Barranquilla, Colombia.*

[#] Correspondence to be directed to:

Mr. Kevin Palomino,
Department of Industrial Engineering,
Universidad del Norte,
Km 5 via Puerto Colombia,
Barranquilla, Colombia
E-mail: krpalomino@uninorte.edu.co

ORCIDs

KP: https://orcid.org/0000-0002-2708-1096
JIV: https://orcid.org/0000-0002-3146-7899
CB: https://orcid.org/0000-0002-4803-2816

Running Title: Leading Consumption Patterns of Psychoactive Substances in Colombia
Number of Characters: 136 (title), 67 (running title)
Number of Words: 236 (abstract), 5888 (main text)
Number of Keywords: 6
Number of Figures: 9
Number of Tables: 2

ABSTRACT

The number of health-related incidents caused using illegal and legal psychoactive substances has dramatically increased over two decades worldwide. In Colombia, the use of illicit substances has increased up to 10.3%, while the consumption alcohol and tobacco has increased to 84% and 12%, respectively. To contribute to the understanding of the mental health problem caused by psychoactive substances and facilitate decision-making and national policy development processes, here we propose a deep clustering model integrating an autoencoder and spatial techniques to identify general and spatial patterns of consumption and/or abuse of drugs in Colombia. This model is capable of extracting features from the input data and identifying clusters during the learning process. As a test case, we used the largest publicly available database comprising 49,600 households. Using this approach, we estimated and geographically represented the prevalence of consumption and/or abuse of both substances and non-psychoactive substances in Colombia, while achieving statistically significant goodness-of-fit values. Our results indicate that variables such as region, sex, housing type, socioeconomic status, age, and variables related to household finances, statistically significantly contribute to explain the consumption and/or abuse patterns of psychoactive substances. We also identified three differential consumption and/or abuse patterns of psychoactive substances in the Colombian territory. These patters, at the spatial level, highlight concentrations of drug consumption in particular regions of the country, and associated with specific geographic locations as well as the inherent social and environmental contexts.

Keywords**:** Autoencoder, Machine Learning, Spatial analysis, Clustering, Drug consumption/abuse, Psychoactive substances.

1. INTRODUCTION

Psychoactive substances (PAS) are chemical substances that change the function of the nervous system and cause alterations in the perception, mood, consciousness, cognition, or behavior of people (OPS, 2022). PAS can be grouped according to their chemical structure as synthetic cannabinoids, synthetic cathinones, phenethylamines, arylcyclohexylamines, tryptamines, indo-lalkylamines, new synthetic opioids, piperazines, ketamine, and designer benzodiazepines; and according to their origin as of natural origin or synthetic molecules (Assi et al., 2017; Heesun et al., 2016; Riley et al., 2020).

The increase numbers of drug use among young people is drawing the attention of national governments (Lukić et al., 2021). Because the number of health-related incidents caused by using illegal and legal psychoactive substances worldwide has dramatically increased over the last two decades (Heesun et al., 2016; Uchiyama et al., 2013), this phenomena has become some of the largest burdens of disease (CDC, 2020; Grant et al., 2004). Drug use constitute a major cost to society due to premature mortality, increased health expenditure, criminal justice (drug and micro-trafficking), social welfare costs, and other social consequences (Goetzel et al., 2003; Stewart et al., 2003).

Colombia is ranked as one of the largest drug producers in the world (Garcia & Murillo, 2021). Unfortunately, production and commercialization of drugs through drug trafficking and micro-trafficking, that constantly expands in locations with high levels of poverty and a limited presence of the government (Aschner & Montero, 2020). Statistics and indicators of drug consumption, production, and distribution, as well as reports from the National Statistical System (DANE) of Colombia, highlight a dramatic increase in (1) drug production (ODC, 2022); (2) intern consumption of psychoactive substances at early ages; and (3) the prevalence in the use and/or abuse of drugs have dramatically increased over the last 20 years (DANE, 2014, 2020). Furthermore, the country also has the highest prevalence of drug use among school students in recent years when compared to other Latin American countries (UNODC, 2021a). Thus, there is an urgent need to develop effective interventions to prevent the use and/or abuse of psychoactive substances.

The first step towards reducing this consumption is to identify drug consumption patterns in the general population (Griffiths & Mcketin, 2003). Numerous studies have identified patterns associated with drug use and consumption (Lanier et al., 2012; Martins et al., 2015). According to the Center for Disease Control and Prevention, people who do not have their own homes and live in rented accommodation are more likely to use drugs (CDC, 2015). Other research studies suggest that neighborhood contextual characteristics may increase the risk of substance abuse (Bozorgi et al., 2021; Fite et al., 2009; Friedman et al., 2016; Fuller et al., 2005; Jensen et al., 2017; Sarah & Leonard A, 2018) and that population density may also influence substance use and overdose risk through a higher level of socialization in densely populated urban areas (Galea et al., 2005; Latkin et al., 2003; Schroeder et al., 2001). Other authors have identified that anxiety, sleep disorders, suicide, depression, and other mental illnesses constitute risk factors for the consumption and abuse of psychoactive substances (Campo-Arias et al., 2020; Fajardo, 2018). In addition, the early use of marijuana increases the risk of consuming other psychoactive substances (Scoppetta & Castaño, 2019), and people involved in sports and artistic activities consider drugs as an enhancer element for developing such activities (Scheuer et al., 2018).

In Colombia, drug consumption patterns and risk factors have also been identified. Indeed, Kalyanam *et* al. (2007)(Kalyanam et al., 2017) analyze the social impact of bazuco and inhalant use among street youths; Narvaez-Chicaiza (2020) (Narvaez-Chicaiza, 2020) assesses social factors leading to the adoption of harm reduction policies and how such factors influence treatments for substance abuse disorders, and Restrepo-Escobar & Cardona (2021)(Restrepo-Escobar & Cardona, 2021) show that university students with low satisfaction in their studies are heavy users of alcohol, tobacco, and marijuana. However, these approaches do not consider the use of Machine Learning (ML) and/or Deep Data Mining techniques in combination with spatial models to analyze drug consumption data from the general population. In this sense, to the best of our knowledge, we found no robust models integrating ML and spatial models to identify drug consumption patterns using Colombian publicly available databases.

Although several techniques for analyzing drug consumption patterns are currently available (i.e., ML, Bayesian, spatial, traditional multivariate, or univariate statistical models, or, in some cases, a combination of these), new trends in pattern identification and analysis techniques focus on hybrid and ensemble models (Hancer et al., 2020). Currently, the most widely used ML techniques are Support Vector Machines (SVMs), Random Forest (RF) and Natural Language Processing (NLP)(Conway & O'Connor, 2016; Henriksson et al., 2015; Katsuki et al., 2015; Squeglia et al., 2017; Wager et al., 2013). Among Bayesian models, Bayesian meta-regression (DisMod-MR), Bayesian hierarchical models, and Markov Chain Monte Carlo are the most attractive methods (Bowman et al., 2008; Degenhardt et al., 2014; Whiteford et al., 2013). Regarding spatial models, Spatial Distributions, Spatial Regression Models, Spatial Scan Statistics, Variograms, and Social Mapping are the most used techniques (Bass & Lambert, 2004; Chaix et al., 2006; Freisthler et al., 2005; Shannon et al., 2008). In terms of traditional statistical models, logistic regression, confirmatory factor analysis, and correlational analysis are the most employed techniques to disclose drug-associated patterns (Chichester et al., 2020; Geissert et al., 2018; Mowbray et al., 2003; Peet & Stokes, 2005).

Fraley and Raftery in (1998) suggest separating clustering approaches into two groups: hierarchical and partitioning techniques. Partitioning techniques are divided into density-, model-, and grid-based methods, the most popular of which are *K*-means, PAM, CLARA, CLARA, NSM, FCM, EM, DBSCAN, CLIQUE. Hierarchical techniques, on the other hand, are divided into agglomerative and divisive methods; of these, the best-known methods are BRICH, CURE, ROCK, CHAMELEON (see Saxena et al. en el (2017) for further reading). Although all these techniques have been shown to have a good fit if not relevant features are removed *a priori*, it is well known that, in clustering algorithms, irrelevant and redundant features in the data may degrade the quality of clusters, which leads to high computational cost. Therefore, removing such features may alleviate these issues. In this paper, we focus on identifying patterns of psychoactive substance consumption using an ensemble model that integrates an autoencoder with a clustering algorithm and a spatial model. As part of our approach, we used the most recent and representative works for data

clustering, as well as different dimensionality reduction and feature selection methodologies proposed in the literature.

Feature selection approaches for clustering can be split into filter, wrapper, embedded, and hybrid approaches (Hancer et al., 2020). While wrappers depend on the clustering algorithms of choice to evaluate the clustering quality of a selected feature subset, filters are independent of the clustering algorithm. Embedded approaches also work with a clustering algorithm and, unlike wrappers, incorporate knowledge about the clustering structure. Another type of methods are hybrid approaches, which combine filter and wrapper approaches into a single strategy. However, studies on embedded and hybrid feature selection approaches in clustering are limited (Hancer et al., 2020).

Other feature learning-based approaches using Deep Neural Networks have been shown to work well for linear and nonlinear models (B. Li et al., 2021). For instance, Xie et al. in (2016), propose to simultaneously work on feature extraction and clustering using pre-trained Auto-Encoders. Although deep clustering methods such as IDEC (Guo et al., 2017), DEPICT (Dizaji et al., 2017), DBC (F. Li et al., 2017) , DualAAE (Ge et al., 2020), VAED (Lim et al., 2020), and DNC (B. Li et al., 2021) have been developed, these are mainly used to work and process images. In general, deep clustering models use Auto-Encoders since they can learn input features without having labels on the data; performance measures show that this approach is reliable for different types of data (Sharifipour et al., 2020). Thus, deep clustering methods have become a growing field of research for feature selection (Sharifipour et al., 2020). In this regard, the use of convolutional networks in autoencoders and data from different statistical distributions, as well as how to apply feature selection for clustering, remain open questions and have not yet been fully addressed (Hancer et al., 2020).

Here, we propose a Deep Neural Network-based Clustering-oriented Embedding Algorithm that allows us to (*i*) identify consumption patterns of psychoactive substances; and (*ii*) build an ensemble algorithm integrating an autoencoder with a clustering algorithm and a spatial model to jointly deal with the feature space and cluster memberships. Our approach is based on the model proposed by Xie et al. (2016) and B. Li et al. in (2021) and expands their work by creating an autoencoder

from a convolutional network instead of a linear one to both accurately represent high-order interactions in the data and simultaneously incorporate a spatial analysis to properly describe drug consumption patterns. Our main hypothesis is that incorporating these two key elements in our proposal will help to identify and better understand drug consumption patterns and support national policy development processes.

2. MATERIAL AND METHODS

2.1 Study Area

Located in South America, the republic of Colombia is a diverse country with a population of over 48 million people distributed in a territory of 440,831 square miles (DANE, 2018) encompassing jungles, highlands, grasslands, deserts, coasts, and islands, distributed in six regions and 32 departments (states)(DNP, 2018).

According to the United Nations Office on Drugs and Crime, Colombia is the first cocaine producing country, and the eighth country with the highest production of cannabis (UNODC, 2021b). Regarding consumption, the Colombian Drug Observatory indicates that the use of illicit substances in the territory has increased to 10.3%, with men between 18 and 24 years old being the heaviest consumers of these types of drugs. Reports also indicate that consumption of licit substances such as alcohol and tobacco have recently dramatically increased (ODC, 2019).

2.2 Data Sources

We used two databases for the analysis of drug consumption patterns in Colombia. The first data base was obtained from the National Survey of Psychoactive Substance Consumption in the General Population during 2019 conducted by the National Statistical System (DANE) of Colombia (DANE, 2020). This data includes observations of 49,600 households, where information on housing, location, general characteristics of individuals, consumption of legal and illegal psychoactive substances and implemented treatments is registered. The second data base comes from the Colombian Drug Observatory and contains information on the production of psychoactive substances during 2019. These databases were georeferenced using polygons (i.e., a shapefile) as implemented in ArcGIS Hub (Espinosa, 2022). This research does not require an ethics statement approved by an ethics committee since we are using public information where there is no identification or individual information of the people involved.

2.3 Convolutional Auto-Encoder-Deep Embedded Clustering Algorithm

Figure 1 presents the proposed Convolutional Auto-Encoder- Deep Embedded Clustering (CAE-DEC) framework, which is based on the implementation presented by Xie et al. (2016). However, unlike the Xie et al. (2016) model, our scheme is constructed by applying convolutional layers for the deep autoencoder (DA) architecture instead of a linear one to represent high-order interactions in the data. In addition, a spectral clustering-based centroid estimation is proposed to achieve an improved initial centroids calculation.

In our approach, an encoder structure is first applied to map the input vector into a lower feature space, called latent feature space (LFS). Then, the LFS is independently passed through a decoder structure and through a clustering layer to achieve an efficient clustering framework. The encoder-decoder combination, known as DA, attempts to extract a LFS preserving the relevant information from the original input data. On the other hand, the clustering layer seeks to execute an improved clustering assignment by minimizing the divergence between a target distribution and a centroid-based probability distribution.

As a complementary part of this research, a spatial analysis was performed using the feature space generated from the autoencoder as input. Here, the spatial data exploration is initially explored using global spatial autocorrelation in order to determine to which establish to which degree the similarity between observations in a dataset relates to the similarity of the locations of such observations (Anselin, 1988). To assess global spatial autocorrelation, the Moran's I (Moran, 1948), Geary's *C* (Geary, 1954), and Getis and Ord's *G* (Getis & Ord, 1992) statistics are estimated. We also measure the Local Spatial Autocorrelation, which focuses on the relationships between each observation and its surroundings, rather than providing a single-number summary of these relationships across the map (Anselin, 1995). This is estimated based on the ability to determine whether spatial autocorrelation is present in a geographically referenced data set. Finally, we perform regionalization, which corresponds to a special kind of clustering where the objective is to group similar observations based on their statistical attributes and their spatial location (Duque et

al., 2016). In this sense, regionalization embeds the same logic as standard clustering techniques, while applying a series of geographical constraints (Rey et al., 2020).

2.1. Convolutional Auto-Encoder (CAE)

The DA is a deep neural network architecture capable of learning unsupervised representations of an input data set. Typically, DA networks are used for dimensionality reduction or denoising tasks. The structure of a DA is based on two deep networks: a network to transform the original input data into a latent feature space, and a network trained to reconstruct the original input data using the extracted latent space as input. The first network, used to extract the latent space, is called encoder, while the second network is called decoder. Rather than using fully connected layers, the implemented DA architecture incorporates convolutional (CONV) layers and fully connected (FC) layers for LFS extraction and reconstruction (Figure 1). The integration of convolutional layers in a DA is also called convolutional auto-encoder (CAE) (Masci et al., 2011). Compared to a DA built with only fully connected layers, the CAE structure can reduce the number of parameters compared to a DA (Shrestha & Mahmood, 2019).

2.1.1 Convolutional Layer

The proposed CAE structure is designed using four convolutional layers, two CONV layers during the encoder stage, and two CONV layers during the decoder stage, and two fully connected layers (Figure 1). The convolution operation can be denoted as:

$$z_{i,j,k}^{l} = W_k^{l^T} X_{i,j}^{l} + b_k^{l}$$

where $z_{i,j,k}^{l}$ is the value of each feature at the (i, j) location in the k-th feature map of the l-th layer, W_k^l and b_k^l represent the weight and bias of the k-th filter of the l-th layer, and $X_{i,j}^l$ denotes the input value at location (i, j) of the l-th layer. For non-linear mapping, an activation function $g(.)$ is applied over the convolutional feature $z_{i,j,k}^{l}$ as follows:

$$a_{i,j,k}^{l} = g(z_{i,j,k}^{l})$$

where $a_{i,j,k}^{l}$ is the activation value resulting from applying the activation function $g(.)$. The Rectified Linear Unit (ReLU) function is set as activation function on each CONV, except in the final decoder CONV layer where a sigmoidal activation will be applied.

2.2 Clustering Layer

The clustering layer is inspired by Xie et al. (2016). Initially, a soft assignment is computed between the latent space, also known as embedded space, and the cluster centroids. Then, update steps are repeated to define the final cluster centroids and embedded space. The Kullback–Leibler (KL) divergence is used as loss function during the optimization procedure. The objective is to minimize de KL divergence between a soft clustering distribution Q and an auxiliar target distribution P. The KL loss is calculated as:

$$L_c = KL(P\|Q) = \sum_k \sum_m p_{km} log \frac{p_{km}}{q_{km}}$$

where L_c is the clustering loss. To measure the similarity between embedded point z_k and the cluster centroid cm, the t Student's distribution is used as a kernel:

$$q_{km} = \frac{(1 + \|z_k - c_m\|^2/\alpha)^{-\frac{\alpha+1}{2}}}{\sum_i (1 + \|z_k - c_i\|^2/\alpha)^{-\frac{\alpha+1}{2}}}$$

with α the degrees of freedom of the t Student's distribution and q_{km} is a a soft clustering assignment distribution of each embedded point (i.e, probability of assigning point k to the cluster m). As in Xie et al. (2016), when setting α=1 the similarity function q_{km} can be calculated as:

$$q_{km} = \frac{(1 + \|z_k - c_m\|^2)^{-1}}{\sum_i (1 + \|z_k - c_i\|^2)^{-1}}$$

To compute the target distribution p_{km}, the q_{km} second power is defined, and a cluster normalization is applied as follows:

$$p_{km} = \frac{q_{km}^2 / \sum_k q_{km}}{\sum_i (q_{ki}^2 / \sum_k q_{ki})}$$

Then, by minimizing the divergence between P and Q, the embedding learning is achieved through highly confident assignments.

2.2.1 Center Initialization

As previously mentioned, the cluster centroids are initialized using a spectral clustering-based approach. The spectral clustering allows flexible distance metrics and provides better cluster estimations than k-means (Xie et al. 2016). However, most spectral clustering algorithms have high computational requirements. To overcome these computational requirements, random samples are be taken to estimate the cluster centroids. As spectral clustering does not estimate any centroid during the learning process, once the clusters are defined, the mean of each cluster is used as the centroid estimator.

2.3 The CAE-DEC Model

Initially, the input data are normalized within the interval [0, 1]. This normalization allows the network to use the most advanced learning rate and avoid the vanishing o gradient problems likewise to alleviate over-fitting. Further, to achieve a better learning process, the last CONV layer in the decoder structure is activated by a sigmoid activation function. Then, two training steps will be executed during the CAE-DEC learning process. Firstly, a CAE model will be trained to minimize the reconstruction loss L_r as follows:

$$L_r = ||x - \tilde{x}||^2$$

where x is the normalized input and $\tilde{x}$ is the reconstructed output. This pretrained CAE model is then used as the DA structure in the CAE-DEC model.

In the second step, the CAE-DEC model is trained to simultaneously minimize reconstruction loss and clustering loss. The total loss during this training step will be set as

$$L_t = L_r + C \times L_c$$

where L_r is the CAE-DEC reconstruction loss, L_c is the CAE-DEC clustering loss, and C is a coefficient to control the loss balance. The training process is shown in Table 1. The goal is to obtain a latent space that minimizes the total loss, and the label of each embedded point must be established. The final label will be obtained as

$$Label_j = \arg max_m q_{jm}$$

where the q_{jm} value can be seen as the probability that point *j* belongs to a specific cluster center *m*. On the other hand, the maximum number of iterations Mint and the target distribution P update condition P_change were chosen based on multiple experiments. The final Mint and P_change values were 3000 and 5, respectively. This final P_change improves stability during the training process.

3. RESULTS

3. 1 Model Comparison for Identifying Drug Consumption Patterns

Figure 2 depicts the LFS resulting after applying both the CAE and CAE-DEC models to the data. Among all individuals, we identified three different clusters; 14935 (30.19%) individuals belong to cluster 0, 11528 (23.30%) individuals belong to cluster 1, and 23005 (46.50%) individuals belong to cluster 2. Interestingly, the LFS generated with the CAE-DEC has more defined clusters compared to that generated by the CAE model. Although the CAE model seeks to extract a LFS that preserves the important characteristics of the input data, our proposed CAE-DEC model not only preserves these important characteristics but, at the same time, also forces the encoder structure to generate representative clusters while extracting the new feature space.

On the other hand, the reconstruction loss obtained through the CAE model is higher than that of the CAE-DEC model. This result may be related to the fact that the CAE-DEC model used the pre-trained CAE model during its construction. It should be noted that the CAE model alone is not able to determine the labels of each point or define clusters in the data. Thus, clusters showed in Figure 2 were obtained through spectral clustering and were the bases for initializing the centroids in the CAE-DEC model.

3.2 Identification of Clusters of Psychoactive Drugs Consumption

Here we analyse the patterns within each of the clusters obtained using the CAE-DEC model. In particular, we defined a priority dummy variable $\underline{Y}_{ij}$ quantifying whether the *i*th person in household *j*th has consumed psychoactive substances; $Y_{ij} = 1$ when an individual has never consumed psychoactive substances and $Y_{ij} = 2$ when the individual has consumed one or more substances (i.e., tranquilizers, stimulants, methadone or opioid analgesics without a medical prescription, or inhalants, DICK, popper, marijuana, cocaine, bazuco, ecstasy, heroin, methamphetamine, Lysergic acid diethylamide [LSD], mushrooms, yage, cacao sabanero, ketamine, Gamma-hydroxybutyrate [GHB], 4-Bromo-2,5-dimethoxyphenethylamine [2CB] or any other drugs).

Out of the 49468 individuals in the sample, only 5514 (11.15%) consume psychoactive substances. Figure 3a and 3b depict, respectively, the derived cluster structure derived with the CAE-DEC model for individuals consuming psychoactive substances and those who reported not to consume. Our results indicate that individuals belonging to clusters 0 and 2 are more likely to consume some type of psychoactive substances (Figure 3a), while most individuals in cluster 1 do not consume psychoactive substances (Table 1). In particular, 1726 (11.56%) individuals in cluster 0, 392 (3.4%) individuals in cluster 1 and 3396 (14.76%) individuals in cluster 2 have used psychoactive substances (Table 1). A χ^2-based test of independence reveals that the region where individuals are located, age (years), the type of household they live in, their socioeconomical status (SES) and whether they contribute to the household finances, are statistically significantly associated to the cluster they belong to (Table 2).

Table 3 shows the adjusted residuals for our model. According to our results, the greatest contribution of the Region variable is made by the Central-Eastern region. In this region, the observed value is higher than the expected value in cluster 2, while the observed value is lower than the expected value for cluster 0. Although to a lesser extent, the Llanos Orientales region also shows a large contribution to the χ^2 statistic. Indeed, this region shows a lower number of observed individuals compared to the expected number of individuals in cluster 2 and a higher number observed than expected individuals in clusters 0 and 1 (Table 3).

On the other hand, Gender has a higher-than-expected value of males in clusters 0 and 2, while it is lower in cluster 1. For females, the opposite occurs in cluster 1, and lower values are observed in clusters 0 and 2. Similarly, Housing Type has a higher-than-expected value of individuals living at houses in cluster 1, and a lower-than-expected in cluster 2. Conversely, cluster 2 has a higher number of individuals living in apartments, and cluster 1 has the lowest (Table 3).

Regarding the SES, we found a higher-than-expected number of individuals in strata 3, 4, 5, and 6 in cluster 0 (Table 3). We also observed a lower-than-expected number of individuals in strata 3, 4, 5, and 6 in cluster 2, and a higher-than-expected number of individuals in strata 1, 4 and 6 in

cluster 1 (Table 3). Moreover, the age variable shows a higher-than-expected observed value for the (0,20] range in cluster 0. For ages between (20,40] years, cluster 2 has a higher-than-expected number of individuals. Conversely, there is a lower number of individuals in cluster 1. Finally, the results for the household economy variable show that cluster 2 has a higher-than-expected value of individuals contributing to the household finances, and cluster 1 has a lower-than-expected value of individuals not contributing to it. Comparison of Calinski, Davies and silhouette metrics between a principal component analysis (PCA)-based deep autoencoder (PCA-DEC) and our proposed CAE-DEC model indicates the superiority of the latter (**Table 2S, Supplementary Material**).

3.3 Spatial Analysis of Psychoactive Drugs Consumption

To determine the number of choropleth class limits, different alternative classification algorithms were used (i.e., Equal Intervals, Quantiles, Maximum Breaks, Box plot, Head-Tail Breaks, Jenks-Caspall, Fisher-Jenks and Max-p) and compared using the absolute deviation around class medians optimization criterion (Figure 4). According to our results, the Fisher-Jenks classifier performed better and hence was selected.

Following the same exploratory spatial analysis, we constructed a choropleth with the percentage of psychoactive substance use for each of the 32 Colombian departments (Figure 5a). Our results indicate that the departments of Arauca, Vichada, Caquetá, Chocó, Magdalena, Cesar, Bolivar, Sucre, Cordoba, and Norte de Santander have low percentages of drug use. However, some of these departments are major drug producers (i.e., Cordoba and Guaviare) according to data from the Drug Observatory of Colombia (ODC, 2021). Similarly, Putumayo is the department with the highest proportions of psychoactive substance use (Figure 5a).

Results of the global Moran's I show the presence of a statistically significant positive global spatial autocorrelation ($I = 0.2005$, $P<0.01$). Thus, the null hypothesis that the map is random (i.e., that the map shows more spatial patterns than we would expect if the values had been randomly assigned to a location) is rejected. In addition, other global indices such as Geary's *C* (*C*

= 0.693, *P* = 0.003), and Getis and Ord's *G* (*G* = 0.800, *P* = 0.049) confirm the presence of statistically significant global spatial autocorrelation.

To further explore the relationships between each observation and its environment, the Local Indicators of Spatial Association (LISA) were estimated (more information on LISA statistics is provided in **Figure 2S** of the **Supplementary Material**). Figure 5b depicts the Moran diagram, indicating the positive (or negative) association within each quadrant. Specifically, the high-high (HH) and low-low (LL) quadrants indicate a positive association with high and low drug use, respectively. On the other hand, [illegible]he low-high (LH) and high-low (HL) quadrants indicate negative associations of drug use (Figure 5b). Following our results, we found that departments such as Nariño and Cauca belong to the HH (hot spot) cluster, while La Guajira, Atlántico, Magdalena, Cesar, Norte de Santander, Sucre, and Cordoba belong to the LL (col[illegible]ot). This clustering pattern leads to a statistically significant Moran's I statistic (*P*-value <0.01). Thus, a little over 39.4% of the departments are considered, by this analysis, to be part of a spatial cluster (i.e., statistically significant with *P*-value <5%).

Figure 6 shows the illegal and legal drugs more frequently consumed in Colombia. Among legal drugs, alcohol and tobacco are the most frequently consumed in the national territory (Figure 6a), while marijuana, followed by non-prescription tranquilizers and Yagé, and a slight consumption of opioids and Poppers, are the most frequently consumed illegal drugs (Figure 6b).

In terms of legal drugs, alcohol has the highest consumption rates in Bogotá, Cundinamarca, and Chocó (Figure 7). However, there is moderately high use in Vaupés, Nariño, Bolívar, Magdalena, La Guajira, and Atlántico (Figure 8). As for energy drinks, consumption is the highest in Casanare and Guaviare, and has slightly high uses in Boyacá, Nariño, Risaralda, and Arauca. On the other hand, tobacco has the highest consumption in Cundinamarca, but has moderately high uses in Bogotá, Boyacá, Nariño, Casanare, Tolima, Quindío, Risaralda, Guainía, Caldas, and Vaupés. It should also be mentioned that the use of these drugs is also present across the country, but with a lower incidence (Figure 7).

Regarding illegal drugs, non-prescription tranquilizers and stimulants are most prevalent in Casanare (Figure 8). However, the consumption of tranquilizers is slightly higher in Nariño, while inhalants have the highest consumption in Quindio, followed by Cauca, Caldas, and Nariño. DICK has the highest use in Cauca and a high consumption in Quindio and Nariño; while Antioquia, followed by Caldas and Risaralda, show the highest consumption of popper. On the contrary, marijuana has its highest consumption in Risaralda and moderately high consumption in Caldas, Bogotá, Antioquia, and Quindío. As for cocaine, its consumption is the highest in Risaralda and moderately high in Antioquia (Figure 8).

On the other hand, bazuco has the highest consumption rate in Guaviare, and critical consumption in Nariño, Cauca, Quindio, Antioquia, and Amazonas; ecstasy has its highest consumption in Risaralda, followed by Bogotá and Caldas; heroin consumption is highest in Vaupes, Huila, Cauca, Quindio, and Arauca, and is slightly higher in Casanare; methamphetamine consumption is highest in Casanare and is moderately high in Boyacá; methadone is most widely used in Quindio, but has slightly high levels of use in Valle del Cauca and Caquetá; opioids are most prevalent in Casanare, followed by Sucre; LSD is most prevalent in Bogotá, but has high levels of use in Caldas, Risaralda, Quindio, and Nariño; mushrooms have their highest consumption in Boyacá and have moderately high uses in Quindio, Risaralda, Bogotá, Cauca, and Casanare; Yagé has a higher incidence in Putumayo; cacao sabanero has its highest consumption in Caldas, and has moderate consumption in Cundinamarca, Bogotá, Antioquia, and Quindio; ketamine has the highest consumption in Casanare, followed by Antioquia; and GHB has the highest consumption in Risaralda, followed by Santander, Valle del Cauca, and Norte de Santander. Finally, 2CB has the highest consumption rate in Risaralda, followed by Caldas. Although the consumption pattern of some departments is not mentioned, there is low and moderate consumption for certain drugs in some of them (Figure 8).

3.4 Regionalization of Clusters

We applied a regionalization method as a grouping technique for imposing a spatial restriction, i.e., the result of a regionalization algorithm contains clusters with geographically coherent areas, in addition to having coherent data profiles. Our approach uses a spatially constrained hierarchical clustering algorithm, which identified three clusters representing the consumption of psychoactive substances in the country (Figure 9). The number of clusters was estimated based on the average silhouette indexes, the total intra-cluster variance, and dendrograms (**Figure 1S, Supplementary Material**). Following our results, cluster 0 is comprised by departments such as La Guajira, Cesar, Atlántico, Magdalena, Norte de Santander, Bolivar, Sucre, and Cordoba, all of them located at the Northern region of the country; cluster 1 is comprised by Antioquia, Santander, Boyacá, Caldas, Risaralda, and Quindío; and cluster 2 is integrated by the remaining departments (Figure 9). When testing geographical coherence, which is the measure that assesses the “compactness” of a given shape, our results indicate that the clusters derived using the regionalization model represent moderately compact regions. In addition, the feature coherence (i.e., goodness-of-fit) test using different metrics showed that our 3-cluster regionalization structure properly fits the data (**Table 1S, Supplementary Material**).

4. DISCUSSION

In this study, we a Deep Neural Network-based Clustering-oriented Embedding algorithm (i.e., a ML-based model) for identifying patterns of psychoactive substance use in the Colombian context. This model allows to automatically extract features from the input data (i.e., sex, age, socioeconomic status, housing type, among others) to determine whether an individual has consumed psychoactive substances, and creates clusters in the new data space generated during the learning process as proposed by B. Li et al., 2021 and Xie et al., 2016 . After the training process, a latent feature space (LFS) is generated, and the analysis of the results is subsequently performed. Among the main results, we highlight the existence of clearly marked clusters where the prevalence of individuals who use or do not use psychoactive substances is identified. In addition, we identified that region, sex, housing type, socioeconomic status, age, and whether individuals contribute to the household finances are statistically impacted to the clustering structure. This result is in line with previous studies aimed at identifying consumption patterns of psychoactive substances (Clarke et al., 2014; Kuo et al., 2016; Martins et al., 2015). Interestingly, when comparing the CAE-DEC model proposed herein and the CAE-Spectral model using different score metrics (i.e., Silhouette score, which measures the internal density of each cluster and the distance that separates them from each other, the Calinski-Harabasz index and the Davies-Bouldin index [DBI]) showed that the our proposal model performs better (Silhouette: 0.62 vs. 0.786; Calinski-Harabasz: 22468.26 vs. 775992.45; DBI: 0.2898 vs. 0.63) than the CAE-Spectral alone model.

Following the results of our model, we found that individuals more likely to have consumed psychoactive substances are grouped in cluster 2 (Table 1). On the other hand, cluster 1 is comprised of individuals that do not consume psychoactive substances. This cluster is characterized by a greater proportion of females, belong to socioeconomical strata 1, are 40 years of age or older, and do not contribute economically to the support their household. In contrast, cluster 2 is characterized by a higher proportion of male, a higher proportion of individuals in strata 1 and 2, with ages between 20 and 40 years, that do not contribute to the household economy (Table 1).

Finally, cluster 0 is characterized by a small proportion of males, a higher proportion of individuals in strata 3, 4, 5, and 6, and individuals that are more likely to contribute to the household economy (Table 1).

At the level of spatial statistics, we identified that legal drugs such as alcohol have a high prevalence in all regions of Colombia, with a slight tendency to more consumption in coastal areas (Figure 7). Likewise, tobacco is present in all regions with a higher proportion in the Central region where climate conditions resemble temperate weather. Similarly, energy drinks are more frequently used in the Eastern region, which is characterized by a continental climate surrounded by flat territory. These results are in line with the scientific literature suggesting that the location of regions within countries is directly associated with the consumption of psychoactive substances (Bozorgi et al., 2021; García et al., 2019; Keyes et al., 2014; King et al., 2014).

Our findings also show that the Southern region is more likely to consume illegal drugs such as bazuco, heroin and Yagé (Figure 8). One of the main reasons for this result is that, unfortunately, this region has favourable environmental characteristics for their consumption and production. In the Western region, consumption mostly include of DICK, GHB, heroin, opioids, and methamphetamines. As for the Central region, there is a more diverse consumption pattern, where the use of drugs such as marijuana, popper, cocaine, ecstasy, inhalants, methadone, heroin, LSD, GHB, 2CB, and mushrooms prevails. The Eastern region is characterized by consumption of drugs such as heroin, bazuco, non-prescription tranquilizers, stimulants, methamphetamines, opioids, and ketamine. Interestingly, consumption of illegal drugs is lower in the Northern region compared to other regions of the country. However, there is a more representative consumption of drugs such as non-prescription tranquilizers, opioids, ketamine, GHB, and heroine, with the Atlántico department having the highest proportion of consumption within this region (Figure 8).

In summary, the proposed model simultaneously integrates a feature extraction process within the clustering design, prioritizing features that improve the separation between groups, thus avoiding the manual extraction of features, which is a frequent process in traditional models.

Additionally, a geospatial component is sequentially included to expand the resulting insights by considering geographic constraints. Currently, these types of architectures are scarce in the understanding of mental health problems. As part of future work, the architecture of the proposed model could be improved to integrate the automatic extraction of features while optimizing a geospatial loss. Following our experience with the proposed CAE-DEC in psychoactive substance consumption, the application of this model to other mental health problems such as suicide, depression, and domestic violence, among other pathologies, should be timely explored. Based on these results, effective interventions and/or government policies to prevent and/or mitigate their impact could be promoted and evaluated.

Acknowledgements. K.P. is a doctoral student at Universidad del Norte, Barranquilla, Colombia, and received a Ph.D. scholarship from this institution. Some of this work is to be presented to the Ph.D. program in partial fulfillment of the requirements for the Ph.D. degree. The sponsor of the study has no role in study design; in the collection, analysis, and interpretation of data; in the writing of the report; and in the decision to submit the article for publication.

Authors' Contributors. All authors contributed to the design of the study. K.P. was responsible for data extraction and analysis; JIV and CB provided important insight and supervised the analysis. All authors reviewed the manuscript.

Declaration of Competing Interest. None to declare.

FIGURE LEGENDS

Figure 1. Architecture of the proposed CAE-DEC model.

Figure 2: Derived Latent Feature Space based on the **(a)** CAE and **(b)** CAE-DEC models.

Figure 3. Resulting clusters for individuals **(a)** consuming and **(b)** not consuming psychoactive substances based on the CAE-DEC model.

Figure 4. Absolute deviation around class medians (ADCM) statistic criterion for different alternative classifiers. Here, lower is better.

Figure 5. (a) Consumption percentage of psychoactive substances according to the CAE-DEC model; **(b)** Moran's I statistic; **(c)** Moran's cluster map. Here, HH, LH, LL and ns represent high-high, low-high, low-low and not statistically significant quadrants, respectively. This clustering pattern leads to a statistically significant Moran's I statistic of 0.2 (*P*-value <0.01).

Figure 6. Frequency of consumption of **(a)** legal and **(b)** illegal drugs in Colombia.

Figure 7. Consumption of illegal drugs by department. For interpretation purposes, number represents values scaled on a range of 0 to 1. For instance, Bogotá D.C. has the highest LSD consumption and Putumayo has the lowest.

Figure 8. Consumption of legal drugs by department. Number represents values scaled on a range of 0 to 1 for psychoactive substance use. Conventions as in Figure 7.

Figure 9. Cluster map of drug use after regionalization.

TABLES

Table 1. Pseudo code for the CAE-DEC training process.

```
Pseudo code: The CAE-DEC training process
--------------------------------------------------------------------------------
Input data: Number of clusters n; Normalized input data x; Maximum number of iterations Mint;
Balance coefficient C; Pretrained CAE; Stop condition Stop; Target distribution P update
condition P_change.
Training process:
1. Generate an initial latent space (Z) through the pre-trained CAE
2. Run spectral clustering with Z to generate the initial cluster centers (C)
3. Initialize the CAE-DEC model with the pretrained CAE.
4. Calculate soft assignment distribution Q and target distribution P based on Z and C
for epoch<Minter do:
        if epoch%P_change==0 then:
                Calculate soft assignment distribution Q and target distribution P based on Z and C
        end if
        Feed the CAE-DEC with the normalized input data x
        Calculate the reconstruction loss and the clustering loss
        Update CAE-DEC parameters. Weight, Bias, and Centers.
        if Stop==True then:
            Break
        end if
end for
Obtain the label for each data point from the las optimized Q.
Output: Latent space, labels
```

Table 2. Distribution of demographic and social variables across clusters.

Variables		Cluster 0 (*n*=14935)	Cluster 1 (*n*=11528)	Cluster 2 (*n*=23005)	χ^2	*df*	*P*-value
Region	Caribbean	3004	2991	4075	1472.9	10	<.0001
	Central-Eastern	2526	2299	6175			
	Central-Southern	1843	1299	1702			
	Eje Cafetero – Antioquia	3902	2354	6371			
	Llanos Orientales	1687	1305	1496			
	Pacific	1973	1280	3186			
Gender	Male	6606	3927	10233	386.72	2	<.0001
	Female	8329	7601	12772			
Housing type	House	8250	6538	11834	114.18	6	<.0001
	Apartment	6275	4724	10595			
	Room	395	256	543			
	Indigenous dwelling	15	10	33			
Socioeconomic status	1	3889	3626	6945	843.92	10	<.0001
	2	4627	3902	8851			
	3	4284	2867	5683			
	4	1341	718	979			
	5	510	256	362			
	6	284	159	185			
Age (years)	(0, 20]	2105	1576	2998	345.87	4	<.0001
	(20, 40]	6814	4329	10889			
	(40, 68]	6016	5623	9118			
Contribute to the household finances	Yes	10128	7473	16041	85.24	2	<.0001
	No	4807	4055	6964			

df: Degrees of freedom.

Table 3. Adjusted residuals comparing the observed and expected frequencies based on the cluster analysis.

	Variables	Cluster 0 (*n*=14935)	Cluster 1 (*n*=11528)	Cluster 2 (*n*=23005)
Region	Caribbean	-0.88	17.02	-13.61
	Central-Eastern	-18.72	-6.76	22.97
	Central-Southern	12.54	6.09	-16.7
	Eje Cafetero - Antioquia	2.02	-14.36	10.31
	Llanos Orientales	11.32	9.59	-18.55
	Pacific	0.84	-6.97	5.13
Gender	Male	6.68	-19.66	10.52
	Female	-6.68	19.66	-10.52
Housing type	House	4.17	7.13	-9.88
	Apartment	-4.83	-6.61	10.05
	Room	2.2	-1.54	-0.72
	Indigenous dwelling	-0.72	-1.09	1.59
Socioeconomic status	1	-10.26	5.99	4.37
	2	-12.72	-3.3	14.51
	3	9.14	-3	-5.87
	4	17.29	0.44	-16.29
	5	11.12	-0.49	-9.82
	6	8.26	1.2	-8.62
Age (years)	(0, 20]	2.54	0.61	-2.85
	(20, 40]	3.2	-17.23	11.66
	(40, 68]	-4.98	16.93	-9.77
Contribute to the household finances	Yes	-0.61	-8.37	7.65
	No	0.61	8.37	-7.65

References

Anselin, L. (1988). *Spatial Econometrics: Methods and Models* (Springer Netherlands). https://doi.org/10.1007/978-94-015-7799-1_1

Anselin, L. (1995). Local Indicators of Spatial Association—LISA. *Geographical Analysis*, *27*(2), 93–115. https://doi.org/10.1111/J.1538-4632.1995.TB00338.X

Aschner, J. P., & Montero, J. C. (2020). Architectures, spaces, and territories of illicit drug trafficking in Colombia and Mexico: *Https://Doi.Org/10.1177/1741659020910212*, *17*(3), 327–351. https://doi.org/10.1177/1741659020910212

Assi, S., Gulyamova, N., Kneller, P., & Osselton, D. (2017). The effects and toxicity of cathinones from the users' perspectives: A qualitative study. *Human Psychopharmacology: Clinical and Experimental*, *32*(3), e2610. https://doi.org/10.1002/HUP.2610

Bozorgi, P., Porter, D. E., Eberth, J. M., Eidson, J. P., & Karami, A. (2021). The leading neighborhood-level predictors of drug overdose: A mixed machine learning and spatial approach. *Drug and Alcohol Dependence*, *229*, 109143. https://doi.org/10.1016/J.DRUGALCDEP.2021.109143

Campo-Arias, A., Suárez-Colorado, Y. P., & Caballero- Domínguez, C. C. (2020). Asociación entre el consumo de Cannabis y el riesgo de suicidio en adolescentes escolarizados de Santa Marta, Colombia. *Biomédica*, *40*(3), 569. https://doi.org/10.7705/BIOMEDICA.4988

CDC. (2015). *Today's Heroin Epidemic* . Centers for Disease Control and Prevention. https://www.cdc.gov/vitalsigns/heroin/index.html

CDC. (2020). *Understanding the Epidemic*. https://www.cdc.gov/opioids/basics/epidemic.html

Clarke, H., Soneji, N., Ko, D. T., Yun, L., & Wijeysundera, D. N. (2014). Rates and risk factors for prolonged opioid use after major surgery: population based cohort study. *BMJ*, *348*. https://doi.org/10.1136/BMJ.G1251

DANE. (2014). *Estudio nacional de consumo de sustancias psicoactivas en Colombia*. https://www.unodc.org/documents/colombia/2014/Julio/Estudio_de_Consumo_UNODC.pdf

DANE. (2018). *Departamento Administrativo Nacional de Estadística. Censo Nacional de Población y Vivienda 2018. Proyecciones de Población 2018-2020, total municipal por área Junio 30*.

DANE. (2020). *Encuesta Nacional de Consumo de Sustancias Psicoactivas*. https://www.dane.gov.co/files/investigaciones/boletines/encspa/comunicado-encspa-2019.pdf

DNP. (2018). *Avances y complementariedades estratégicas de los Distritos en el marco de los esquemas asociativos territoriales*. https://colaboracion.dnp.gov.co/CDT/Desarrollo Territorial/Conversatorio Distrito Cali 04_10_2018 - Santiago Arroyo.pdf

Duque, J. C., Ramos, R., & Suriñach, J. (2016). Supervised Regionalization Methods: A Survey: *Http://Dx.Doi.Org/10.1177/0160017607301605*, *30*(3), 195–220. https://doi.org/10.1177/0160017607301605

Espinosa, J. (2022). *Shapefile*. https://hub.arcgis.com/datasets/de0e829ddbf743c895ba6dcee1b74fae/about

Fajardo, A. L. (2018). Consumption of psychopharmaceuticals in the city of Bogota (Colombia): a new reality. *ARCHIVOS DE MEDICINA*, *18*(2). https://doi.org/10.30554/archmed.18.2.2743.2018

Fite, P. J., Wynn, P., Lochman, J. E., & Wells, K. C. (2009). The Influence of Neighborhood Disadvantage and Perceived Disapproval on Early Substance Use Initiation. *Addictive Behaviors*, *34*(9), 769. https://doi.org/10.1016/J.ADDBEH.2009.05.002

Friedman, S. R., Tempalski, B., Brady, J. E., West, B. S., Pouget, E. R., Williams, L. D., Des Jarlais, D. C., & Cooper, H. L. F. (2016). Income inequality, drug-related arrests, and the health of people who inject drugs: Reflections on seventeen years of research. *The International Journal on Drug Policy*, *32*, 11–16. https://doi.org/10.1016/J.DRUGPO.2016.03.003

Fuller, C. M., Borrell, L. N., Latkin, C. A., Galea, S., Ompad, D. C., Strathdee, S. A., & Vlahov, D. (2005). Effects of race, neighborhood, and social network on age at initiation of injection drug

use. *American Journal of Public Health*, *95*(4), 689–695. https://doi.org/10.2105/AJPH.2003.02178

Galea, S., Rudenstine, S., & Vlahov, D. (2005). Drug use, misuse, and the urban environment. *Drug and Alcohol Review*, *24*(2), 127–136. https://doi.org/10.1080/09595230500102509

Garcia, F. L. G., & Murillo, J. C. A. (2021). The United Nations and 21st century security challenges in Colombia. *Revista Cientifica General Jose Maria Cordova*, *19*(36), 929–940. https://doi.org/10.21830/19006586.875

García, M. C., Heilig, C. M., Lee, S. H., Faul, M., Guy, G., Iademarco, M. F., Hempstead, K., Raymond, D., & Gray, J. (2019). Opioid Prescribing Rates in Nonmetropolitan and Metropolitan Counties Among Primary Care Providers Using an Electronic Health Record System — United States, 2014–2017. *MMWR. Morbidity and Mortality Weekly Report*, *68*(2), 25–30. https://doi.org/10.15585/MMWR.MM6802A1

Geary, R. C. (1954). The Contiguity Ratio and Statistical Mapping. *The Incorporated Statistician*, *5*(3), 115. https://doi.org/10.2307/2986645

Getis, A., & Ord, J. K. (1992). The Analysis of Spatial Association by Use of Distance Statistics. *Geographical Analysis*, *24*(3), 189–206. https://doi.org/10.1111/J.1538-4632.1992.TB00261.X

Goetzel, R. Z., Hawkins, K., Ozminkowski, R. J., & Wang, S. H. (2003). The health and productivity cost burden of the “top 10” physical and mental health conditions affecting six large US employers in 1999. *JOURNAL OF OCCUPATIONAL AND ENVIRONMENTAL MEDICINE*, *45*(1), 5–14. https://doi.org/10.1097/00043764-200301000-00007

Grant, B. F., Stinson, F. S., Dawson, D. A., Chou, S. P., Dufour, M. C., Compton, W., Pickering, R. P., & Kaplan, K. (2004). Prevalence and co-occurrence of substance use disorders and independent mood and anxiety disorders - Results from the national epidemiologic survey on alcohol and related conditions. *ARCHIVES OF GENERAL PSYCHIATRY*, *61*(8), 807–816. https://doi.org/10.1001/archpsyc.61.8.807

Griffiths, P., & Mcketin, R. (2003). Developing a global perspective on drug consumption patterns

and trends-the challenge for drug epidemiology. *Bulletin on Narcotics*, *5*(1).

Hancer, E., Xue, B., & Zhang, M. (2020). A survey on feature selection approaches for clustering. *Artificial Intelligence Review 2020 53:6*, *53*(6), 4519–4545. https://doi.org/10.1007/S10462-019-09800-W

Heesun, C., Jaesin, L., & Eunmi, K. (2016). Trends of novel psychoactive substances (NPSs) and their fatal cases. *Forensic Toxicology*, *34*(1), 1–11. https://jglobal.jst.go.jp/en/detail?JGLOBAL_ID=201602283742633549

Jensen, M., Chassin, L., & Gonzales, N. A. (2017). Neighborhood Moderation of Sensation Seeking Effects on Adolescent Substance Use Initiation. *Journal of Youth and Adolescence*, *46*(9), 1953. https://doi.org/10.1007/S10964-017-0647-Y

Kalyanam, J., Katsuki, T., R.G. Lanckriet, G., & Mackey, T. K. (2017). Exploring trends of nonmedical use of prescription drugs and polydrug abuse in the Twittersphere using unsupervised machine learning. *Addictive Behaviors*, *65*, 289–295. https://doi.org/10.1016/j.addbeh.2016.08.019

Keyes, K. M., Cerdá, M., Brady, J. E., Havens, J. R., & Galea, S. (2014). Understanding the Rural–Urban Differences in Nonmedical Prescription Opioid Use and Abuse in the United States. *American Journal of Public Health*, *104*(2), e52. https://doi.org/10.2105/AJPH.2013.301709

King, N. B., Fraser, V., Boikos, C., Richardson, R., & Harper, S. (2014). Determinants of Increased Opioid-Related Mortality in the United States and Canada, 1990–2013: A Systematic Review. *American Journal of Public Health*, *104*(8), e32. https://doi.org/10.2105/AJPH.2014.301966

Kuo, Y. F., Raji, M. A., Chen, N. W., Hasan, H., & Goodwin, J. S. (2016). Trends in Opioid Prescriptions Among Part D Medicare Recipients From 2007 to 2012. *The American Journal of Medicine*, *129*(2), 221.e21-221.e30. https://doi.org/10.1016/J.AMJMED.2015.10.002

Lanier, W. A., Johnson, E. M., Rolfs, R. T., Friedrichs, M. D., & Grey, T. C. (2012). Risk factors for prescription opioid-related death, Utah, 2008-2009. *Pain Medicine (Malden, Mass.)*, *13*(12), 1580–1589. https://doi.org/10.1111/J.1526-4637.2012.01518.X

Latkin, C. A., Forman, V., Knowlton, A., & Sherman, S. (2003). Norms, social networks, and HIV-related risk behaviors among urban disadvantaged drug users. *Social Science & Medicine (1982)*, *56*(3), 465–476. https://doi.org/10.1016/S0277-9536(02)00047-3

Li, B., Pi, D., Lin, Y., & Cui, L. (2021). DNC: A Deep Neural Network-based Clustering-oriented Network Embedding Algorithm. *Journal of Network and Computer Applications*, *173*. https://doi.org/10.1016/J.JNCA.2020.102854

Lukić, V., Micić, R., Arsić, B., Nedović, B., & Radosavljević, Ž. (2021). Overview of the major classes of new psychoactive substances, psychoactive effects, analytical determination and conformational analysis of selected illegal drugs. *Open Chemistry*, *19*(1), 60–106. https://doi.org/10.1515/CHEM-2021-0196/XML

Martins, S. S., Sampson, L., Cerdá, M., & Galea, S. (2015). Worldwide Prevalence and Trends in Unintentional Drug Overdose: A Systematic Review of the Literature. *American Journal of Public Health*, *105*(11), e29–e49. https://doi.org/10.2105/AJPH.2015.302843

Moran, P. (1948). *The Interpretation of Statistical Maps* (2nd ed., Vol. 10). Journal of the Royal Statistical Society. https://www.jstor.org/stable/2983777

Narvaez-Chicaiza, M. A. (2020). Harm Reduction Policies Where Drugs Constitute a Security Issue. *Health Care Analysis*, *28*(4), 382–390. https://doi.org/10.1007/S10728-020-00415-9

ODC. (2019). *Estudio nacional de consumo de sustancias psicoactivas*. https://www.odc.gov.co/Portals/1/publicaciones/pdf/estudio Nacional de consumo 2019.pdf

ODC. (2021). *Density of drug production in Colombia*. https://www.datos.gov.co/d/acs4-3wgp/visualization

ODC. (2022). *Observatorio de drogas de Colombia*. https://www.minjusticia.gov.co/programas-co/ODC/Paginas/SIDCO-departamento-municipio.aspx

OPS. (2022). *Abuso de sustancias* . Organización Panamericana de La Salud. https://www.paho.org/es/temas/abuso-sustancias

Restrepo-Escobar, S. M., & Cardona, E. A. S. (2021). Educational and prevention campaigns. A

review on the use of psychoactive substances in Colombian university students. *Interdisciplinaria*, *38*(2), 199–208. https://doi.org/10.16888/INTERD.2021.38.2.13

Rey, S., Arribas-Bel, D., & Wolf, L. (2020). *Geographic Data Science with Python*. https://geographicdata.science/book/intro.html

Riley, A. L., Nelson, K. H., To, P., López-Arnau, R., Xu, P., Wang, D., Wang, Y., Shen, H. wei, Kuhn, D. M., Angoa-Perez, M., Anneken, J. H., Muskiewicz, D., & Hall, F. S. (2020). Abuse potential and toxicity of the synthetic cathinones (i.e., “Bath salts”). *Neuroscience and Biobehavioral Reviews*, *110*, 150–173. https://doi.org/10.1016/J.NEUBIOREV.2018.07.015

Sarah, C., & Leonard A, J. (2018). Contextual Perspectives on Heroin Addiction and Recovery: Classic and Contemporary Theories. *International Archives of Public Health and Community Medicine*, *2*(1). https://doi.org/10.23937/IAPHCM-2017/1710009

Scheuer, C., Boot, E., Carse, N., Clardy, A., Gallagher, J., Heck, S., Marron, S., Martinez-Alvarez, L., Masarykova, D., Mcmillan, P., Murphy, F., Steel, E., Ekdom, H. Van, & Vecchione, H. (2018). El consumo de sustancias psicoactivas en jóvenes estudiantes de una institución educativa del municipio de Neira (Caldas): un estudio de caso desde la mirada de la educación inclusiva. *Cultura y Droga*, *23*(26), 343–354. https://doi.org/10.2/JQUERY.MIN.JS

Schroeder, J. R., Latkin, C. A., Hoover, D. R., Curry, A. D., Knowlton, A. R., & Celentano, D. D. (2001). Illicit drug use in one’s social network and in one’s neighborhood predicts individual heroin and cocaine use. *Annals of Epidemiology*, *11*(6), 389–394. https://doi.org/10.1016/S1047-2797(01)00225-3

Scoppetta, O., & Castaño, G. A. (2019). Early drug consumption and subsequent risk of illicit drug use in Colombia. *Addictive Disorders and Their Treatment*, *18*(1), 10–14. https://doi.org/10.1097/ADT.0000000000000144

Stewart, W. F., Ricci, J. A., Chee, E., Hahn, S. R., & Morganstein, D. (2003). Cost of lost productive work time among US workers with depression. *JAMA-JOURNAL OF THE AMERICAN MEDICAL ASSOCIATION*, *289*(23), 3135–3144.

https://doi.org/10.1001/jama.289.23.3135

Uchiyama, N., Matsuda, S., Kawamura, M., Kikura-Hanajiri, R., & Goda, Y. (2013). Two new-type cannabimimetic quinolinyl carboxylates, QUPIC and QUCHIC, two new cannabimimetic carboxamide derivatives, ADB-FUBINACA and ADBICA, and five synthetic cannabinoids detected with a thiophene derivative α-PVT and an opioid receptor agonist AH-7921 identified in illegal products. *Forensic Toxicology 2013 31:2*, *31*(2), 223–240. https://doi.org/10.1007/S11419-013-0182-9

UNODC. (2021a). *Drogas sintéticas y nuevas sustancias psicoactivas en América Latina y el Caribe 2021*. https://www.minjusticia.gov.co/programas-co/ODC/Documents/Publicaciones/GlobalSmartLA (1).pdf?csf=1&e=MH9EHg

UNODC. (2021b). *Monitoreo de territorios afectados por cultivos ilícitos 2020*. https://www.unodc.org/documents/crop-monitoring/Colombia/Colombia_Monitoreo_de_territorios_afectados_por_cultivos_ilicitos_2020.pdf

Xie, J., Girshick, R., & Farhadi, A. (2016). Unsupervised Deep Embedding for Clustering Analysis. *33rd International Conference on Machine Learning, ICML 2016*, *1*, 740–749. https://arxiv.org/abs/1511.06335v2

Figure 1

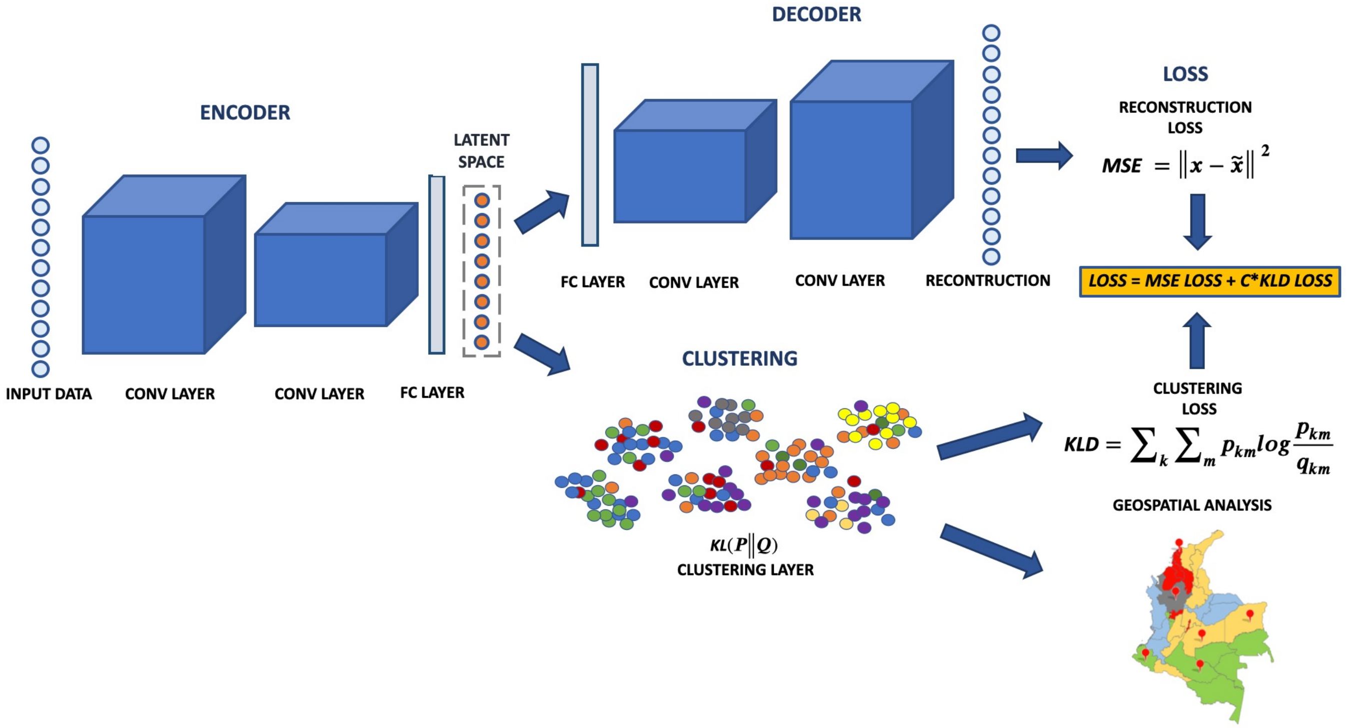
ENCODER
INPUT DATA
CONV LAYER
CONV LAYER
FC LAYER
LATENT SPACE
DECODER
FC LAYER
CONV LAYER
CONV LAYER
RECONTRUCTION
LOSS
RECONSTRUCTION LOSS
$MSE = \|x - \tilde{x}\|^2$
LOSS = MSE LOSS + C*KLD LOSS
CLUSTERING
$KL(P\|Q)$
CLUSTERING LAYER
CLUSTERING LOSS
$KLD = \sum_k \sum_m p_{km} log \frac{p_{km}}{q_{km}}$
GEOSPATIAL ANALYSIS

Figure 2

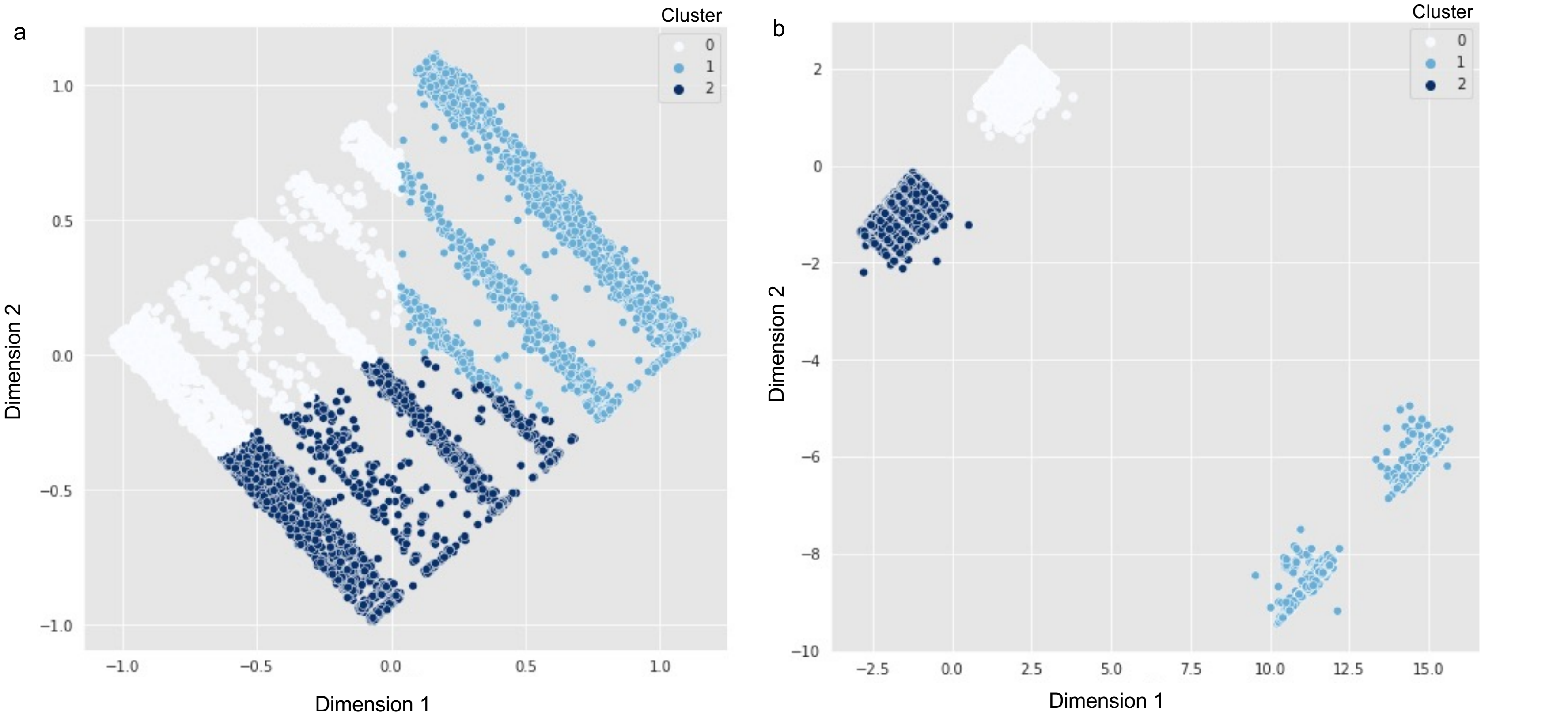
a
Cluster
0
1
2
1.0
0.5
0.0
−0.5
−1.0
Dimension 2
−1.0
−0.5
0.0
0.5
1.0
Dimension 1
b
Cluster
0
1
2
2
0
−2
−4
−6
−8
−10
Dimension 2
−2.5
0.0
2.5
5.0
7.5
10.0
12.5
15.0
Dimension 1

Click here to access/download;Figure;palomino-fig-3.pdf

Figure 3

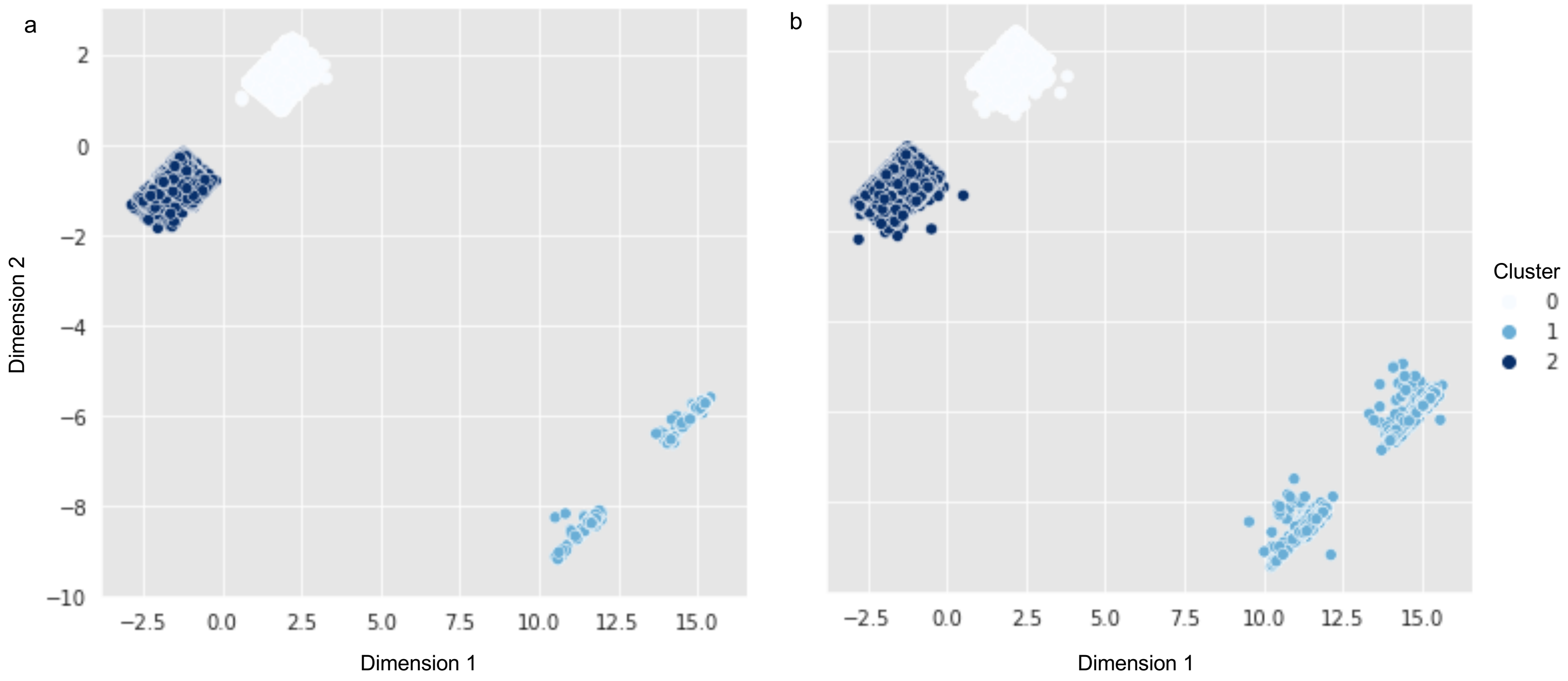

Figure 4

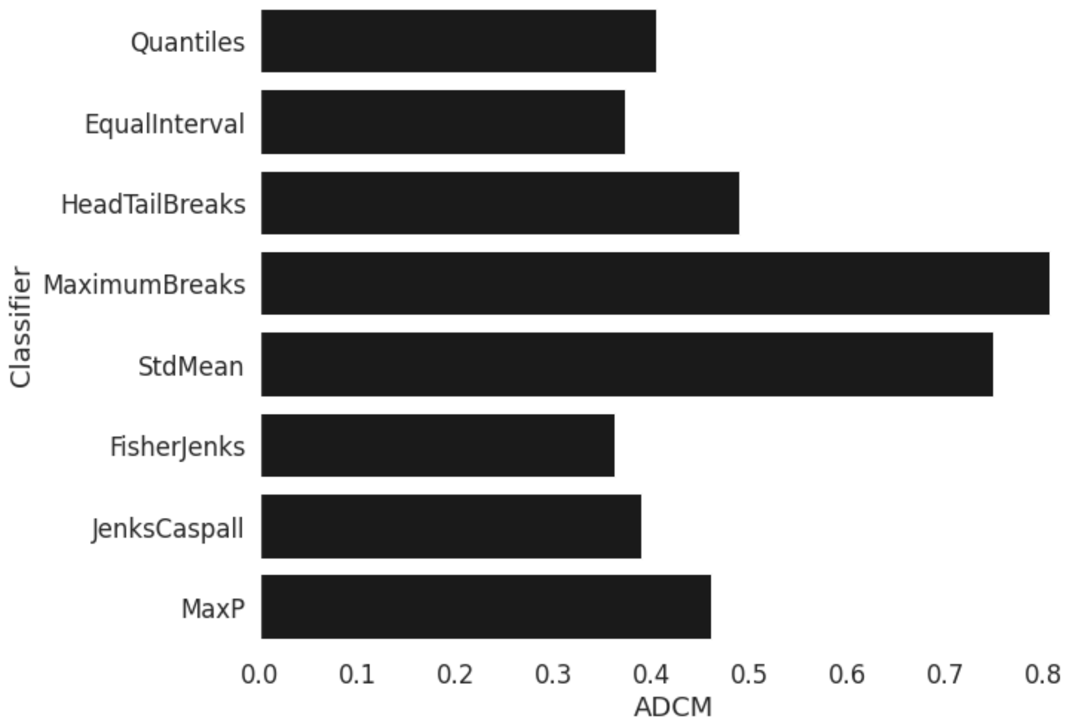
Quantiles
EqualInterval
HeadTailBreaks
MaximumBreaks
StdMean
FisherJenks
JenksCaspall
MaxP
Classifier
0.0
0.1
0.2
0.3
0.4
0.5
0.6
0.7
0.8
ADCM

Click here to access/download;Figure;palomino-fig-5.pdf

Figure 5

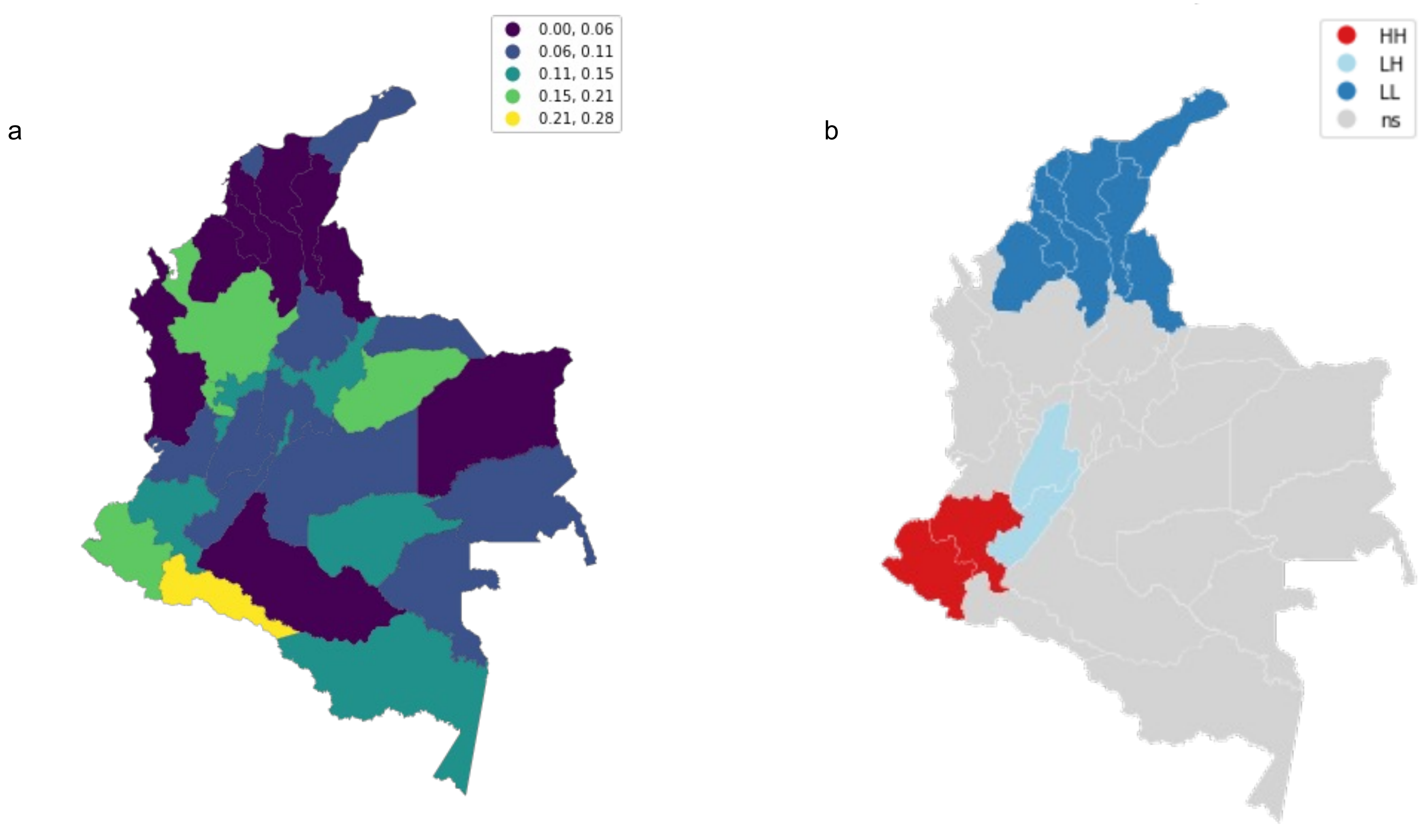

Click here to access/download;Figure;palomino-fig-6.pdf

Figure 6

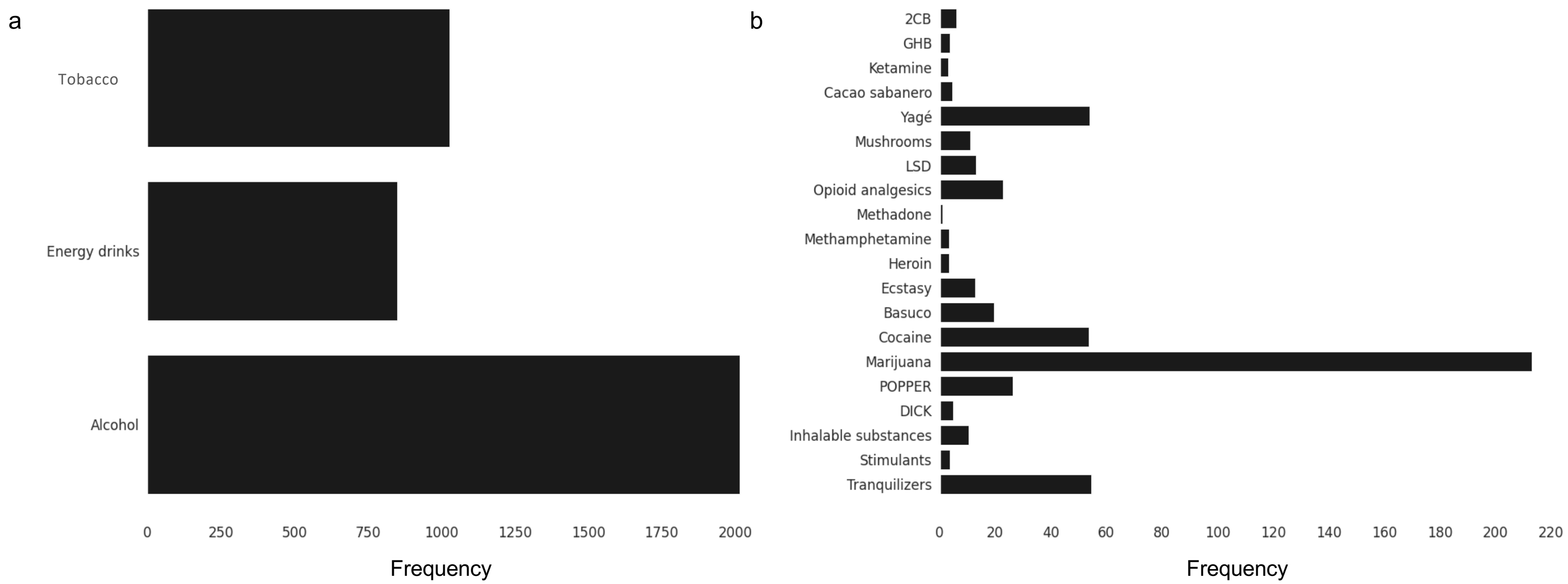

Click here to access/download;Figure;palomino-fig-7.pdf

Figure 7

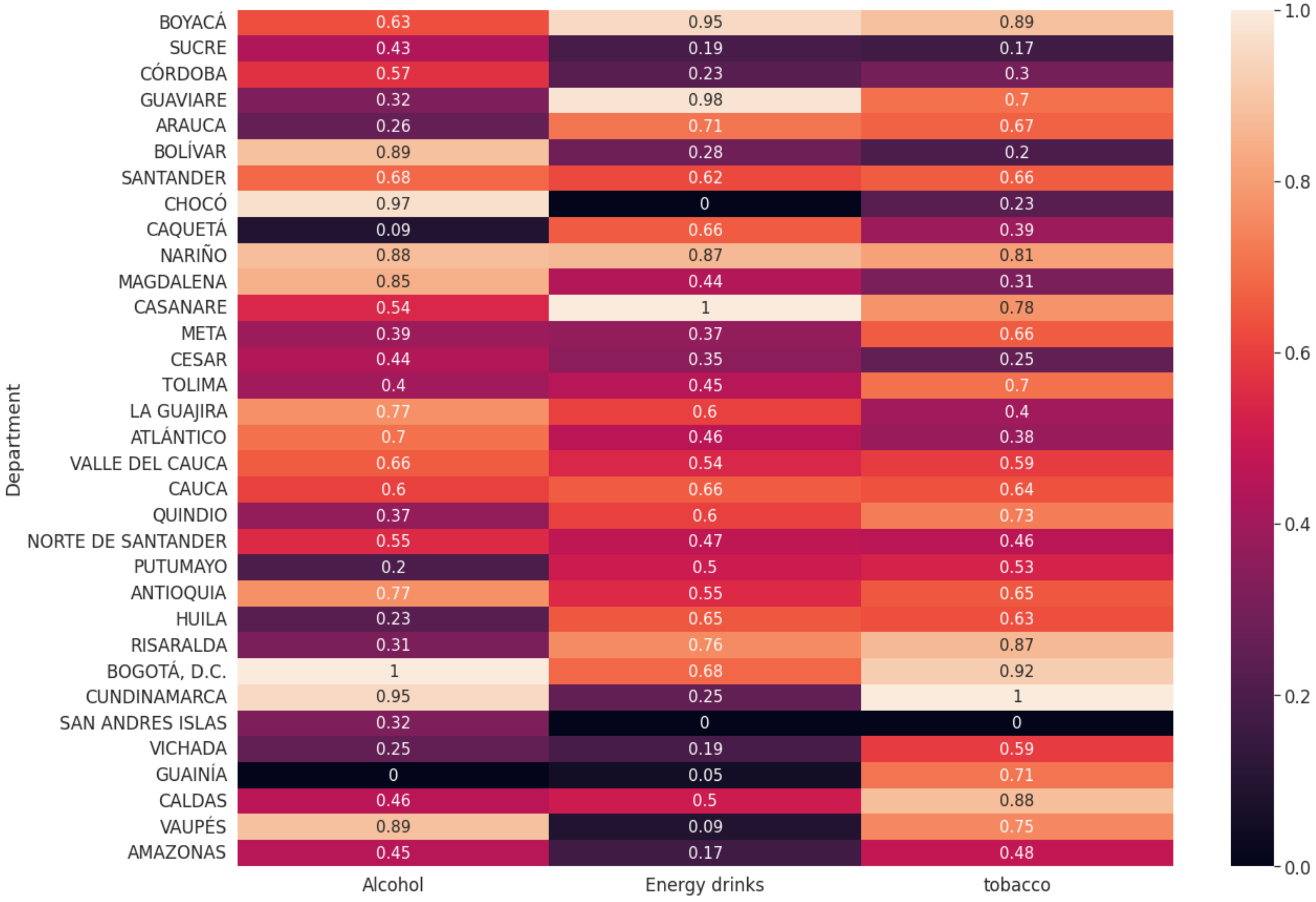

Click here to access/download;Figure;palomino-fig-8.pdf

Figure 8

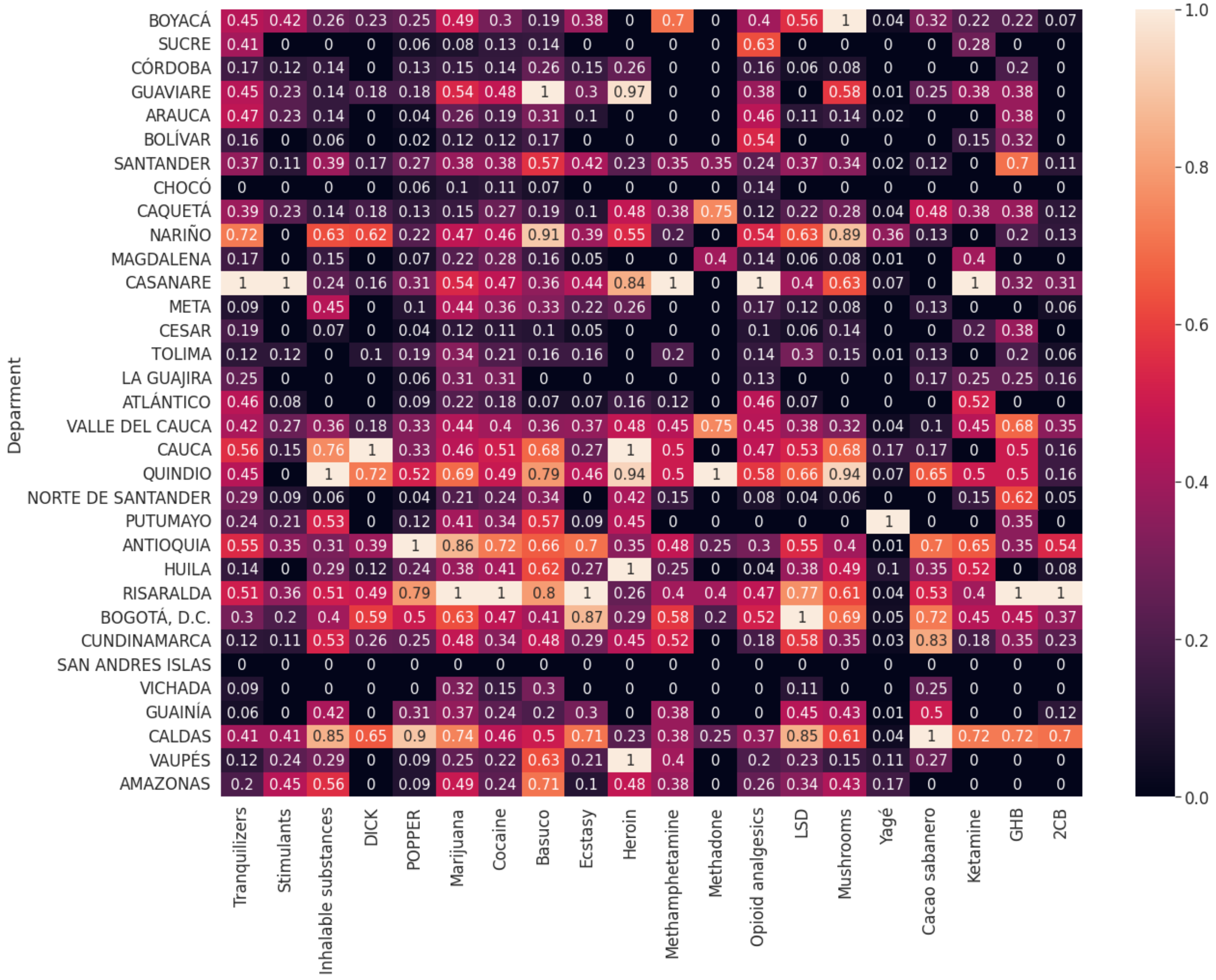

Click here to access/download;Figure;palomino-fig-9.pdf

Figure 9

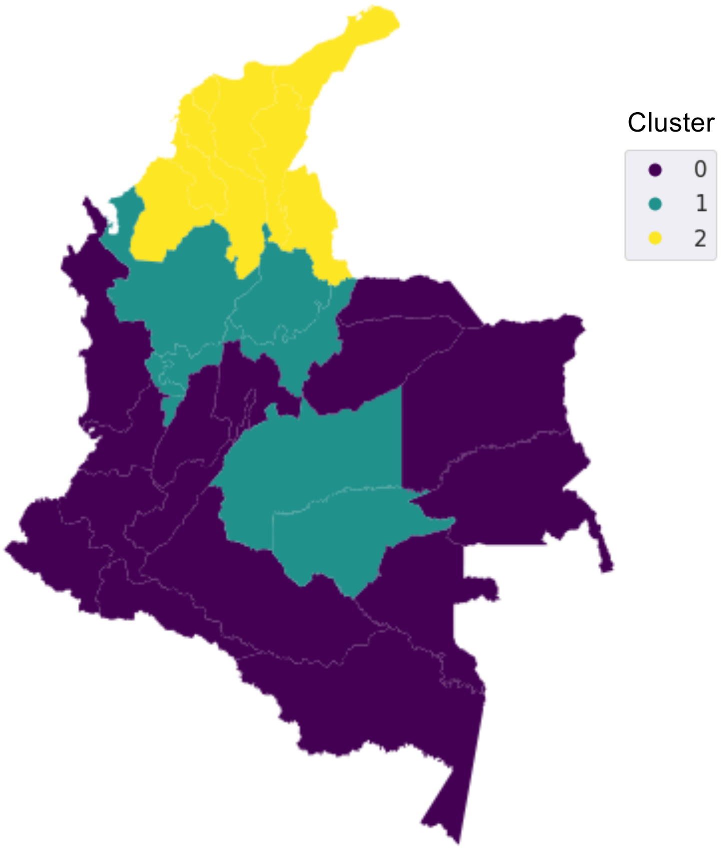

Supplementary materials

Click here to access/download
Supporting Information
Supplementary materials.docx
