# Supplementary material for: Leading consumption patterns of psychoactive substances in Colombia: A deep neural network-based clustering-oriented embedding approach
Source: PLoS One. 2023 Aug 18;18(8):e0290098. doi: 10.1371/journal.pone.0290098 (PMC10438020; doi:10.1371/journal.pone.0290098)
Supplement: Attachment — Submitted filename: PONE-D-22-28262_R1.pdf [file pone.0290098.s009.pdf]

PLOS ONE

Leading Consumption Patterns of Psychoactive Substances in Colombia: A Deep Neural Network-based Clustering-oriented Embedding Approach

--Manuscript Draft--

**Manuscript Number:**	PONE-D-22-28262R1
**Article Type:**	Research Article
**Full Title:**	Leading Consumption Patterns of Psychoactive Substances in Colombia: A Deep Neural Network-based Clustering-oriented Embedding Approach
**Short Title:**	Leading Consumption Patterns of Psychoactive Substances in Colombia
**Corresponding Author:**	kevin palomino<br>Universidad del Norte<br>Barranquilla, COLOMBIA
**Keywords:**	autoencoder; machine learning; spatial analysis; Drug consumption/abuse
**Abstract:**	The number of health-related incidents caused using illegal and legal psychoactive substances (PAS) has dramatically increased over two decades worldwide. In Colombia, the use of illicit substances has increased up to 10.3%, while the consumption alcohol and tobacco has increased to 84% and 12%, respectively. It is well-known that identifying drug consumption patterns in the general population is essential in reducing overall drug consumption. However, existing approaches do not incorporate Machine Learning and/or Deep Data Mining methods in combination with spatial techniques. To enhance our understanding of mental health issues related to PAS and assist in the development of national policies, here we present a novel Deep Neural Network-based Clustering-oriented Embedding Algorithm that incorporates an autoencoder and spatial techniques. The primary goal of our model is to identify general and spatial patterns of drug consumption and abuse, while also extracting relevant features from the input data and identifying clusters during the learning process. As a test case, we used the largest publicly available database of legal and illegal PAS consumption comprising 49,600 Colombian households. We estimated and geographically represented the prevalence of consumption and/or abuse of both PAS and non-PAS, while achieving statistically significant goodness-of-fit values. Our results indicate that region, sex, housing type, socioeconomic status, age, and variables related to household finances contribute to explaining the patterns of consumption and/or abuse of PAS. Additionally, we identified three distinct patterns of PAS consumption and/or abuse. At the spatial level, these patterns indicate concentrations of drug consumption in specific regions of the country, which are closely related to specific geographic locations and the prevailing social and environmental contexts. These findings can provide valuable insights to facilitate decision-making and develop national policies targeting specific groups given their cultural, geographic, and social conditions.
**Order of Authors:**	kevin palomino
	Carmen R. Berdugo
	Jorge I. Vélez
**Response to Reviewers:**	Dr. Vinicius Silva Belo<br>Academic Editor, PLOS ONE<br><br>RE: Revised Manuscript # PONE-D-22-28262<br><br>Dear Dr. Silva,<br><br>Thank you for the opportunity to submit a revised version of our manuscript, "Leading Consumption Patterns of Psychoactive Substances in Colombia: A Deep Neural Network-based Clustering-oriented Embedding Approach," which now includes responses to the concerns, inquiries, comments, and suggestions raised by two

Powered by Editorial Manager® and ProduXion Manager® from Aries Systems Corporation

anonymous reviewers. Please find below our response (in blue).

We very much appreciate all your efforts as Editor-in-Chief and the detailed and extraordinary revision this manuscript had. We enjoyed the thorough review of our manuscript, and it was a pleasure to respond to the reviewers. Please, Dr. Silva, allows us to mention that this exercise of the fair and exigent peer-review process is disappearing, and only good journals like PLOS ONE keep it. We hope that our new version might be suitable for publication in PLOS ONE.

Thank you very much for your time and consideration.

Yours sincerely,
Kevin R. Palomino, PhD(c)
Corresponding author

Comments from the Editor

1. Please ensure that your manuscript meets PLOS ONE's style requirements, including those for file naming. The PLOS ONE style templates can be found at https://journals.plos.org/plosone/s/file?id=wjVg/PLOSOne_formatting_sample_main_body.pdf and https://journals.plos.org/plosone/s/file?id=ba62/PLOSOne_formatting_sample_title_authors_affiliations.pdf
We very much appreciate your comments. We have updated our manuscript according to the journal’s formatting. In particular,
We changed the figure citation, i.e., from “Figure” to “Fig”.
We added the Numbered Equation.
We added the supporting information captions.
We changed the Cite references into brackets.
2. Please confirm that all data sources you used were publicly available and anonymized. Furthermore, please clarify how the data were accessed for the purpose of this study.
Thank you very much for your comments. The data is fully available and anonymized, and can be retrieved from https://microdatos.dane.gov.co/index.php/catalog/680/data-dictionary
For practicality, we downloaded the data and stored it on the cloud. The data can be retrieved from https://acortar.link/ktgCD9
3. In the ethics statement in the manuscript and in the online submission form, please provide additional information about the patient records/samples used in your retrospective study. Specifically, please ensure that you have discussed whether all data/samples were fully anonymized before you accessed them and/or whether the IRB or ethics committee waived the requirement for informed consent. If patients provided informed written consent to have data/samples from their medical records used in research, please include this information.
Thank you for your comment. As per Colombian regulations, the data were fully anonymized by the Colombian government before we could use them.
4. Thank you for stating the following financial disclosure:
"The author(s) received no specific funding for this work."
At this time, please address the following queries:
a) Please clarify the sources of funding (financial or material support) for your study. List the grants or organizations that supported your study, including funding received from your institution.
K.P. is a doctoral student at Universidad del Norte, Barranquilla, Colombia, and received a Ph.D. scholarship from this institution to cover tuition expenses. However, Universidad del Norte did not provide any additional funds for this study.
b) State what role the funders took in the study. If the funders had no role in your study, please state: “The funders had no role in study design, data collection and analysis, decision to publish, or preparation of the manuscript.”
Thank you for your comment. The following statement was included in the revised version of the manuscript to address this:
“The funders had no role in study design, data collection and analysis, decision to

Powered by Editorial Manager® and ProduXion Manager® from Aries Systems Corporation

publish, or preparation of the manuscript".

c) If any authors received a salary from any of your funders, please state which authors and which funders.
As stated previously, we did not receive any salary from organizations.
d) If you did not receive any funding for this study, please state: "The authors received no specific funding for this work."
Done. We have added the following statement in the revised version of the manuscript: "The authors received no specific funding for this work".
Please include your amended statements within your cover letter; we will change the online submission form on your behalf.
4. Thank you for stating the following in the Acknowledgments Section of your manuscript:
"K.P. is a doctoral student at Universidad del Norte, Barranquilla, Colombia, and received a Ph.D. scholarship from this institution. Some of this work is to be presented to the Ph.D. program in partial fulfilment of the requirements for the Ph.D. degree. The sponsor of the study has no role in study design; in the collection, analysis, and interpretation of data; in the writing of the report; and in the decision to submit the article for publication."
We note that you have provided funding information that is not currently declared in your Funding Statement. However, funding information should not appear in the Acknowledgments section or other areas of your manuscript. We will only publish funding information present in the Funding Statement section of the online submission form.
Please remove any funding-related text from the manuscript and let us know how you would like to update your Funding Statement. Currently, your Funding Statement reads as follows:
"The author(s) received no specific funding for this work."
Please include your amended statements within your cover letter; we will change the online submission form on your behalf.
We appreciate your correction. As per your request, we have removed any funding-related text from the manuscript and clarified that Universidad del Norte provided only tuition expenses for our PhD student.
5. In your Data Availability statement, you have not specified where the minimal data set underlying the results described in your manuscript can be found. PLOS defines a study's minimal data set as the underlying data used to reach the conclusions drawn in the manuscript and any additional data required to replicate the reported study findings in their entirety. All PLOS journals require that the minimal data set be made fully available. For more information about our data policy, please see http://journals.plos.org/plosone/s/data-availability.
Upon re-submitting your revised manuscript, please upload your study's minimal underlying data set as either Supporting Information files or to a stable, public repository and include the relevant URLs, DOIs, or accession numbers within your revised cover letter. For a list of acceptable repositories, please see http://journals.plos.org/plosone/s/data-availability#loc-recommended-repositories. Any potentially identifying patient information must be fully anonymized.
Important: If there are ethical or legal restrictions to sharing your data publicly, please explain these restrictions in detail. Please see our guidelines for more information on what we consider unacceptable restrictions to publicly sharing data: http://journals.plos.org/plosone/s/data-availability#loc-unacceptable-data-access-restrictions. Note that it is not acceptable for the authors to be the sole named individuals responsible for ensuring data access.
We will update your Data Availability statement to reflect the information you provide in your cover letter.
Thank you!
6. We note that Figures 1, 5 9 and S2 in your submission contain map images which may be copyrighted. All PLOS content is published under the Creative Commons Attribution License (CC BY 4.0), which means that the manuscript, images, and Supporting Information files will be freely available online, and any third party is permitted to access, download, copy, distribute, and use these materials in any way, even commercially, with proper attribution. For these reasons, we cannot publish previously copyrighted maps or satellite images created using proprietary data, such as Google software (Google Maps, Street View, and Earth). For more information, see our copyright guidelines: http://journals.plos.org/plosone/s/licenses-and-copyright.

Powered by Editorial Manager® and ProduXion Manager® from Aries Systems Corporation

	We require you to either (1) present written permission from the copyright holder to publish these figures specifically under the CC BY 4.0 license, or (2) remove the figures from your submission:<br><br>a. You may seek permission from the original copyright holder of Figures 1, 5, 9 and S2 to publish the content specifically under the CC BY 4.0 license.<br><br>We recommend that you contact the original copyright holder with the Content Permission Form (http://journals.plos.org/plosone/s/file?id=7c09/content-permission-form.pdf) and the following text:<br>“I request permission for the open-access journal PLOS ONE to publish XXX under the Creative Commons Attribution License (CCAL) CC BY 4.0 (http://creativecommons.org/licenses/by/4.0/). Please be aware that this license allows unrestricted use and distribution, even commercially, by third parties. Please reply and provide explicit written permission to publish XXX under a CC BY license and complete the attached form.”<br>Please upload the completed Content Permission Form or other proof of granted permissions as an ""Other"" file with your submission.<br><br>In the figure caption of the copyrighted figure, please include the following text: “Reprinted from [ref] under a CC BY license, with permission from [name of publisher], original copyright [original copyright year].”<br>We are very appreciative of your comments.<br>Figures containing maps were created using the plot function from GeoPandas (https://geopandas.org/en/stable/), which is an open-source library in Python. This library has a permissive license similar to the BSD 2-Clause License, and it has permissions for distribution, private use, modification, and commercial use (see https://github.com/geopandas/geopandas/blob/main/LICENSE.txt).<br>On the other hand, when creating all maps, we used a shape file that contains the location’s geometry. This shapefile was retrieved from https://hub.arcgis.com/datasets/de0e829ddbf743c895ba6dcee1b74fae/about. According to the author, this file can be freely accessible and used, which indicates that neither a license nor permission is needed to use it (i.e., https://hub.arcgis.com/datasets/de0e829ddbf743c895ba6dcee1b74fae/about).<br><br>b. If you are unable to obtain permission from the original copyright holder to publish these figures under the CC BY 4.0 license or if the copyright holder’s requirements are incompatible with the CC BY 4.0 license, please either i) remove the figure or ii) supply a replacement figure that complies with the CC BY 4.0 license. Please check copyright information on all replacement figures and update the figure caption with source information. If applicable, please specify in the figure caption text when a figure is similar but not identical to the original image and is therefore for illustrative purposes only.<br>The following resources for replacing copyrighted map figures may be helpful:<br><br>USGS National Map Viewer (public domain): http://viewer.nationalmap.gov/viewer/<br>The Gateway to Astronaut Photography of Earth (public domain): http://eol.jsc.nasa.gov/sseop/clickmap/<br>Maps at the CIA (public domain): https://www.cia.gov/library/publications/the-world-factbook/index.html and https://www.cia.gov/library/publications/cia-maps-publications/index.html<br>NASA Earth Observatory (public domain): http://earthobservatory.nasa.gov/<br>Landsat: http://landsat.visibleearth.nasa.gov/<br>USGS EROS (Earth Resources Observatory and Science (EROS) Center) (public domain): http://eros.usgs.gov/#<br>Natural Earth (public domain): http://www.naturalearthdata.com/<br>Kindly see our response to Comment #6. As mentioned above, a license to use the maps is not required.<br>7. Please include captions for your Supporting Information files at the end of your manuscript, and update any in-text citations to match accordingly. Please see our Supporting Information guidelines for more information: http://journals.plos.org/plosone/s/supporting-information.

Powered by Editorial Manager® and ProduXion Manager® from Aries Systems Corporation

We very much appreciate your supporting comments. We have updated our manuscript according to the journal's formatting.
We added the Supporting Information captions in the manuscript after references.
We separated the supplementary materials into four (4) files: S1_Table, S2_Table2, S3_Fig, and S4_Fig.
We changed the Supporting Information citation on the manuscript i.e. "Table 1S, supplementary material" to "S1 Table".

Comments from Reviewer #1
The manuscript was well constructed. However, the methodology seems very technical, with no explanation of what data were used, how and where the analyzes were carried out. I suggest making the methodology clearer, for example in CAE not only citing authors who used the technique but making it clear why it was chosen and not another deep learning technique.
We very much appreciate the reviewer's comments.
We used data from the Colombian National Survey on Psychoactive Substance Consumption. Data were extracted from https://microdatos.dane.gov.co/index.php/catalog/680/data-dictionary. Analyses were performed using Python's libraries GeoPandas (https://geopandas.org/en/stable/), TensorFlow (https://www.tensorflow.org/), PyTorch (https://pytorch.org/) and scikit-learn (https://scikit-learn.org/stable/), among others. All notebooks and code written for processing and analyzing the data are available from first author under reasonable request.
Following the reviewer's suggestion, we have modified the methodology in the revised version of the manuscript. We added a subtitle in the methodology denominated "Framework evaluation."
Now, the text reads:
"2.8 Framework Evaluation.
We trained the CAE-DEC method using data retrieved from the National Survey of Psychoactive Substance Consumption (DANE-DIMPE-ENCSPA-2019), which contains 49,600 observations. A second database with PAS production figures, was used in the spatial analysis stage to correlate the PSA consumption and production. In order to evaluate the framework, we compared our CAE-DEC approach with other approaches, including CAE, and Principal Component Analysis integrated with clustering (PCA-DEC). For evaluation and comparison purposes, we use the Calinski-Harabasz [76], Davies-Bouldin [77], and Silhouette [78] index as intrinsic clustering metrics. In addition, we used the χ^2 statistic to investigate potential associations and differences among the patterns (clusters) identified using our approach."
On the other hand, we rewrote a paragraph in the methodology to make clear why the model was chosen. Now, the text reads:
"Fig 1 presents the proposed Convolutional Auto-Encoder- Deep Embedded Clustering (CAE-DEC) framework based on the implementation presented by Xie et al. [57]. However, unlike the Xie et al. model, our structure is developed by applying convolutional layers for the deep autoencoder (DA) architecture instead of a linear one to represent high-order interactions in the data. In addition, a spectral clustering-based centroid estimation is proposed to achieve an improved initial centroid calculation. We chose the CAE-DEC framework based on its ability to reduce both the number of model parameters and the dimensionality, while creating clusters simultaneously."
"
In the results, improve the identification of tables and figures. Include subtitles to make files more understandable.
Thank you for your comment. As suggested and following the Journal's formatting for Figures and Table file naming, we have improved the tables and figures captions in the revised version of the manuscript (i.e., from "Palomino-fig-1" to "Fig1").

In the Discussion, explore further the impact of the results and assess the environmental issue of these sites. There was a stratification of regions and consumption profile, but there is no information about the location. The area is urban or rural, what level of development. Perhaps these and other local (environmental) aspects may be related and/or favour the consumption of these substances.
Thank you for your suggestion. We improved the discussion section accordingly in the revised version of the manuscript. The relevant text now reads:

"In this study, we propose and test a Deep Neural Network-based Clustering-oriented

Powered by Editorial Manager® and ProduXion Manager® from Aries Systems Corporation

Embedding algorithm (i.e., a ML-based model) for identifying psychoactive substance (PAS) use and abuse patterns in Colombia. This model allows the automatic extraction of features from the input data (such as sex, age, socioeconomic status, and housing type) to determine whether an individual has consumed PAS. It then creates clusters in the new data space generated during the learning process, following the methods outlined in [57, 59]. After the training process, a latent feature space (LFS) is generated and the results are subsequently analysed. We have identified clearly marked clusters where the prevalence of individuals who use or do not use PAS is notable. Additionally, we found that region, sex, housing type, socioeconomic strata, age, and whether individuals contribute to household finances have a statistically significant impact on the clustering structure. These findings are consistent with previous studies aimed at identifying PAS consumption patterns [19, 80, 81]. Interestingly, when comparing the CAE-DEC model proposed in this study and the CAE-Spectral model using different metrics (i.e., Silhouette statistic, which measures the internal density of each cluster and the distance that separates them from each other, the Calinski-Harabasz index and the Davies-Bouldin index [DBI]), we found that our model performs better (Silhouette: 0.62 vs. 0.786; Calinski-Harabasz: 22468.26 vs. 775992.45; DBI: 0.2898 vs. 0.63; S2 Table, Supplementary Material).

Based on our findings, individuals more likely to consume PAS are grouped in cluster 2, while cluster 1 consisted of individuals who did not consume PAS (Table 1). Not surprisingly, a significant proportion of females characterizes cluster 1. In addition, most individuals belong to socioeconomic strata 1, are 40 years old or older, and do not contribute economically to support their household. In contrast, cluster 2 is characterized by a higher proportion of males aged between 20 and 40 in socioeconomical strata 1 and 2, who do not contribute to the household finances (Table 1). Finally, cluster 0 is characterized by a small proportion of males, a higher proportion of individuals in strata 3, 4, 5, and 6, and individuals are more likely to contribute to the household economy (Table 1).

At the level of spatial statistics, we identified that legal drugs such as alcohol have a high prevalence in all regions of Colombia, with a slight tendency to more consumption in coastal areas (Fig 7). In our country, the coastal areas are often popular tourist destinations, and many tourists come to these areas looking for a relaxing experience, which can increase alcohol consumption. Coastal areas typically have warmer temperatures and more sunshine, increasing thirst and making people more likely to consume alcohol. Additionally, bars, clubs, and restaurants serve alcoholic beverage due to the high demand from tourists and locals [82, 83]. Another characteristic of this area is the fishing and maritime culture. This culture is often associated with hard work and long working hours, and alcohol may be seen as a way to relax and unwind after a tough day at the sea [84]. Finally, this region has 68% urban and 32% rural zones [60]. The level of development, as measured by gross domestic product (GDP), is the third region with significant economic development in the country [85] (S3 Table, Supplementary Material). Interestingly, the consumption of illegal drugs is lower in the Northern region than in other regions of the country. However, there is a more representative consumption of non-prescription tranquilizers, opioids, ketamine, GHB, and heroin. In particular, the Atlántico department has the highest consumption proportion within this region (Fig 8).

Tobacco consumption is present in all regions, with a higher proportion in the Central region, where climate conditions resemble temperate weather. Also, this region has a diverse consumption pattern, where drugs such as marijuana, popper, cocaine, ecstasy, inhalants, methadone, heroin, LSD, GHB, 2CB, and mushrooms prevail. This region has Colombia's largest cities (i.e., Bogotá and Medellin); Bogotá has the highest population density and is a hub for drug trafficking routes, while Medellín has an unfortunate history of drug cartels and gang violence. Ultimately, this region is comprised of 77% urban areas, and the most developed cities in the country are located there [60, 85] (S3 Table, Supplementary Material). Energy drinks are more frequently used in the Eastern region, characterized by a continental climate surrounded by flat territory. Our results are in line with the scientific literature suggesting that the location of regions within countries is directly associated with the consumption of PAS [26, [86–88]. The consumption of heroin, basuco, non-prescription tranquilizers, stimulants, methamphetamines, opioids, and ketamine characterizes this region. This zone is the second most developed region in the country, and 72% of

urban areas [60], [85](S3 Table, Supplementary Material).Our findings also show that the Southern region is more likely to consume illegal drugs, including basuco, heroin, and Yagé (Fig 8). One of the main reasons for this result is that, unfortunately, this region has favourable environmental characteristics (i.e., majority rainforest) for their consumption and production, being the second largest illegal drug-producing region in Colombia [79]. Furthermore, this region has the highest percentage of rurality (52%) compared to the other regions, and its level of development is low as measured by the GDP [60, 85] (S3 Table, Supplementary Material).

In the Western region, also known as the Pacific region, consumption mostly mainly includes of Methylene Chloride (DICK), GHB, heroin, opioids, and methamphetamines. This region (Pacific) is mainly characterized known for its geographical isolation, poverty, and ongoing conflict, which have contributed to the growth of drug production and trafficking in the area. Poverty is one of the main factors driving drug production in the Pacific region, which has led many people to turn to drug cultivation and trafficking for survival. Additionally, the region's rugged terrain and limited infrastructure have made it difficult for the Colombian government to establish a strong presence, allowing drug traffickers to operate with relative impunity [89]. This region has a similar percentage of urban (53%) and rural (47%) populations than the Southern region, and ranks second among the regions with the lowest levels of development (S3 Table, Supplementary Material). In the Western region, also known as the Pacific region, consumption mainly includes methylene chloride, GHB, heroin, opioids, and methamphetamines. This region is mainly characterized for its geographical isolation, poverty, and ongoing conflict, which have contributed to the growth of drug production and trafficking in the area. Poverty is one of the main factors driving drug production in the Pacific region, which has led many people to turn to drug cultivation and trafficking for survival. Additionally, the region's rugged terrain and limited infrastructure have made it difficult for the Colombian government to establish a strong presence, allowing drug traffickers to operate with relative impunity [89]. This region has a similar percentage of urban (53%) and rural (47%) populations than the Southern region, and ranks second among the regions with the lowest levels of development (S3 Table, Supplementary Material).[89]. This region has a similar percentage of urban (53%) and rural (47%) populations than the Southern region. On the other hand, this region ranks second among the regions with the lowest levels of development (S3 Table, Supplementary Material)."

Comments from Reviewer #2

Abstract should rewrite and included technical approach more.
Done.

page 5 "Feature selection approaches for clustering can be split into filter, wrapper, embedded," are these methods just for clustering!!?
Thank you for your comment.
No, feature selection approaches do not only apply for clustering algorithms.
Feature selection is the process of selecting a subset of relevant features (variables and/or attributes) to be used in a supervised or unsupervised model and constitutes an important step in Machine Learning as including irrelevant or redundant features in a model can lead to overfitting, decreased model performance, and increased computational complexity. In this sense, feature selection approaches can be useful for classification, clustering, or regression.

In the Introduction, please give problem, challenges clearly. Introduction is written separately.
Thank you for your input. Following your advice, several changes have been made in the Introduction of the revised version of the manuscript to address this.

In introduction did not cite many reverences together" IDEC (Guo et al., 2017), DEPICT (Dizaji et al., 2017), DBC (Li et al., 2017), DualAAE (Ge et al., 2020), VAED (Lim et al., 2020), and DNC (B. Li et al., 2021) "
Done.
What is difference between convolutional auto-encoder (CAE) and stacked space auto encoder?
Both Convolutional Autoencoder (CAE) and Stacked Autoencoder (SAE) are types of

autoencoders, a type of neural network architecture that is used for unsupervised learning and data compression.

The main difference between CAE and SAE is the way they handle the input data. A CAE is typically used for processing image data. It uses convolutional layers to extract spatial features from the input image and then uses deconvolutional layers to reconstruct the image. CAEs are well-suited for image data because they can capture the spatial relationships between pixels in an image and can learn to recognize visual patterns and shapes. On the other hand, SAE is typically used for processing structured or unstructured data. It consists of multiple layers of neural networks that encode the input data into a lower-dimensional representation and then decode it back to the original dimensions. SAEs are useful for feature learning and data compression in many different types of data, including text, audio, and structured data.

In summary, while CAE is focused on processing image data using convolutional layers, SAE can be applied to various types of data and uses multiple layers of neural networks for encoding and decoding.

Did not compare other previous works.
Thank you for the comments. In the supplementary section, S2 Table shows the comparison of our model with PCA-K-means, and CAE-Spectral.

Table S2. Performance metrics for different models.
Performance metricCAE-DECPCA-K-meansCAE-Spectral
Calinski-Harabasz775992.45128651.8322468.26
Davies-Bouldin0.28980.5670.63
Silhouette0.7860.60610.62
In this sense, S2 Table shows that our CAE-DEC model results in the highest Calinski-Harabasz score, which means that identified clusters are dense and well separated. On the other hand, our model has the smallest Davies-Bouldin score, which indicates that identified clusters groups have a better partition. Regarding the Silhouette index, our model gives the highest value, which implies that clusters are highly dense.
Performance metric interpretation
Calinski-Harabasz score: A high score indicates that clusters are dense and well separated.
Davies-Bouldin score: Lower is better. Lower values indicating better clustering.
Silhouette index: The best value is 1 and the worst value is -1. Values near 0 indicate overlapping clusters. Negative values generally indicate that a sample has been assigned to the wrong cluster, as a different cluster is more similar.

Your problem is classification or clustering?

We appreciate your question.

The problem we are addressing is this study is a clustering problem, as we aim to identify consumption patterns of psychoactive substances (PAS) in the Colombian territory. In particular, we used clustering techniques (i.e., an ensemble model that integrates an autoencoder and cluster method) to find the different patterns of PAS consumptions in Colombian citizens considering the consumers’ location.

Please give your performance metrics
The performance metrics are stated in the Discussion of the manuscript. The relevant text reads:

“Interestingly, when comparing the CAE-DEC model proposed herein and the CAE-Spectral model using different score metrics (i.e., Silhouette score, which measures the internal density of each cluster and the distance that separates them from each other, the Calinski-Harabasz index and the Davies-Bouldin index [DBI]) showed that the our proposal model performs better (Silhouette: 0.62 vs. 0.786; Calinski-Harabasz: 22468.26 vs. 775992.45; DBI: 0.2898 vs. 0.63) than the CAE-Spectral alone model”

More information about performance metrics can be found in S2 Table of the Supplementary Material. According to our results, the proposed CAE-DEC model shows a well-separated and highly dense cluster, meaning we can define better groups

Powered by Editorial Manager® and ProduXion Manager® from Aries Systems Corporation

	and identify PAS consumer patterns more precisely.
Additional Information:	
Question	Response
**Financial Disclosure**<br><br>Enter a financial disclosure statement that describes the sources of funding for the work included in this submission. Review the submission guidelines for detailed requirements. View published research articles from *PLOS ONE* for specific examples.<br><br>This statement is required for submission and **will appear in the published article** if the submission is accepted. Please make sure it is accurate.<br><br>**Unfunded studies**<br>Enter: *The author(s) received no specific funding for this work.*<br><br>**Funded studies**<br>Enter a statement with the following details:<br>• Initials of the authors who received each award<br>• Grant numbers awarded to each author<br>• The full name of each funder<br>• URL of each funder website<br>• Did the sponsors or funders play any role in the study design, data collection and analysis, decision to publish, or preparation of the manuscript?<br>• **NO** - Include this sentence at the end of your statement: *The funders had no role in study design, data collection and analysis, decision to publish, or preparation of the manuscript.*<br>• **YES** - Specify the role(s) played.<br><br>* typeset	The author(s) received no specific funding for this work.
**Competing Interests**<br><br>Use the instructions below to enter a competing interest statement for this submission. On behalf of all authors, disclose any competing interests that could be perceived to bias this work—acknowledging all financial support and any other relevant financial or non-financial competing interests.	The authors have declared that no competing interests exist.

This statement is required for submission and **will appear in the published article** if the submission is accepted. Please make sure it is accurate and that any funding sources listed in your Funding Information later in the submission form are also declared in your Financial Disclosure statement.<br><br>View published research articles from *PLOS ONE* for specific examples.<br><br>**NO authors have competing interests**<br><br>Enter: *The authors have declared that no competing interests exist.*<br><br>**Authors with competing interests**<br><br>Enter competing interest details beginning with this statement:<br><br>*I have read the journal's policy and the authors of this manuscript have the following competing interests: [insert competing interests here]*<br><br>* typeset	
**Ethics Statement**<br><br>Enter an ethics statement for this submission. This statement is required if the study involved:<br><br>• Human participants<br>• Human specimens or tissue<br>• Vertebrate animals or cephalopods<br>• Vertebrate embryos or tissues<br>• Field research<br><br>Write "N/A" if the submission does not require an ethics statement.<br><br>General guidance is provided below. Consult the submission guidelines for detailed instructions. **Make sure that all**	This research does not require an ethics statement approved by an ethics committee since we are using public information where there is no identification or individual information of the people involved.

Powered by Editorial Manager® and ProduXion Manager® from Aries Systems Corporation

**information entered here is included in the Methods section of the manuscript.**<br><br>**Format for specific study types**<br><br>**Human Subject Research (involving human participants and/or tissue)**<br>• Give the name of the institutional review board or ethics committee that approved the study<br>• Include the approval number and/or a statement indicating approval of this research<br>• Indicate the form of consent obtained (written/oral) or the reason that consent was not obtained (e.g. the data were analyzed anonymously)<br><br>**Animal Research (involving vertebrate animals, embryos or tissues)**<br>• Provide the name of the Institutional Animal Care and Use Committee (IACUC) or other relevant ethics board that reviewed the study protocol, and indicate whether they approved this research or granted a formal waiver of ethical approval<br>• Include an approval number if one was obtained<br>• If the study involved *non-human primates*, add *additional details* about animal welfare and steps taken to ameliorate suffering<br>• If anesthesia, euthanasia, or any kind of animal sacrifice is part of the study, include briefly which substances and/or methods were applied<br><br>**Field Research**<br><br>Include the following details if this study involves the collection of plant, animal, or other materials from a natural setting:<br>• Field permit number<br>• Name of the institution or relevant body that granted permission	
**Data Availability**<br><br>Authors are required to make all data underlying the findings described fully available, without restriction, and from the time of publication. PLOS allows rare exceptions to address legal and ethical	Yes - all data are fully available without restriction

concerns. See the PLOS Data Policy and FAQ for detailed information.<br><br>A Data Availability Statement describing where the data can be found is required at submission. Your answers to this question constitute the Data Availability Statement and **will be published in the article**, if accepted.<br><br>**Important:** Stating 'data available on request from the author' is not sufficient. If your data are only available upon request, select 'No' for the first question and explain your exceptional situation in the text box.<br><br>Do the authors confirm that all data underlying the findings described in their manuscript are fully available without restriction?	
**Describe where the data may be found in full sentences. If you are copying our sample text, replace any instances of XXX with the appropriate details.**<br><br>• If the data are **held or will be held in a public repository**, include URLs, accession numbers or DOIs. If this information will only be available after acceptance, indicate this by ticking the box below. For example: *All XXX files are available from the XXX database (accession number(s) XXX, XXX.).*<br>• If the data are all contained **within the manuscript and/or Supporting Information files**, enter the following: *All relevant data are within the manuscript and its Supporting Information files.*<br>• If neither of these applies but you are able to provide **details of access elsewhere**, with or without limitations, please do so. For example:<br><br>*Data cannot be shared publicly because of [XXX]. Data are available from the XXX Institutional Data Access / Ethics Committee (contact via XXX) for researchers who meet the criteria for access to confidential data.*	The data used in the manuscript were obtained from a third party, the Archivo Nacional de Datos (ANDA), and are fully available and anonymized. The authors confirm that others would be able to access these data in the same manner as themselves; and the authors did not have any special access privileges that others would not have. The data can be publicly retrieved from ANDA (https://microdatos.dane.gov.co/index.php/catalog/680/data-dictionary).

Powered by Editorial Manager® and ProduXion Manager® from Aries Systems Corporation

The data underlying the results presented in the study are available from (include the name of the third party and contact information or URL).<br>• This text is appropriate if the data are owned by a third party and authors do not have permission to share the data.<br><br>* typeset	
Additional data availability information:	

Powered by Editorial Manager® and ProduXion Manager® from Aries Systems Corporation

Revised Manuscript
Click here to access/download;Manuscript;Revised Manuscript.docx

Leading Consumption Patterns of Psychoactive Substances in Colombia: A Deep Neural Network-based Clustering-oriented Embedding Approach

Kevin Palomino[1,*], Carmen R. Berdugo[1], Jorge I. Vélez[1]

[1] *Department of Industrial Engineering, Universidad del Norte, Barranquilla, Colombia.*

[*] Corresponding author. E-mail: krpalomino@uninorte.edu.co

ORCIDs

KP: https://orcid.org/0000-0002-2708-1096
CB: https://orcid.org/0000-0002-4803-2816
JIV: https://orcid.org/0000-0002-3146-7899

Abstract

The number of health-related incidents caused using illegal and legal psychoactive substances (PAS) has dramatically increased over two decades worldwide. In Colombia, the use of illicit substances has increased up to 10.3%, while the consumption alcohol and tobacco has increased to 84% and 12%, respectively. It is well-known that identifying drug consumption patterns in the general population is essential in reducing overall drug consumption. However, existing approaches do not incorporate Machine Learning and/or Deep Data Mining methods in combination with spatial techniques. To enhance our understanding of mental health issues related to PAS and assist in the development of national policies, here we present a novel Deep Neural Network-based Clustering-oriented Embedding Algorithm that incorporates an autoencoder and spatial techniques. The primary goal of our model is to identify general and spatial patterns of drug consumption and abuse, while also extracting relevant features from the input data and identifying clusters during the learning process. As a test case, we used the largest publicly available database of legal and illegal PAS consumption comprising 49,600 Colombian households. We estimated and geographically represented the prevalence of consumption and/or abuse of both PAS and non-PAS, while achieving statistically significant goodness-of-fit values. Our results indicate that region, sex, housing type, socioeconomic status, age, and variables related to household finances contribute to explaining the patterns of consumption and/or abuse of PAS. Additionally, we identified three distinct patterns of PAS consumption and/or abuse. At the spatial level, these patterns indicate concentrations of drug consumption in specific regions of the country, which are closely related to specific geographic locations and the prevailing social and environmental contexts. These findings can provide valuable insights to facilitate decision-making and develop national policies targeting specific groups given their cultural, geographic, and social conditions.

***Keywords*:** Autoencoder, Machine Learning, Spatial analysis, Drug consumption/abuse.

Author summary

Why was this study done?

- Mental disorders caused by psychoactive substances (PAS) have a high global disease burden and result in significant costs to society.
- Interventions for PAS consumers currently follow international protocols and do not consider the individual or regional characteristics.
- Targeted interventions for specific groups based on their cultural, geographic, and social conditions are needed.

What did the researchers do and find?

- We developed a novel georeferenced mathematical model to identify PAS consumption patterns, including spatial analysis to emphasize the need for georeferenced interventions.
- Our model identified and characterized three groups of PAS consumers based on location and sociodemographic information.

What do these findings mean?

- Design and focus interventions on identified clusters to reduce negative impact of PSA drug consumption in specific areas.
- Future modelling studies could explore new patterns related to medical information.
- Policies for treating patients with high PAS consumption should consider the identified patterns in each region.

1. Introduction

Psychoactive substances (PAS) are chemical substances that change the function of the nervous system and cause alterations in people's perception, mood, consciousness, cognition, or behavior [1]. PAS can be grouped according to their chemical structure as synthetic cannabinoids, synthetic cathinones, phenethylamines, arylcyclohexylamines, tryptamines, indolalkylamines, new synthetic opioids, piperazines, ketamine, and designer benzodiazepines. They can also be grouped according to their origin as a natural origin or synthetic molecules [2–4].

The increased numbers of drug use among young people are drawing the attention of national governments [5]. Because the number of health-related incidents caused by using legal and illegal PAS worldwide has dramatically increased over the last two decades [2, 6], this phenomenon has become some of the largest burdens of disease [7, 8]. Drug use constitutes a high cost to society due to premature mortality, increased health expenditure, criminal justice (drug and micro-trafficking), social welfare costs, and other social consequences [9, 10].

Colombia is ranked as one of the largest drug producers in the world [11]. Unfortunately, the production and commercialization of drugs through drug- and micro-trafficking, constantly expands in locations with high levels of poverty and limited government presence [12]. Statistics and indicators of drug consumption, production, and distribution, as well as reports from the National Statistical System (DANE) of Colombia, highlight a dramatic increase in (1) drug production [13]; (2) intern consumption of PAS at early ages; and (3) the prevalence in use and/or abuse of drugs have dramatically increased over the last 20 years [14, 15]. Furthermore, the country also has the highest prevalence of drug use among school students in recent years compared to other Latin American countries [16]. Thus, there is an urgent need to develop effective interventions to prevent the use and/or abuse of PAS.

The first step towards reducing this consumption is to identify drug consumption patterns in the general population [17]. Several studies have identified patterns associated with drug use and consumption [18, 19]. According to the Center for Disease Control and Prevention, individuals who

do not have their own homes and live in rented accommodations are more likely to use drugs [20]. Other research studies suggest that neighborhood contextual characteristics may increase the risk of substance abuse [21–26]. Additionally, population density may also influence substance use and overdose risk through a higher level of socialization in densely populated urban areas [27–29]. Other authors have identified that anxiety, sleep disorders, suicide, depression, and other mental illnesses are risk factors for the consumption and abuse of PAS [30, 31]. Furthermore, early marijuana use has been shown to increase the risk of consuming other PAS [32]. Furthermore, people involved in sports and artistic activities perceive drugs as enhancers element for improving their performance [33].

In Colombia, drug consumption patterns and risk factors have also been identified. For instance, Kalyanam et al. [34] analyzed the social impact of basuco and inhalant use among street youths. Narvaez-Chicaiza [35] assessed the social factors that lead to the adoption of harm reduction policies and how these factors influence treatments for substance abuse disorders. Additionally, Restrepo-Escobar & Cardona [36] demonstrated that university students with low satisfaction in their studies tend to be heavy users of alcohol, tobacco, and marijuana. However, these approaches do not consider the use of Machine Learning (ML) and/or Deep Data Mining techniques in combination with spatial models to analyze drug consumption data from the general population. To our knowledge, we have not found any robust models integrating ML and spatial models to identify drug consumption patterns using publicly available Colombian databases.

Although several techniques for analyzing drug consumption patterns are currently available (i.e., ML, Bayesian, spatial, traditional multivariate, or univariate statistical models, or, in some cases, a combination of these), new trends in pattern identification and analysis techniques focus on hybrid and ensemble models [37]. Currently, the most widely used ML techniques are Support Vector Machines (SVMs), Random Forest (RF), and Natural Language Processing (NLP)[38–42]. Among Bayesian models, Bayesian meta-regression (DisMod-MR), Bayesian hierarchical models, and Markov Chain Monte Carlo are the most attractive methods [43–45]. Regarding spatial models,

Spatial Distributions, Spatial Regression Models, Spatial Scan Statistics, Variograms, and Social Mapping are the most frequently used techniques [46–49]. On the other hand, logistic regression, confirmatory factor analysis, and correlational analysis are the most employed traditional statistical models to identify drug-associated patterns [50–53].

Fraley and Raftery [54] suggest separating clustering approaches into hierarchical and partitioning techniques. Partitioning techniques are divided into density-, model-, and grid-based methods, the most popular of which are *K*-means, PAM, CLARA, DBSCAN and CLIQUE. On the other hand, hierarchical techniques are divided into agglomerative and divisive methods. Of these, the best-known methods are BRICH, CURE, ROCK, and CHAMELEON (see [55] for further reading). Although these techniques have been shown to perform well when relevant features are removed *a priori*, it is well-known that in clustering algorithms, irrelevant and redundant features in the data may degrade the quality of clusters and lead to high computational cost. Therefore, removing such features may alleviate these issues. Thus, we focus on identifying patterns of PAS consumption using an ensemble model integrating an autoencoder with both a clustering algorithm and a spatial model. As part of our approach, we used the most recent and representative works for data clustering, and different dimensionality reduction and feature selection methodologies proposed in the literature.

Feature selection approaches in clustering can be split into filter, wrapper, embedded, and hybrid approaches [37]. While wrappers depend on the clustering algorithms to evaluate the clustering quality of a selected feature subset, filters are independent of the clustering algorithm. Embedded approaches also work with a clustering algorithm and, unlike wrappers, incorporate knowledge about the clustering structure. Another type of method is hybrid approaches, which combine filter and wrapper approaches into a single strategy. However, studies on embedded and hybrid feature selection approaches in clustering are limited [37]. Other feature learning-based approaches using Deep Neural Networks have been shown to work well for linear and nonlinear models [56]. For instance, Xie et al. in (2016) [57] propose to work on feature extraction and

clustering using pre-trained Auto-Encoders simultaneously. However, these are mainly used to work and process images. In general, deep clustering models use Auto-Encoders since they can learn input features without labels on the data; performance measures show that this approach is reliable for different data types [58]. Thus, deep clustering methods have become a growing field of research for feature selection [58]. In this regard, the use of convolutional networks in autoencoders and the application of feature selection for clustering are open questions that have not been fully addressed yet, especially when dealing with data from different statistical distributions [37].

Here, we propose a Deep Neural Network-based Clustering-oriented Embedding Algorithm that allows us to (*i*) identify consumption patterns of PAS; and (*ii*) build an ensemble algorithm integrating an autoencoder with a clustering algorithm and a spatial model to deal with the feature space and cluster memberships. Our approach is based on the model proposed by Xie et al. [57] and B. Li et al. [59], and expands their work by creating an autoencoder from a convolutional network to represent high-order interactions in the data accurately and simultaneously incorporate a spatial analysis to describe drug consumption patterns properly. Our main hypothesis is that incorporating these two critical elements in our proposal will help to identify and better understand drug consumption patterns and support national policy development processes.

2. Materials and methods

2.1 Study area

Located in South America, the Republic of Colombia is a diverse country with a population of over 50 million people distributed over a territory of 440,831 square miles [60], encompassing jungles, highlands, grasslands, deserts, coasts, and islands, distributed in six regions and 32 departments (states)[61]. It is worth noting that, unfortunately, Colombia has been a major producer of illegal drugs for a long time, which has had a significant impact on drug consumption and abuse.

According to the United Nations Office on Drugs and Crime, Colombia is the first cocaine-producing country and the eighth country with the highest production of cannabis [62]. In addition, the Colombian Drug Observatory indicates that the use of illicit substances in the territory has increased to 10.3%, with men between the ages of 18 and 24 being the heaviest consumers of these types of drugs. Reports also indicate that consumption of licit substances such as alcohol and tobacco has recently increased dramatically [63].

2.2 Data sources

We used two databases to identify drug consumption patterns in Colombia. The first database was retrieved from the 2019 National Survey of Psychoactive Substance Consumption in the General Population (DANE-DIMPE-ENCSPA-2019; URL: https://microdatos.dane.gov.co/index.php/catalog/680/data-dictionary) conducted by the National Statistical System (DANE) of Colombia [64]. This survey includes observations of 49,600 households, where information on housing, location, general characteristics of individuals, consumption of legal and illegal PAS, and implemented treatments is registered. The second database comes from the Colombian Drug Observatory and contains information on the production of PAS per area during 2019. All these databases are fully available and completely anonymized. In this study, we used departments (states) as georeferenced areas using polygons (i.e., a shapefile) as

implemented in ArcGIS Hub [65]. Thus, an ethics statement approved by an ethics committee is not required since we are using public information without the identification or individual information of the people involved.

2.3 Convolutional Auto-Encoder-Deep Embedded Clustering Algorithm

Fig 1 presents the proposed Convolutional Auto-Encoder- Deep Embedded Clustering (CAE-DEC) framework based on the implementation presented by Xie et al. [57]. However, unlike the Xie et al. model, our structure is developed by applying convolutional layers for the deep autoencoder (DA) architecture instead of a linear one to represent high-order interactions in the data. In addition, a spectral clustering-based centroid estimation is proposed to achieve an improved initial centroid calculation. We chose the CAE-DEC framework based on its ability to reduce both the number of model parameters and the dimensionality, while creating clusters simultaneously.

Fig 1. Architecture of the proposed CAE-DEC model. Republished from [65] under a CC BY license, with permission from [ArcGIS Hub], original copyright [2016].

In our approach, an encoder structure is first applied to map the input vector into a lower feature space, called latent feature space (LFS). Then, the LFS is independently passed through a decoder structure and a clustering layer to achieve an efficient clustering framework. The encoder-decoder combination (DA) attempts to extract a LFS preserving the relevant information from the original input data. On the other hand, the clustering layer seeks to execute an improved clustering assignment by minimizing the divergence between a target distribution and a centroid-based probability distribution.

In the last stage of the framework, a spatial analysis was performed using the feature space generated from the autoencoder as input. Here, the spatial data exploration is initially performed

using Global Spatial Autocorrelation to determine to which level the similarity between observations in a dataset relates to the similarity of the locations of such observations [66]. To assess GSA, the Moran's I [67], Geary's *C* [68], and Getis and Ord's *G* [69] statistics are estimated. We also measure the Local Spatial Autocorrelation, which focuses on the relationships between each observation and its surroundings, rather than providing a single-number summary of these relationships across the map [70]. This is estimated based on the ability to determine whether spatial autocorrelation is present in a geographically referenced data set. Finally, we perform regionalization, which corresponds to a special kind of clustering where the objective is to group similar observations based on their statistical attributes and spatial location [71]. In this sense, regionalization embeds the same logic as standard clustering techniques while applying a series of geographical constraints [72]. This framework was built using the TensorFlow (https://www.tensorflow.org/) and PyTorch (https://pytorch.org/) libraries in Python version 3.11 [73].

2.4 Convolutional Auto-Encoder (CAE)

The DA is a deep neural network architecture capable of learning unsupervised representations of an input data set. Typically, DA networks are used for dimensionality reduction or denoising tasks. The structure of a DA is based on two deep networks: a network to transform the original input data into a latent feature space, and a network trained to reconstruct the original input data using the extracted latent space as input. The first network, used to extract the latent space, is called the encoder, while the second is called the decoder. Rather than using fully connected layers, the implemented DA architecture incorporates convolutional (CONV) layers and fully connected (FC) layers for LFS extraction and reconstruction (**Fig 1**). Integrating convolutional layers in a DA is also called CAE [74]. Compared to a DA, which is built with only fully connected layers, the CAE structure can reduce the number of parameters compared to a DA [75].

2.4.1 Convolutional layer

The proposed CAE structure is designed using four convolutional layers, two CONV layers during the encoder stage, two CONV layers during the decoder stage, and two fully connected layers (**Fig 1**). The convolution operation can be denoted as:

$$z_{i,j,k}^{l} = W_k^{l^T} X_{i,j}^{l} + b_k^{l} \quad (1)$$

where $z_{i,j,k}^{l}$ is the value of each feature at the (i, j) location in the k-th feature map of the l-th layer, W_k^l and b_k^l represent the weight and bias of the k-th filter of the l-th layer, and $X_{i,j}^{l}$ denotes the input value at location (i, j) of the l-th layer. For non-linear mapping, an activation function $g(.)$ is applied over the convolutional feature $z_{i,j,k}^{l}$ as follows:

$$a_{i,j,k}^{l} = g(z_{i,j,k}^{l}) \quad (2)$$

where $a_{i,j,k}^{l}$ is the activation value resulting from applying the activation function $g(.)$. The Rectified Linear Unit (ReLU) function is set as the activation function on each CONV, except in the final decoder CONV layer where a sigmoidal activation is applied.

2.5 Clustering layer

The clustering layer is inspired by Xie et al.[57]. Initially, a soft assignment is computed between the latent space, also known as embedded space, and the cluster centroids. Then, update steps are repeated to define the final cluster centroids and embedded space. The Kullback–Leibler (KL) divergence is used as loss function during the optimization procedure. The objective is to minimize de KL divergence between a soft clustering distribution Q and an auxiliar target distribution P. The KL loss is calculated as:

$$L_c = KL(P \| Q) = \sum_k \sum_m p_{km} log\left(\frac{p_{km}}{q_{km}}\right) \quad (3)$$

where L_c is the clustering loss. To measure the similarity between embedded point z_k and the cluster centroid c_m, the t Student's distribution is used as a kernel:

$$q_{km} = \frac{(1 + \|z_k - c_m\|^2/\alpha)^{-\frac{\alpha+1}{2}}}{\sum_i (1 + \|z_k - c_i\|^2/\alpha)^{-\frac{\alpha+1}{2}}} \quad (4)$$

with α the degrees of freedom of the *t* Student's distribution and q_{km} is a soft clustering assignment distribution of each embedded point (i.e., probability of assigning point *k* to cluster *m*). As in Xie et al., when setting α=1 the similarity function q_{km} can be calculated as:

$$q_{km} = \frac{(1 + \|z_k - c_m\|^2)^{-1}}{\sum_i (1 + \|z_k - c_i\|^2)^{-1}} \quad (5)$$

To compute the target distribution p_{km}, the second power of q_{km} is calculated, and a cluster normalization is applied as follows:

$$p_{km} = \frac{q_{km}^2 / \sum_k q_{km}}{\sum_i (q_{ki}^2 / \sum_k q_{ki})} \quad (6)$$

Then, by minimizing the divergence between *P* and *Q*, the embedding learning is achieved through highly confident assignments.

2.5.1 Center initialization

As previously mentioned, the cluster centroids are initialized using a spectral clustering-based approach. The spectral clustering allows flexible distance metrics and provides better cluster estimations than *K*-means [57]. However, most spectral clustering algorithms have high computational requirements. To overcome these computational requirements, random samples are taken to estimate the cluster centroids. As spectral clustering does not estimate any centroid during the learning process, once the clusters are defined, the mean of each cluster is used as the centroid estimator.

2.6 The CAE-DEC model

Initially, the input data is normalized within the interval [0, 1]. This normalization allows the network to use the most advanced learning rate and avoid the vanishing gradient problems, as well as alleviate overfitting. Further, to achieve a better learning process, the last CONV layer in the decoder structure is activated by a sigmoid activation function. Then, two training steps will be executed during the CAE-DEC learning process. Firstly, a CAE model will be trained to minimize the reconstruction loss L_r computed as

$$L_r = \|x - \tilde{x}\|^2 \tag{7}$$

where x is the normalized input and $\tilde{x}$ is the reconstructed output. This pretrained CAE model is then used as the DA structure in the CAE-DEC model.

In the second step, the CAE-DEC model is trained to simultaneously minimize reconstruction loss and clustering loss. The total loss during this training step will be set as

$$L_t = L_r + C \cdot L_c \tag{8}$$

where L_r is the CAE-DEC reconstruction loss, L_c is the CAE-DEC clustering loss, and C is a coefficient to control the loss balance. The training process is shown in **Table 1**. The goal is to obtain a latent space that minimizes the total loss. Finally, the label of each embedded point is established as

$$Label_j = \arg max_m q_{jm} \tag{9}$$

Table 1. Pseudo code for the CAE-DEC training process.

```
Pseudo code: The CAE-DEC training process
Input data: Number of clusters n; Normalized input data x; Maximum number of iterations Mint; Balance
coefficient C; Pretrained CAE; Stop condition Stop; Target distribution P update condition P_change.
Training process:
1. Generate an initial latent space (Z) through the pre-trained CAE
2. Run spectral clustering with Z to generate the initial cluster centers (C)
3. Initialize the CAE-DEC model with the pretrained CAE.
4. Calculate soft assignment distribution Q and target distribution P based on Z and C
for epoch<Minter do:
    if epoch%P_change==0 then:
        Calculate soft assignment distribution Q and target distribution P based on Z and C
    end if
    Feed the CAE-DEC with the normalized input data x
    Calculate the reconstruction loss and the clustering loss
    Update CAE-DEC parameters. Weight, Bias, and Centers.
    if Stop==True then:
        Break
```

end if
end for
Obtain the label for each data point from the las optimized Q.
Output: Latent space, labels

where q_{jm} is the probability that point j belongs to a specific cluster center m. On the other hand, the maximum number of iterations Mint and the target distribution P update condition P_change was chosen based on multiple experiments. The final Mint and P_change values were 3000 and 5, respectively. This final P_change improves stability during the training process.

2.7 Framework Evaluation

We trained the CAE-DEC method using data retrieved from the National Survey of Psychoactive Substance Consumption (DANE-DIMPE-ENCSPA-2019), which contains 49,600 observations. A second database with PAS production figures, was used in the spatial analysis stage to correlate the PSA consumption and production. In order to evaluate the framework, we compared our CAE-DEC approach with other approaches, including CAE, and Principal Component Analysis integrated with clustering (PCA-DEC). For evaluation and comparison purposes, we use the Calinski-Harabasz [76], Davies-Bouldin [77], and Silhouette [78] index as intrinsic clustering metrics. In addition, we used the χ^2 statistic to investigate potential associations and differences among the patterns (clusters) identified using our approach.

3. Results

3. 1 Model Comparison for Identifying Drug Consumption Patterns

Fig 2 depicts the LFS resulting after applying the CAE and CAE-DEC models to the data. Among all individuals, we identified three different clusters; 14935 (30.19%) individuals belong to cluster 0, 11528 (23.30%) individuals belong to cluster 1, and 23005 (46.50%) individuals belong to cluster 2. Interestingly, the LFS generated with the CAE-DEC has more defined clusters than the CAE model. Although the CAE model seeks to extract a LFS that preserves the essential characteristics of the input data, our proposed CAE-DEC model not only preserves these important characteristics but, at the same time, also forces the encoder structure to generate representative clusters while extracting the new feature space.

On the other hand, the reconstruction loss obtained through the CAE model is higher than that of the CAE-DEC model. This result may be related to the fact that the CAE-DEC model used the pre-trained CAE model during its construction. It should be noted that the CAE model alone cannot determine the labels of each point or define clusters in the data. Thus, clusters in **Fig 2** were obtained through spectral clustering and were the bases for initializing the centroids in the CAE-DEC model.

Fig 2. Derived Latent Feature Space based on the (a) CAE and (b) CAE-DEC models.

3.2 Identification of Clusters of Psychoactive Drugs Consumption

Here we analyse the patterns in each cluster obtained using the CAE-DEC model. We defined a priority dummy variable Y_{ij} quantifying whether the *i*th person in household *j*th has consumed PAS; $Y_{ij} = 1$ when an individual has never consumed PAS and $Y_{ij} = 2$ otherwise. Out of the 49468 individuals in the sample, only 5514 (11.15%) consume PAS. **Fig 3a** and **Fig 3b** depict, respectively, the derived cluster structure for individuals consuming PAS and those who reported

not consuming, derived from the CAE-DEC model. Our results indicate that individuals in clusters 0 and 2 are more likely to consume some PAS (**Fig 3a**), while most individuals in cluster 1 do not (**Table 2**). In particular, 1726 (11.56%) individuals in cluster 0, 392 (3.4%) individuals in cluster 1, and 3396 (14.76%) individuals in cluster 2 have used PAS (**Table 2**). A χ^2-based test of independence reveals that the region where individuals are located, age (years), the type of household they live in, their socioeconomic status (SES), and whether they contribute to the household finances are statistically significantly associated with the cluster they belong to (**Table 2**).

Fig 3. Resulting clusters for individuals (a) consuming and (b) not consuming psychoactive substances based on the CAE-DEC model.

Table 2. Distribution of demographic and social variables across clusters.

Variables		Cluster 0 (*n*=14935)	Cluster 1 (*n*=11528)	Cluster 2 (*n*=23005)	χ^2	*df*	*P*-value
Region	Caribbean	3004	2991	4075	1472.9	10	<.0001
	Central-Eastern	2526	2299	6175			
	Central-Southern	1843	1299	1702			
	Eje Cafetero – Antioquia	3902	2354	6371			
	Llanos Orientales	1687	1305	1496			
	Pacific	1973	1280	3186			
Gender	Male	6606	3927	10233	386.72	2	<.0001
	Female	8329	7601	12772			
Housing type	House	8250	6538	11834	114.18	6	<.0001
	Apartment	6275	4724	10595			
	Room	395	256	543			
	Indigenous dwelling	15	10	33			
Socioeconomic status	1	3889	3626	6945	843.92	10	<.0001
	2	4627	3902	8851			
	3	4284	2867	5683			
	4	1341	718	979			
	5	510	256	362			
	6	284	159	185			
Age (years)	(0, 20]	2105	1576	2998	345.87	4	<.0001
	(20, 40]	6814	4329	10889			
	(40, 68]	6016	5623	9118			
Contribute to the household finances	Yes	10128	7473	16041	85.24	2	<.0001
	No	4807	4055	6964			

df: Degrees of freedom.

Table 3 shows the adjusted residuals for our model. According to our results, the Central-Eastern region significantly contributes to the Region variable. In this region, the observed value is higher than the expected value in cluster 2, while the observed value is lower than the expected value for cluster 0. Although to a lesser extent, the Llanos Orientales region also significantly

contributes the χ^2 statistic. Indeed, this region shows fewer observed individuals than the expected number of individuals in cluster 2 and a higher number observed than expected individuals in clusters 0 and 1 (**Table 3**).

Table 3. Adjusted residuals comparing the observed and expected frequencies based on the cluster analysis.

	**Variables**	**Cluster 0 (*n*=14935)**	**Cluster 1 (*n*=11528)**	**Cluster 2 (*n*=23005)**
Region	Caribbean	-0.88	17.02	-13.61
	Central-Eastern	-18.72	-6.76	22.97
	Central-Southern	12.54	6.09	-16.7
	Eje Cafetero - Antioquia	2.02	-14.36	10.31
	Llanos Orientales	11.32	9.59	-18.55
	Pacific	0.84	-6.97	5.13
Gender	Male	6.68	-19.66	10.52
	Female	-6.68	19.66	-10.52
Housing type	House	4.17	7.13	-9.88
	Apartment	-4.83	-6.61	10.05
	Room	2.2	-1.54	-0.72
	Indigenous dwelling	-0.72	-1.09	1.59
Socioeconomic status	1	-10.26	5.99	4.37
	2	-12.72	-3.3	14.51
	3	9.14	-3	-5.87
	4	17.29	0.44	-16.29
	5	11.12	-0.49	-9.82
	6	8.26	1.2	-8.62
Age (years)	(0, 20]	2.54	0.61	-2.85
	(20, 40]	3.2	-17.23	11.66
	(40, 68]	-4.98	16.93	-9.77
Contribute to the household finances	Yes	-0.61	-8.37	7.65
	No	0.61	8.37	-7.65

On the other hand, Gender has a higher-than-expected value of males in clusters 0 and 2, while it is lower in cluster 1. For females, the opposite occurs in cluster 1, and lower values are observed in clusters 0 and 2. Similarly, Housing Type has a higher-than-expected value of individuals living at houses in cluster 1 and a lower-than-expected in cluster 2. Conversely, cluster 2 has more individuals living in apartments, and cluster 1 has the lowest (**Table 3**).

Regarding SES, a higher-than-expected number of individuals in strata 3, 4, 5, and 6 in cluster 0 were found (**Table 3**). We also observed a lower-than-expected number of individuals in strata 3, 4, 5, and 6 in cluster 2 and a higher-than-expected number in strata 1, 4 and 6 in cluster 1 (**Table 3**). Moreover, the age variable shows a higher-than-expected observed value for the (0,20] range in cluster 0. For ages between (20,40] years, cluster 2 has a higher-than-expected number of individuals. Conversely, there is a lower number of individuals in cluster 1. Finally, the household economy variable results show that cluster 2 has a higher-than-expected value of individuals contributing to the household finances, and cluster 1 has a lower-than-expected value of individuals not contributing to it. Comparison of Calinski-Harabasz, Davies-Bouldin, and silhouette metrics between a principal component analysis (PCA)-based deep autoencoder (PCA-DEC) and our proposed CAE-DEC model indicates the superiority of the latter (**Table S2**, **Supplementary Material**).

3.3 Spatial Analysis of Psychoactive Drugs Consumption

Different alternative classification algorithms were used to determine the number of choropleth class limits (i.e., Equal Intervals, Quantiles, Maximum Breaks, Box plot, Head-Tail Breaks, Jenks-Caspall, Fisher-Jenks, and Max-p) and compared using the absolute deviation around class medians optimization criterion (**Fig 4**). According to our results, the Fisher-Jenks classifier performed better and hence was selected.

Fig 4. Absolute deviation around class medians (ADCM) statistic criterion for different alternative classifiers. Here, lower is better.

Following the same exploratory spatial analysis, we constructed a choropleth with the percentage of PAS use for each of the 32 Colombian departments (**Fig 5a**). We found that the departments of Arauca, Vichada, Caquetá, Chocó, Magdalena, Cesar, Bolivar, Sucre, Cordoba, and Norte de Santander have low percentages of drug use. However, some of these departments are major drug producers (i.e., Cordoba and Guaviare), according to data from the Drug Observatory of Colombia [79]. Similarly, Putumayo is the department with the highest proportions of PAS use (**Fig 5a**).

Fig 5. (a) Consumption percentage of psychoactive substances according to the CAE-DEC model; (b) Moran's I statistic; (c) Moran's cluster map. Here, HH, LH, LL and ns represent high-high, low-high, low-low, and not statistically significant quadrants, respectively. This clustering pattern leads to a statistically significant Moran's I statistic of 0.2 (*P*-value <0.01). Architecture of the proposed CAE-DEC model. Republished from [65] under a CC BY license, with permission from [ArcGIS Hub], original copyright [2016].

The global Moran's I results show the presence of a statistically significant positive global spatial autocorrelation (I = 0.2005, $P<0.01$). Thus, the null hypothesis that the map is random (i.e., that the map shows more spatial patterns than we would expect if the values had been randomly assigned to a location) is rejected. In addition, other global indices such as Geary's C ($C = 0.693$, $P = 0.003$) and Getis and Ord's G ($G = 0.800$, $P = 0.049$) confirm the presence of statistically significant global spatial autocorrelation.

To further explore the relationships between each observation and its environment, the Local Indicators of Spatial Association (LISA) were estimated (more information on LISA statistics is provided in **Fig S2**, **Supplementary Material**). **Fig 5b** depicts the Moran diagram, indicating each quadrant's positive (or negative) association. Specifically, the high-high (HH) and low-low (LL) quadrants indicate a positive association between high and low drug use. On the other hand, the low-high (LH) and high-low (HL) quadrants indicate negative associations with drug use (**Fig 5b**). Following our results, we found that departments such as Nariño and Cauca belong to the HH cluster. In contrast, la Guajira, Atlántico, Magdalena, Cesar, Norte de Santander, Sucre, and Cordoba belong to the LL. This clustering pattern leads to a statistically significant Moran's I statistic (*P*-value <0.01). Thus, a little over 39.4% of the departments are considered, by this analysis, to be part of a spatial cluster (i.e., statistically significant with a *P*-value <5%). We also identified that, among legal drugs, alcohol and tobacco are the most frequently consumed in the national territory (**Fig 6a**). At the same time, marijuana, followed by non-prescription tranquilizers and Yagé, and a slight consumption of opioids and Poppers, are the most frequently consumed illegal drugs (**Fig 6b**).

Fig 6. Frequency of consumption of (a) legal and (b) illegal drugs in Colombia.

Regarding legal drugs, alcohol has the highest consumption rates in Bogotá, Cundinamarca, and Chocó (**Fig 7**). However, there is moderately high use in Vaupés, Nariño, Bolívar, Magdalena, La Guajira, and Atlántico. As for energy drinks, consumption is the highest in Casanare and Guaviare and has slightly high uses in Boyacá, Nariño, Risaralda, and Arauca. On the other hand, tobacco has the highest consumption in Cundinamarca but has moderately high uses in Bogotá, Boyacá, Nariño, Casanare, Tolima, Quindío, Risaralda, Guainía, Caldas, and Vaupés. It should be mentioned that the use of these drugs is also present across the country but with a lower incidence (**Fig 7**).

Fig 7. Consumption of illegal drugs by department. For interpretation purposes, number represents values scaled on a range of 0 to 1. For instance, Bogotá D.C. has the highest LSD consumption and Putumayo has the lowest.

Concerning illegal drugs, non-prescription tranquilizers and stimulants are most prevalent in Casanare (**Fig 8**). However, the consumption of tranquilizers is slightly higher in Nariño, while inhalants have the highest consumption in Quindío, followed by Cauca, Caldas, and Nariño. Methylene Chloride has the highest consumption in Cauca and a high consumption in Quindío and Nariño; Antioquia, followed by Caldas and Risaralda, shows the highest consumption of popper. On the contrary, marijuana has its highest consumption in Risaralda and moderately high consumption in Caldas, Bogotá, Antioquia, and Quindío. As for cocaine, its consumption is the highest in Risaralda and moderately high in Antioquia (**Fig 8**).

Fig 8. Consumption of legal drugs by department. Number represents values scaled on a range of 0 to 1 for psychoactive substance use. Conventions as in Fig 7.

On the other hand, basuco (i.e., cocaine paste) has the highest consumption rate in Guaviare, and critical consumption in Nariño, Cauca, Quindío, Antioquia, and Amazonas; ecstasy has its highest consumption in Risaralda, followed by Bogotá and Caldas; heroin consumption is highest in Vaupes, Huila, Cauca, Quindío, and Arauca, and is slightly higher in Casanare; methamphetamine consumption is highest in Casanare and is moderately high in Boyacá; methadone is most widely used in Quindío, but has slightly high levels of use in Valle del Cauca and Caquetá; opioids are most prevalent in Casanare, followed by Sucre; LSD is most prevalent in Bogotá, but has high levels of use in Caldas, Risaralda, Quindío, and Nariño; mushrooms have their highest consumption in Boyacá and have moderately high uses in Quindío, Risaralda, Bogotá, Cauca, and Casanare; Yagé has a higher incidence in Putumayo; cacao sabanero has its highest consumption in Caldas,

and has moderate consumption in Cundinamarca, Bogotá, Antioquia, and Quindío; ketamine has the highest consumption in Casanare, followed by Antioquia; and GHB has the highest consumption in Risaralda, followed by Santander, Valle del Cauca, and Norte de Santander. Finally, 2CB has the highest consumption rate in Risaralda, followed by Caldas. Although the consumption pattern of some departments is not mentioned, there is low and moderate consumption for certain drugs in some of them (**Fig 8**).

3.4 Regionalization of Clusters

We applied a regionalization method as a grouping technique for imposing a spatial restriction, i.e., the result of a regionalization algorithm contains clusters with geographically coherent areas and coherent data profiles. Our approach uses a spatially constrained hierarchical clustering algorithm, which identified three clusters representing the consumption of PAS in the country (**Fig 9**). The number of clusters was estimated based on the average silhouette indexes, the total intra-cluster variance, and dendrograms (**Fig S1**, **Supplementary Material**). Following our results, cluster 0 is comprised of departments such as La Guajira, Cesar, Atlántico, Magdalena, Norte de Santander, Bolivar, Sucre, and Cordoba, all of them located in the Northern region of the country; cluster 1 is comprised of Antioquia, Santander, Boyacá, Caldas, Risaralda, and Quindío; and cluster 2 is integrated by the remaining departments (**Fig 9**). When testing geographical coherence, which is the measure that assesses the "compactness" of a given shape, our results indicate that the clusters derived using the regionalization model represent moderately compact regions. In addition, the feature coherence (i.e., goodness-of-fit) test using different metrics showed that our 3-cluster regionalization structure properly fits the data (**Table S1**, **Supplementary Material**).

Fig 9. Cluster map of drug use after regionalization. Republished from [65] under a CC BY license, with permission from [ArcGIS Hub], original copyright [2016].

4. Discussion

In this study, we propose and test a Deep Neural Network-based Clustering-oriented Embedding algorithm (i.e., a ML-based model) for identifying psychoactive substance (PAS) use and abuse patterns in Colombia. This model allows the automatic extraction of features from the input data (such as sex, age, socioeconomic status, and housing type) to determine whether an individual has consumed PAS. It then creates clusters in the new data space generated during the learning process, following the methods outlined in [57, 59]. After the training process, a latent feature space (LFS) is generated, and the results are subsequently analysed.

We have identified clearly marked clusters where the prevalence of individuals who use or do not use PAS is notable. Additionally, we found that region, sex, housing type, socioeconomic strata, age, and whether individuals contribute to household finances have a statistically significant impact on the clustering structure. These findings are consistent with previous studies aimed at identifying PAS consumption patterns [19, 80, 81]. Interestingly, when comparing the CAE-DEC model proposed in this study and the CAE-Spectral model using different metrics (i.e., Silhouette statistic, which measures the internal density of each cluster and the distance that separates them from each other, the Calinski-Harabasz index and the Davies-Bouldin index [DBI]), we found that our model performs better (Silhouette: 0.62 vs. 0.786; Calinski-Harabasz: 22468.26 vs. 775992.45; DBI: 0.2898 vs. 0.63; S2 **Table**, **Supplementary Material**).

Based on our findings, individuals more likely to consume PAS are grouped in cluster 2, while cluster 1 consisted of individuals who did not consume PAS (**Table 2**). Not surprisingly, a significant proportion of females characterizes cluster 1. In addition, most individuals belong to socioeconomic strata 1, are 40 years old or older, and do not contribute economically to support their household. In contrast, cluster 2 is characterized by a higher proportion of males aged between 20 and 40 in socioeconomical strata 1 and 2, who do not contribute to the household finances (**Table 2**). Finally, cluster 0 is characterized by a small proportion of males, a higher proportion of

individuals in strata 3, 4, 5, and 6, and individuals are more likely to contribute to the household economy (**Table 2**).

At the level of spatial statistics, we identified that legal drugs such as alcohol have a high prevalence in all regions of Colombia, with a slight tendency to more consumption in coastal areas (**Fig 7**). In our country, the coastal areas are often popular tourist destinations, and many tourists come to these areas looking for a relaxing experience, which can increase alcohol consumption. Coastal areas typically have warmer temperatures and more sunshine, increasing thirst and making people more likely to consume alcohol. Additionally, bars, clubs, and restaurants serve alcoholic beverage due to the high demand from tourists and locals [82, 83]. Another characteristic of this area is the fishing and maritime culture. This culture is often associated with hard work and long working hours, and alcohol may be seen as a way to relax and unwind after a tough day at the sea [84]. Finally, this region has 68% urban and 32% rural zones [60]. The level of development, as measured by gross domestic product (GDP), is the third region with significant economic development in the country [85] (S3 **Table**, **Supplementary Material**). Interestingly, the consumption of illegal drugs is lower in the Northern region than in other regions of the country. However, there is a more representative consumption of non-prescription tranquilizers, opioids, ketamine, GHB, and heroin. In particular, the Atlántico department has the highest consumption proportion within this region (**Fig 8**).

Tobacco consumption is present in all regions, with a higher proportion in the Central region, where climate conditions resemble temperate weather. Also, this region has a diverse consumption pattern, where drugs such as marijuana, popper, cocaine, ecstasy, inhalants, methadone, heroin, LSD, GHB, 2CB, and mushrooms prevail. This region has Colombia’s largest cities (i.e., Bogotá and Medellin); Bogotá has the highest population density and is a hub for drug trafficking routes, while Medellín has an unfortunate history of drug cartels and gang violence. Ultimately, this region is comprised of 77% urban areas, and the most developed cities in the country are located there [60, 85] (S3 **Table**, **Supplementary Material**).

Energy drinks are more frequently used in the Eastern region, characterized by a continental climate surrounded by flat territory. Our results are in line with the scientific literature suggesting that the location of regions within countries is directly associated with the consumption of PAS [26, [86–88]. The consumption of heroin, basuco, non-prescription tranquilizers, stimulants, methamphetamines, opioids, and ketamine characterizes this region. This zone is the second most developed region in the country, and 72% of urban areas [60], [85](**S3 Table**, **Supplementary Material**).

Our findings also show that the Southern region is more likely to consume illegal drugs, including basuco, heroin, and Yagé (**Fig 8**). One of the main reasons for this result is that, unfortunately, this region has favourable environmental characteristics (i.e., majority rainforest) for their consumption and production, being the second largest illegal drug-producing region in Colombia [79]. Furthermore, this region has the highest percentage of rurality (52%) compared to the other regions, and its level of development is low as measured by the GDP [60, 85] (S3 **Table**, **Supplementary Material**).

In the Western region, also known as the Pacific region, consumption mostly mainly includes of Methylene Chloride, GHB, heroin, opioids, and methamphetamines. This region (Pacific) is mainly characterized known for its geographical isolation, poverty, and ongoing conflict, which have contributed to the growth of drug production and trafficking in the area. Poverty is one of the main factors driving drug production in the Pacific region, which has led many people to turn to drug cultivation and trafficking for survival. Additionally, the region's rugged terrain and limited infrastructure have made it difficult for the Colombian government to establish a strong presence, allowing drug traffickers to operate with relative impunity [89]. This region has a similar percentage of urban (53%) and rural (47%) populations than the Southern region, and ranks second among the regions with the lowest levels of development (S3 **Table**, **Supplementary Material**).

In the Western region, also known as the Pacific region, consumption mainly includes methylene chloride, GHB, heroin, opioids, and methamphetamines. This region is mainly characterized for its geographical isolation, poverty, and ongoing conflict, which have contributed to the growth of drug production and trafficking in the area. Poverty is one of the main factors driving drug production in the Pacific region, which has led many people to turn to drug cultivation and trafficking for survival. Additionally, the region's rugged terrain and limited infrastructure have made it difficult for the Colombian government to establish a strong presence, allowing drug traffickers to operate with relative impunity [89]. This region has a similar percentage of urban (53%) and rural (47%) populations than the Southern region, and ranks second among the regions with the lowest levels of development (S3 **Table**, **Supplementary Material**).[89]. This region has a similar percentage of urban (53%) and rural (47%) populations than the Southern region. On the other hand, this region ranks second among the regions with the lowest levels of development (S3 **Table**, **Supplementary Material**).

Conclusion

In summary, the proposed CAE-DEC model simultaneously integrates a feature extraction process within the clustering design, prioritizing features that improve the separation between groups, thus avoiding the manual extraction of features, which is a frequent process in traditional models. Additionally, a geospatial component is sequentially included to expand the resulting insights by considering geographic constraints. Currently, these types of architectures are scarce in understanding mental health problems. As part of future work, the architecture of the proposed model could be improved to integrate the automatic extraction of features while optimizing a geospatial loss. Following our experience with the proposed CAE-DEC in PAS consumption, the application of this model to other mental health problems, such as suicide, depression, and domestic violence, among other pathologies, could be explored. Based on these results, effective interventions and/or government policies to prevent and/or mitigate their impact could be promoted

and evaluated, for example, by developing regional interventions based on the types of drugs most prevalent in the area and the cultural and socio-economic characteristics. This can include education, treatment, and harm reduction programs. Also, this information can be used to develop public health campaigns to raise awareness about the risks of drug use and reduce their negative impact. Furthermore, this information can be used to crack down on drug trafficking and distribution networks. On the other hand, this information can be used to alert healthcare providers and regulatory bodies to take appropriate action to prevent their use and discover new drugs.

Acknowledgements. K.P. is a doctoral student at Universidad del Norte, Barranquilla, Colombia, and received a Ph.D. scholarship from this institution. Some of this work is to be presented to the Ph.D. program in partial fulfillment of the requirements for the Ph.D. degree. The funders had no role in study design, data collection and analysis, decision to publish, or preparation of the manuscript.

Authors' Contributors. All authors contributed to the design of the study. K.P. was responsible for data extraction and analysis; JIV and CB provided important insight and supervised the analysis. All authors reviewed the manuscript.

Declaration of Competing Interest. None to declare.

References

[1] OPS, “Abuso de sustancias ,” *Organización Panamericana de la Salud*, 2022. https://www.paho.org/es/temas/abuso-sustancias (accessed Jan. 25, 2022).

[2] C. Heesun, L. Jaesin, and K. Eunmi, “Trends of novel psychoactive substances (NPSs) and their fatal cases,” *Forensic Toxicol.*, vol. 34, no. 1, pp. 1–11, 2016, Accessed: Jan. 20, 2022. [Online]. Available: https://jglobal.jst.go.jp/en/detail?JGLOBAL_ID=201602283742633549

[3] A. L. Riley *et al.*, “Abuse potential and toxicity of the synthetic cathinones (i.e., ‘Bath salts’),” *Neurosci. Biobehav. Rev.*, vol. 110, pp. 150–173, Mar. 2020, doi: 10.1016/J.NEUBIOREV.2018.07.015.

[4] S. Assi, N. Gulyamova, P. Kneller, and D. Osselton, “The effects and toxicity of cathinones from the users’ perspectives: A qualitative study,” *Hum. Psychopharmacol. Clin. Exp.*, vol. 32, no. 3, p. e2610, May 2017, doi: 10.1002/HUP.2610.

[5] V. Lukić, R. Micić, B. Arsić, B. Nedović, and Ž. Radosavljević, “Overview of the major classes of new psychoactive substances, psychoactive effects, analytical determination and conformational analysis of selected illegal drugs,” *Open Chem.*, vol. 19, no. 1, pp. 60–106, Jan. 2021, doi: 10.1515/CHEM-2021-0196/XML.

[6] N. Uchiyama, S. Matsuda, M. Kawamura, R. Kikura-Hanajiri, and Y. Goda, “Two new-type cannabimimetic quinolinyl carboxylates, QUPIC and QUCHIC, two new cannabimimetic carboxamide derivatives, ADB-FUBINACA and ADBICA, and five synthetic cannabinoids detected with a thiophene derivative α-PVT and an opioid receptor agonist AH-7921 identified in illegal products,” *Forensic Toxicol. 2013 312*, vol. 31, no. 2, pp. 223–240, Mar. 2013, doi: 10.1007/S11419-013-0182-9.

[7] B. F. Grant *et al.*, “Prevalence and co-occurrence of substance use disorders and independent mood and anxiety disorders - Results from the national epidemiologic survey on alcohol and related conditions,” *Arch. Gen. Psychiatry*, vol. 61, no. 8, pp. 807–816, 2004, doi: 10.1001/archpsyc.61.8.807.

[8] CDC, “Understanding the Epidemic,” 2020. https://www.cdc.gov/opioids/basics/epidemic.html (accessed Jan. 21, 2022).

[9] R. Z. Goetzel, K. Hawkins, R. J. Ozminkowski, and S. H. Wang, “The health and productivity cost burden of the ‘top 10’ physical and mental health conditions affecting six large US employers in 1999,” *J. Occup. Environ. Med.*, vol. 45, no. 1, pp. 5–14, 2003, doi: 10.1097/00043764-200301000-00007.

[10] W. F. Stewart, J. A. Ricci, E. Chee, S. R. Hahn, and D. Morganstein, “Cost of lost productive work time among US workers with depression,” *JAMA-JOURNAL Am. Med. Assoc.*, vol. 289, no. 23, pp. 3135–3144, Jun. 2003, doi: 10.1001/jama.289.23.3135.

[11] F. L. G. Garcia and J. C. A. Murillo, “The United Nations and 21st century security challenges in Colombia,” *Rev. Cient. Gen. Jose Maria Cordova*, vol. 19, no. 36, pp. 929–940, 2021, doi: 10.21830/19006586.875.

[12] J. P. Aschner and J. C. Montero, “Architectures, spaces, and territories of illicit drug trafficking in Colombia and Mexico:,” *https://doi.org/10.1177/1741659020910212*, vol. 17, no. 3, pp. 327–351, Mar. 2020, doi: 10.1177/1741659020910212.

[13] ODC, “Observatorio de drogas de Colombia,” 2022. https://www.minjusticia.gov.co/programas-co/ODC/Paginas/SIDCO-departamento-municipio.aspx (accessed Jun. 09, 2022).

[14] DANE, “Encuesta Nacional de Consumo de Sustancias Psicoactivas,” 2020. Accessed: Apr. 23, 2021. [Online]. Available: https://www.dane.gov.co/files/investigaciones/boletines/encspa/comunicado-encspa-2019.pdf

[15] DANE, “Estudio nacional de consumo de sustancias psicoactivas en Colombia,” Bogotá, 2014. Accessed: Jan. 17, 2022. [Online]. Available: https://www.unodc.org/documents/colombia/2014/Julio/Estudio_de_Consumo_UNODC.pdf

[16] UNODC, “Drogas sintéticas y nuevas sustancias psicoactivas en América Latina y el Caribe

2021," Viena, 2021. Accessed: Jan. 21, 2022. [Online]. Available: https://www.minjusticia.gov.co/programas-co/ODC/Documents/Publicaciones/GlobalSmartLA (1).pdf?csf=1&e=MH9EHg

[17] P. Griffiths and R. Mcketin, "Developing a global perspective on drug consumption patterns and trends-the challenge for drug epidemiology," *Bull. Narcotics*, vol. 5, no. 1, 2003.

[18] W. A. Lanier, E. M. Johnson, R. T. Rolfs, M. D. Friedrichs, and T. C. Grey, "Risk factors for prescription opioid-related death, Utah, 2008-2009," *Pain Med.*, vol. 13, no. 12, pp. 1580–1589, 2012, doi: 10.1111/J.1526-4637.2012.01518.X.

[19] S. S. Martins, L. Sampson, M. Cerdá, and S. Galea, "Worldwide Prevalence and Trends in Unintentional Drug Overdose: A Systematic Review of the Literature," *Am. J. Public Health*, vol. 105, no. 11, pp. e29–e49, Nov. 2015, doi: 10.2105/AJPH.2015.302843.

[20] CDC, "Today's Heroin Epidemic ," *Centers for Disease Control and Prevention*, 2015. https://www.cdc.gov/vitalsigns/heroin/index.html (accessed Jan. 16, 2022).

[21] C. M. Fuller *et al.*, "Effects of race, neighborhood, and social network on age at initiation of injection drug use," *Am. J. Public Health*, vol. 95, no. 4, pp. 689–695, Apr. 2005, doi: 10.2105/AJPH.2003.02178.

[22] P. J. Fite, P. Wynn, J. E. Lochman, and K. C. Wells, "The Influence of Neighborhood Disadvantage and Perceived Disapproval on Early Substance Use Initiation," *Addict. Behav.*, vol. 34, no. 9, p. 769, Sep. 2009, doi: 10.1016/J.ADDBEH.2009.05.002.

[23] S. R. Friedman *et al.*, "Income inequality, drug-related arrests, and the health of people who inject drugs: Reflections on seventeen years of research," *Int. J. Drug Policy*, vol. 32, pp. 11–16, Jun. 2016, doi: 10.1016/J.DRUGPO.2016.03.003.

[24] M. Jensen, L. Chassin, and N. A. Gonzales, "Neighborhood Moderation of Sensation Seeking Effects on Adolescent Substance Use Initiation," *J. Youth Adolesc.*, vol. 46, no. 9, p. 1953, Sep. 2017, doi: 10.1007/S10964-017-0647-Y.

[25] C. Sarah and J. Leonard A, "Contextual Perspectives on Heroin Addiction and Recovery:

Classic and Contemporary Theories," *Int. Arch. Public Heal. Community Med.*, vol. 2, no. 1, Dec. 2018, doi: 10.23937/IAPHCM-2017/1710009.

[26] P. Bozorgi, D. E. Porter, J. M. Eberth, J. P. Eidson, and A. Karami, "The leading neighborhood-level predictors of drug overdose: A mixed machine learning and spatial approach," *Drug Alcohol Depend.*, vol. 229, p. 109143, Dec. 2021, doi: 10.1016/J.DRUGALCDEP.2021.109143.

[27] S. Galea, S. Rudenstine, and D. Vlahov, "Drug use, misuse, and the urban environment," *Drug Alcohol Rev.*, vol. 24, no. 2, pp. 127–136, Mar. 2005, doi: 10.1080/09595230500102509.

[28] C. A. Latkin, V. Forman, A. Knowlton, and S. Sherman, "Norms, social networks, and HIV-related risk behaviors among urban disadvantaged drug users," *Soc. Sci. Med.*, vol. 56, no. 3, pp. 465–476, Feb. 2003, doi: 10.1016/S0277-9536(02)00047-3.

[29] J. R. Schroeder, C. A. Latkin, D. R. Hoover, A. D. Curry, A. R. Knowlton, and D. D. Celentano, "Illicit drug use in one's social network and in one's neighborhood predicts individual heroin and cocaine use," *Ann. Epidemiol.*, vol. 11, no. 6, pp. 389–394, 2001, doi: 10.1016/S1047-2797(01)00225-3.

[30] A. Campo-Arias, Y. P. Suárez-Colorado, and C. C. Caballero- Domínguez, "Asociación entre el consumo de Cannabis y el riesgo de suicidio en adolescentes escolarizados de Santa Marta, Colombia," *Biomédica*, vol. 40, no. 3, p. 569, 2020, doi: 10.7705/BIOMEDICA.4988.

[31] A. L. Fajardo, "Consumption of psychopharmaceuticals in the city of Bogota (Colombia): a new reality," *Arch. Med.*, vol. 18, no. 2, 2018, doi: 10.30554/archmed.18.2.2743.2018.

[32] O. Scoppetta and G. A. Castaño, "Early drug consumption and subsequent risk of illicit drug use in Colombia," *Addict. Disord. their Treat.*, vol. 18, no. 1, pp. 10–14, Mar. 2019, doi: 10.1097/ADT.0000000000000144.

[33] C. Scheuer *et al.*, "El consumo de sustancias psicoactivas en jóvenes estudiantes de una institución educativa del municipio de Neira (Caldas): un estudio de caso desde la mirada de

la educación inclusiva," *Cult. y Drog.*, vol. 23, no. 26, pp. 343–354, Jul. 2018, doi: 10.2/JQUERY.MIN.JS.

[34] J. Kalyanam, T. Katsuki, G. R.G. Lanckriet, and T. K. Mackey, "Exploring trends of nonmedical use of prescription drugs and polydrug abuse in the Twittersphere using unsupervised machine learning," *Addict. Behav.*, vol. 65, pp. 289–295, Feb. 2017, doi: 10.1016/j.addbeh.2016.08.019.

[35] M. A. Narvaez-Chicaiza, "Harm Reduction Policies Where Drugs Constitute a Security Issue," *Heal. Care Anal.*, vol. 28, no. 4, pp. 382–390, Dec. 2020, doi: 10.1007/S10728-020-00415-9.

[36] S. M. Restrepo-Escobar and E. A. S. Cardona, "Educational and prevention campaigns. A review on the use of psychoactive substances in Colombian university students," *Interdisciplinaria*, vol. 38, no. 2, pp. 199–208, 2021, doi: 10.16888/INTERD.2021.38.2.13.

[37] E. Hancer, B. Xue, and M. Zhang, "A survey on feature selection approaches for clustering," *Artif. Intell. Rev. 2020 536*, vol. 53, no. 6, pp. 4519–4545, Jan. 2020, doi: 10.1007/S10462-019-09800-W.

[38] T. D. Wager, L. Y. Atlas, M. A. Lindquist, M. Roy, C.-W. Woo, and E. Kross, "An fMRI-Based Neurologic Signature of Physical Pain," *N. Engl. J. Med.*, vol. 368, no. 15, pp. 1388–1397, 2013, doi: 10.1056/NEJMoa1204471.

[39] A. Henriksson, M. Kvist, H. Dalianis, and M. Duneld, "Identifying adverse drug event information in clinical notes with distributional semantic representations of context," *J. Biomed. Inform.*, vol. 57, pp. 333–349, Oct. 2015, doi: 10.1016/j.jbi.2015.08.013.

[40] L. M. Squeglia *et al.*, "Neural Predictors of Initiating Alcohol Use During Adolescence," *Am. J. Psychiatry*, vol. 174, no. 2, pp. 172–185, Feb. 2017, doi: 10.1176/appi.ajp.2016.15121587.

[41] M. Conway and D. O'Connor, "Social media, big data, and mental health: current advances and ethical implications," *Curr. Opin. Psychol.*, vol. 9, pp. 77–82, Jun. 2016, doi: 10.1016/j.copsyc.2016.01.004.

[42] T. Katsuki, T. K. Mackey, and R. Cuomo, “Establishing a Link Between Prescription Drug Abuse and Illicit Online Pharmacies: Analysis of Twitter Data,” *J. Med. INTERNET Res.*, vol. 17, no. 12, 2015, doi: 10.2196/jmir.5144.

[43] L. Degenhardt *et al.*, “The global epidemiology and burden of psychostimulant dependence: Findings from the Global Burden of Disease Study 2010,” *Drug Alcohol Depend.*, vol. 137, pp. 36–47, 2014, doi: 10.1016/j.drugalcdep.2013.12.025.

[44] H. A. Whiteford *et al.*, “Global burden of disease attributable to mental and substance use disorders: findings from the Global Burden of Disease Study 2010,” *Lancet*, vol. 382, no. 9904, pp. 1575–1586, Nov. 2013, doi: 10.1016/S0140-6736(13)61611-6.

[45] F. D. Bowman, B. Caffo, S. S. Bassett, and C. Kilts, “A Bayesian hierarchical framework for spatial modeling of fMRI data,” *Neuroimage*, vol. 39, no. 1, pp. 146–156, 2008, doi: 10.1016/j.neuroimage.2007.08.012.

[46] K. Shannon, M. Rusch, J. Shoveller, D. Alexson, K. Gibson, and M. W. Tyndall, “Mapping violence and policing as an environmental-structural barrier to health service and syringe availability among substance-using women in street-level sex work,” *Int. J. DRUG POLICY*, vol. 19, no. 2, pp. 140–147, 2008, doi: 10.1016/j.drugpo.2007.11.024.

[47] B. Freisthler, B. Needell, and P. J. Gruenewald, “Is the physical availability of alcohol and illicit drugs related to neighborhood rates of child maltreatment?,” *Child Abuse Negl.*, vol. 29, no. 9, pp. 1049–1060, Sep. 2005, doi: 10.1016/j.chiabu.2004.12.014.

[48] J. K. Bass and S. F. Lambert, “Urban adolescents’ perceptions of their neighborhoods: An examination of spatial dependence,” *J. Community Psychol.*, vol. 32, no. 3, pp. 277–293, May 2004, doi: 10.1002/jcop.20005.

[49] B. Chaix *et al.*, “Spatial clustering of mental disorders and associated characteristics of the neighbourhood context in Malmo, Sweden, in 2001,” *J. Epidemiol. Community Health*, vol. 60, no. 5, pp. 427–435, May 2006, doi: 10.1136/jech.2005.040360.

[50] C. T. Mowbray, M. C. Holter, G. B. Teague, and D. Bybee, “Fidelity criteria: Development,

measurement, and validation," *Am. J. Eval.*, vol. 24, no. 3, pp. 315–340, 2003, doi: 10.1177/109821400302400303.

[51] M. Peet and C. Stokes, "Omega-3 fatty acids in the treatment of psychiatric disorders," *Drugs*, vol. 65, no. 8, pp. 1051–1059, 2005, doi: 10.2165/00003495-200565080-00002.

[52] K. Chichester *et al.*, "Pharmacies and features of the built environment associated with opioid overdose: A geospatial comparison of rural and urban regions in Alabama, USA," *Int. J. Drug Policy*, vol. 79, May 2020, doi: 10.1016/J.DRUGPO.2020.102736.

[53] P. Geissert *et al.*, "High-risk prescribing and opioid overdose: prospects for prescription drug monitoring program-based proactive alerts," *Pain*, vol. 159, no. 1, pp. 150–156, Jan. 2018, doi: 10.1097/J.PAIN.0000000000001078.

[54] C. Fraley and A. E. Raftery, "How many clusters? Which clustering method? Answers via model-based cluster analysis," *Comput. J.*, vol. 41, no. 8, pp. 586–588, 1998, doi: 10.1093/COMJNL/41.8.578.

[55] A. Saxena *et al.*, "A review of clustering techniques and developments," *Neurocomputing*, vol. 267, pp. 664–681, Dec. 2017, doi: 10.1016/J.NEUCOM.2017.06.053.

[56] B. Li, D. Pi, Y. Lin, and L. Cui, "DNC: A Deep Neural Network-based Clustering-oriented Network Embedding Algorithm," *J. Netw. Comput. Appl.*, vol. 173, Jan. 2021, doi: 10.1016/J.JNCA.2020.102854.

[57] J. Xie, R. Girshick, and A. Farhadi, "Unsupervised Deep Embedding for Clustering Analysis," *33rd Int. Conf. Mach. Learn. ICML 2016*, vol. 1, pp. 740–749, Nov. 2016, Accessed: Jan. 15, 2022. [Online]. Available: https://arxiv.org/abs/1511.06335v2

[58] S. Sharifipour, H. Fayyazi, and M. Sabokro, "Unsupervised Feature Selection using Encoder-Decoder Networks," *6th Iran. Conf. Signal Process. Intell. Syst. ICSPIS 2020*, Dec. 2020, doi: 10.1109/ICSPIS51611.2020.9349608.

[59] B. Li, D. Pi, Y. Lin, and L. Cui, "DNC: A Deep Neural Network-based Clustering-oriented Network Embedding Algorithm," *J. Netw. Comput. Appl.*, vol. 173, no. May 2020, p.

102854, 2021, doi: 10.1016/j.jnca.2020.102854.

[60] DANE, “Departamento Administrativo Nacional de Estadística. Censo Nacional de Población y Vivienda 2018. Proyecciones de Población 2018-2020, total municipal por área Junio 30.” Bogotá D.C, Colombia, 2018.

[61] DNP, “Avances y complementariedades estratégicas de los Distritos en el marco de los esquemas asociativos territoriales,” Bogotá D.C, 2018. [Online]. Available: https://colaboracion.dnp.gov.co/CDT/Desarrollo Territorial/Conversatorio Distrito Cali 04_10_2018 - Santiago Arroyo.pdf

[62] UNODC, “Monitoreo de territorios afectados por cultivos ilícitos 2020,” Bogotá, 2021. Accessed: Jan. 14, 2022. [Online]. Available: https://www.unodc.org/documents/crop-monitoring/Colombia/Colombia_Monitoreo_de_territorios_afectados_por_cultivos_ilicitos_2020.pdf

[63] ODC, “Estudio nacional de consumo de sustancias psicoactivas,” Bogotá, 2019. Accessed: Jan. 14, 2022. [Online]. Available: https://www.odc.gov.co/Portals/1/publicaciones/pdf/estudio Nacional de consumo 2019.pdf

[64] DANE, “Encuesta Nacional de Consumo de Sustancias Psicoactivas en Población General 2019,” *2020*. https://microdatos.dane.gov.co/index.php/catalog/680/get_microdata (accessed Jan. 14, 2022).

[65] J. Espinosa, “Shapefile,” 2022. https://hub.arcgis.com/datasets/de0e829ddbf743c895ba6dcee1b74fae/about (accessed Jun. 09, 2022).

[66] L. Anselin, *Spatial Econometrics: Methods and Models*, Springer Netherlands. 1988. doi: 10.1007/978-94-015-7799-1_1.

[67] P. Moran, *The Interpretation of Statistical Maps* , 2nd ed., vol. 10. Journal of the Royal Statistical Society, 1948. Accessed: Jan. 23, 2022. [Online]. Available: https://www.jstor.org/stable/2983777

[68] R. C. Geary, “The Contiguity Ratio and Statistical Mapping,” *Inc. Stat.*, vol. 5, no. 3, p. 115, Nov. 1954, doi: 10.2307/2986645.

[69] A. Getis and J. K. Ord, “The Analysis of Spatial Association by Use of Distance Statistics,” *Geogr. Anal.*, vol. 24, no. 3, pp. 189–206, Jul. 1992, doi: 10.1111/J.1538-4632.1992.TB00261.X.

[70] L. Anselin, “Local Indicators of Spatial Association—LISA,” *Geogr. Anal.*, vol. 27, no. 2, pp. 93–115, Apr. 1995, doi: 10.1111/J.1538-4632.1995.TB00338.X.

[71] J. C. Duque, R. Ramos, and J. Suriñach, “Supervised Regionalization Methods: A Survey:,” *http://dx.doi.org/10.1177/0160017607301605*, vol. 30, no. 3, pp. 195–220, Jul. 2016, doi: 10.1177/0160017607301605.

[72] S. Rey, D. Arribas-Bel, and L. Wolf, *Geographic Data Science with Python*. 2020. Accessed: Jan. 23, 2022. [Online]. Available: https://geographicdata.science/book/intro.html

[73] G. Van Rossum and F. L. Drake, *Python 3 Reference Manual*. Scotts Valley, CA: CreateSpace, 2009.

[74] J. Masci, U. Meier, D. Cireşan, and J. Schmidhuber, “Stacked Convolutional Auto-Encoders for Hierarchical Feature Extraction,” *Lect. Notes Comput. Sci. (including Subser. Lect. Notes Artif. Intell. Lect. Notes Bioinformatics)*, vol. 6791 LNCS, no. PART 1, pp. 52–59, 2011, doi: 10.1007/978-3-642-21735-7_7.

[75] A. Shrestha and A. Mahmood, “Review of deep learning algorithms and architectures,” *IEEE Access*, vol. 7, pp. 53040–53065, 2019, doi: 10.1109/ACCESS.2019.2912200.

[76] T. Caliñski and J. Harabasz, “A Dendrite Method Foe Cluster Analysis,” *Commun. Stat.*, vol. 3, no. 1, pp. 1–27, 1974, doi: 10.1080/03610927408827101.

[77] D. L. Davies and D. W. Bouldin, “A Cluster Separation Measure,” *IEEE Trans. Pattern Anal. Mach. Intell.*, vol. PAMI-1, no. 2, pp. 224–227, 1979, doi: 10.1109/TPAMI.1979.4766909.

[78] P. J. Rousseeuw, “Silhouettes: A graphical aid to the interpretation and validation of cluster

analysis,” *J. Comput. Appl. Math.*, vol. 20, no. C, pp. 53–65, Nov. 1987, doi: 10.1016/0377-0427(87)90125-7.

[79] ODC, “Density of drug production in Colombia,” 2021. https://www.datos.gov.co/d/acs4-3wgp/visualization (accessed Jul. 05, 2022).

[80] H. Clarke, N. Soneji, D. T. Ko, L. Yun, and D. N. Wijeysundera, “Rates and risk factors for prolonged opioid use after major surgery: population based cohort study,” *BMJ*, vol. 348, Feb. 2014, doi: 10.1136/BMJ.G1251.

[81] Y. F. Kuo, M. A. Raji, N. W. Chen, H. Hasan, and J. S. Goodwin, “Trends in Opioid Prescriptions Among Part D Medicare Recipients From 2007 to 2012,” *Am. J. Med.*, vol. 129, no. 2, pp. 221.e21-221.e30, Feb. 2016, doi: 10.1016/J.AMJMED.2015.10.002.

[82] S. Puigcorbé *et al.*, “Assessing the association between tourism and the alcohol urban environment in Barcelona: a cross-sectional study,” *BMJ Open*, vol. 10, no. 9, p. e037569, Sep. 2020, doi: 10.1136/BMJOPEN-2020-037569.

[83] M. Easwaran, J. Bazroy, V. Jayaseelan, and Z. Singh, “Prevalence and determinants of alcohol consumption among adult men in a coastal area of south India,” *Int. J. Med. Sci. Public Heal.*, vol. 4, no. 3, p. 360, 2015, doi: 10.5455/IJMSPH.2015.1010201479.

[84] P. Chinnakali, P. Thekkur, A. Manoj Kumar, G. Ramaswamy, B. Bharadwaj, and G. Roy, “Alarmingly high level of alcohol use among fishermen: A community based survey from a coastal area of south India,” *J. Forensic Leg. Med.*, vol. 42, pp. 41–44, Aug. 2016, doi: 10.1016/J.JFLM.2016.05.006.

[85] DANE, “Producto Interno Bruto por departamento,” 2021.

[86] M. C. García *et al.*, “Opioid Prescribing Rates in Nonmetropolitan and Metropolitan Counties Among Primary Care Providers Using an Electronic Health Record System — United States, 2014–2017,” *MMWR. Morb. Mortal. Wkly. Rep.*, vol. 68, no. 2, pp. 25–30, Jan. 2019, doi: 10.15585/MMWR.MM6802A1.

[87] K. M. Keyes, M. Cerdá, J. E. Brady, J. R. Havens, and S. Galea, “Understanding the Rural–

Urban Differences in Nonmedical Prescription Opioid Use and Abuse in the United States," *Am. J. Public Health*, vol. 104, no. 2, p. e52, Feb. 2014, doi: 10.2105/AJPH.2013.301709.

[88] N. B. King, V. Fraser, C. Boikos, R. Richardson, and S. Harper, "Determinants of Increased Opioid-Related Mortality in the United States and Canada, 1990–2013: A Systematic Review," *Am. J. Public Health*, vol. 104, no. 8, p. e32, 2014, doi: 10.2105/AJPH.2014.301966.

[89] UNODC, "Persistencia de los cultivos de coca en la Región Pacífica," 2010.

Supporting information

S1 Table. Feature coherence measurements

S2 Table. Model comparison

S3 Table. Characteristics of the level of development, urbanity, rurality, and drug production in the regions of Colombia.

S1 Fig. The optimal number of clusters using Dendrogram.

S2 Fig. Maps of Local Indicators of Spatial Association (LISA). Republished from [65] under a CC BY license, with permission from [ArcGIS Hub], original copyright [2016].

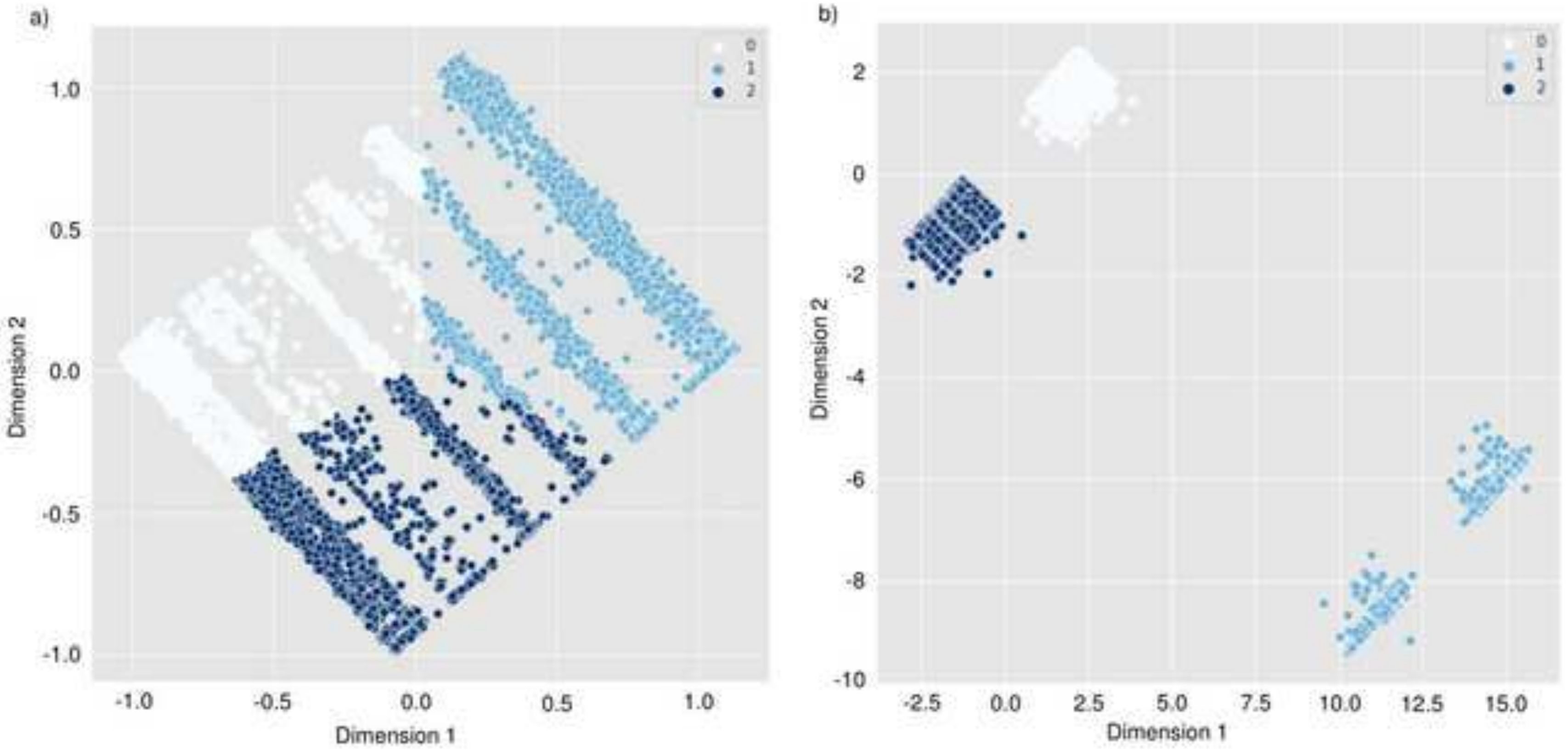
a)
0
1
2
1.0
0.5
0.0
-0.5
-1.0
Dimension 2
-1.0
-0.5
0.0
0.5
1.0
Dimension 1
b)
0
1
2
2
0
-2
-4
-6
-8
-10
Dimension 2
-2.5
0.0
2.5
5.0
7.5
10.0
12.5
15.0
Dimension 1

Figure

Click here to access/download;Figure;Fig3.tif

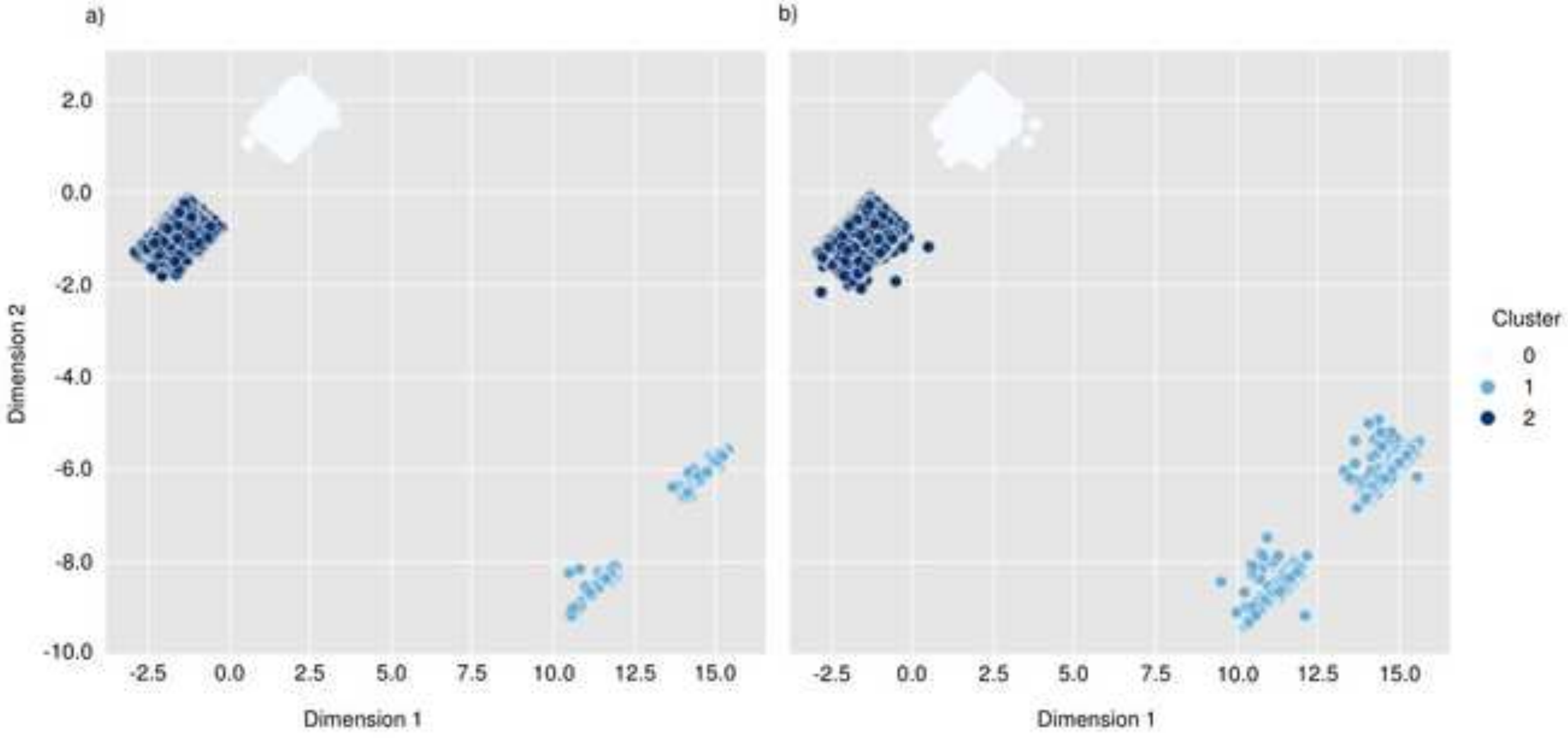

Figure

Click here to access/download;Figure;Fig4.tif

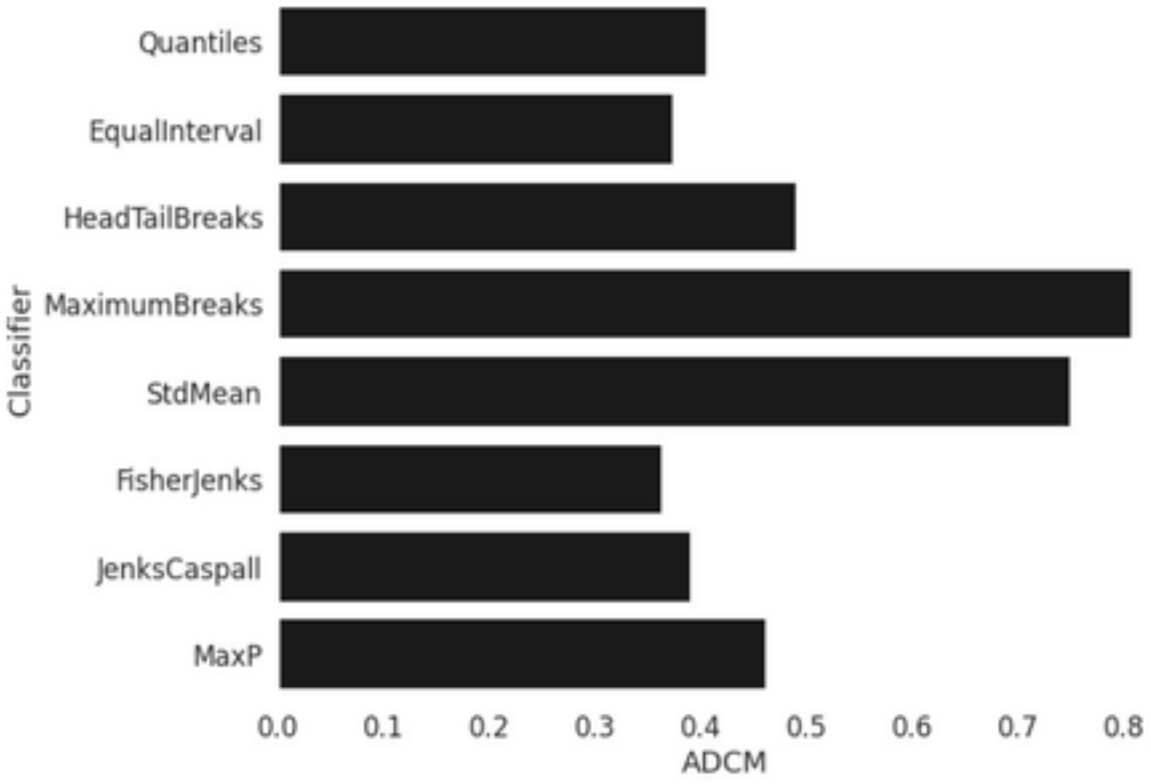

Figure Click here to access/download;Figure;Fig5.tif

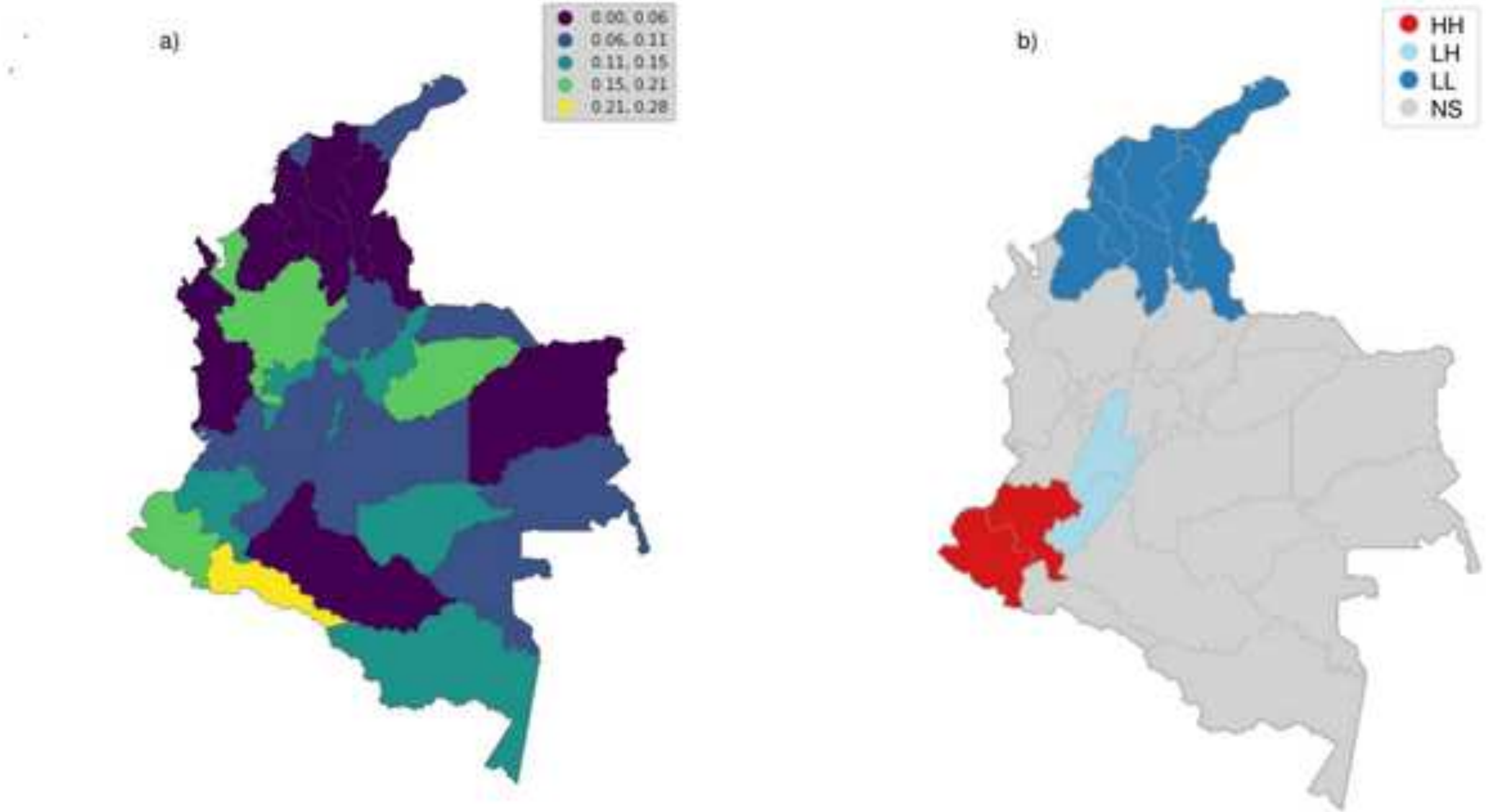

Figure

Click here to access/download;Figure;Fig6.tif

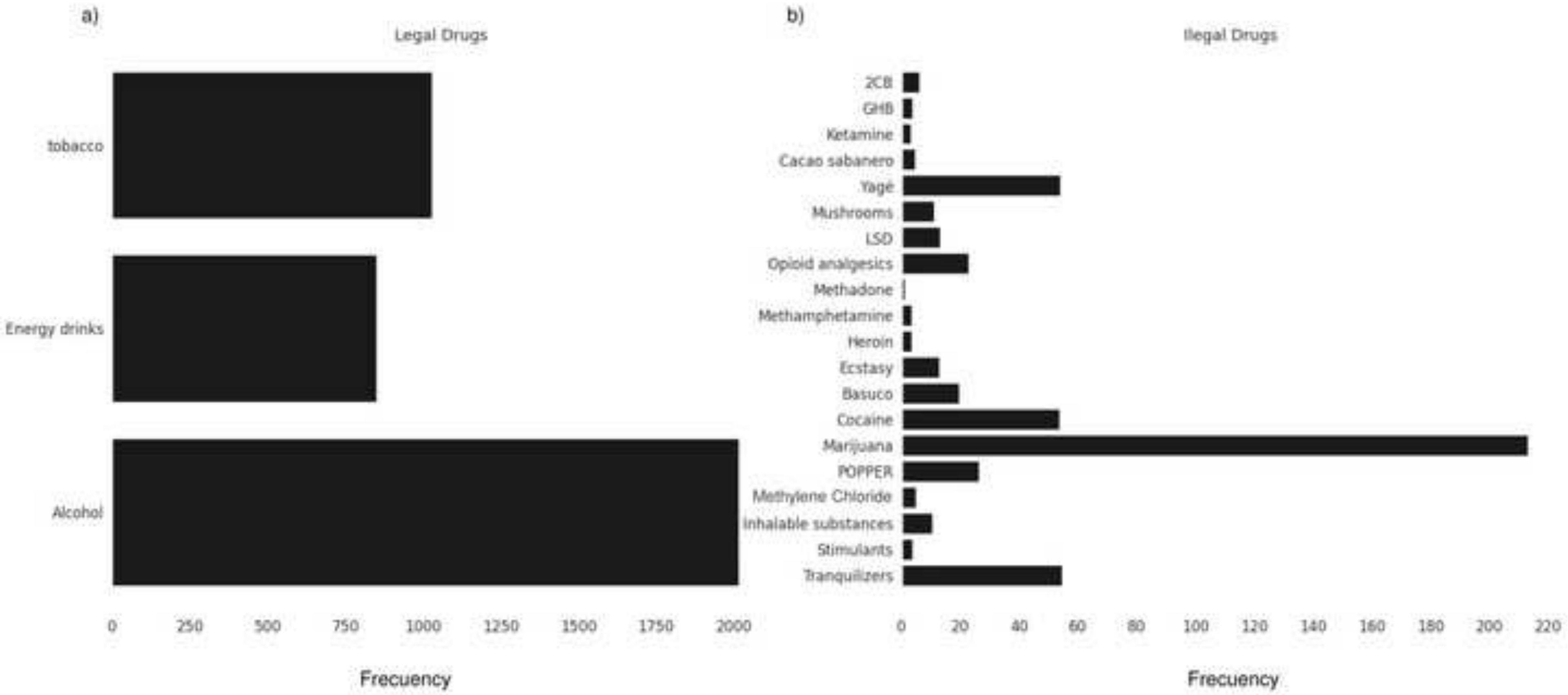

Figure

Click here to access/download;Figure;Fig7.tif

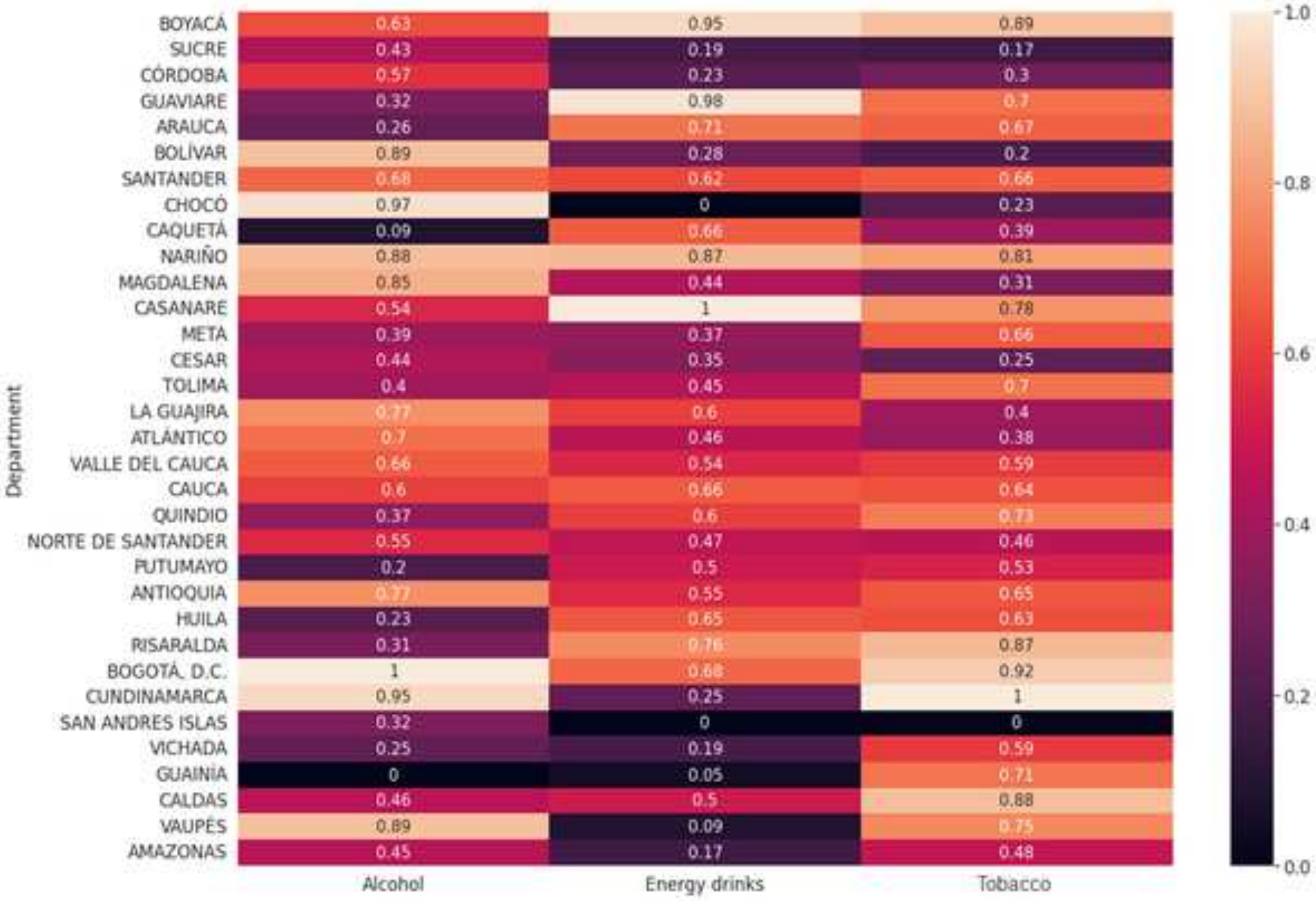

Click here to access/download;Figure;Fig8.tif

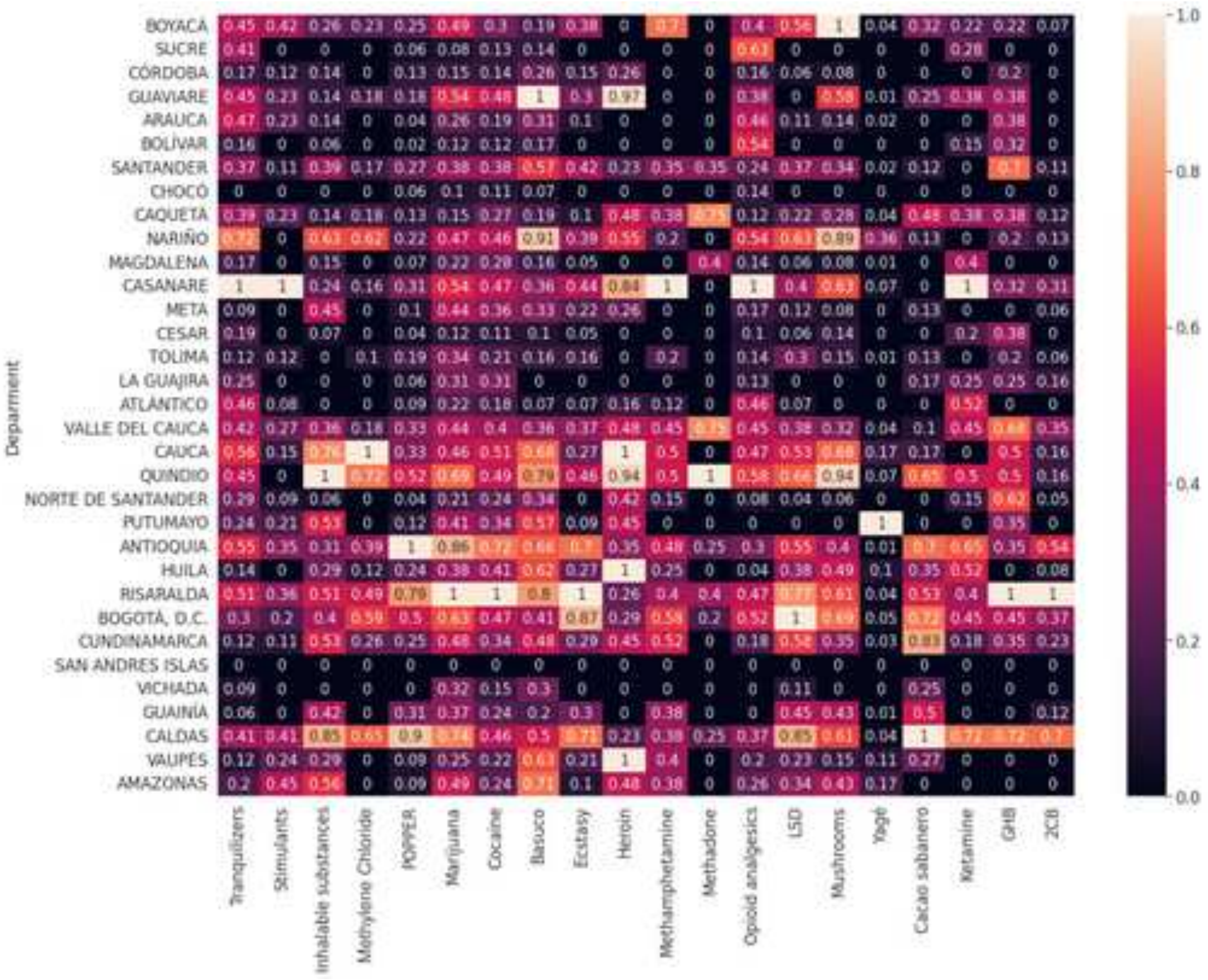

Figure

Click here to access/download;Figure;Fig9.tif

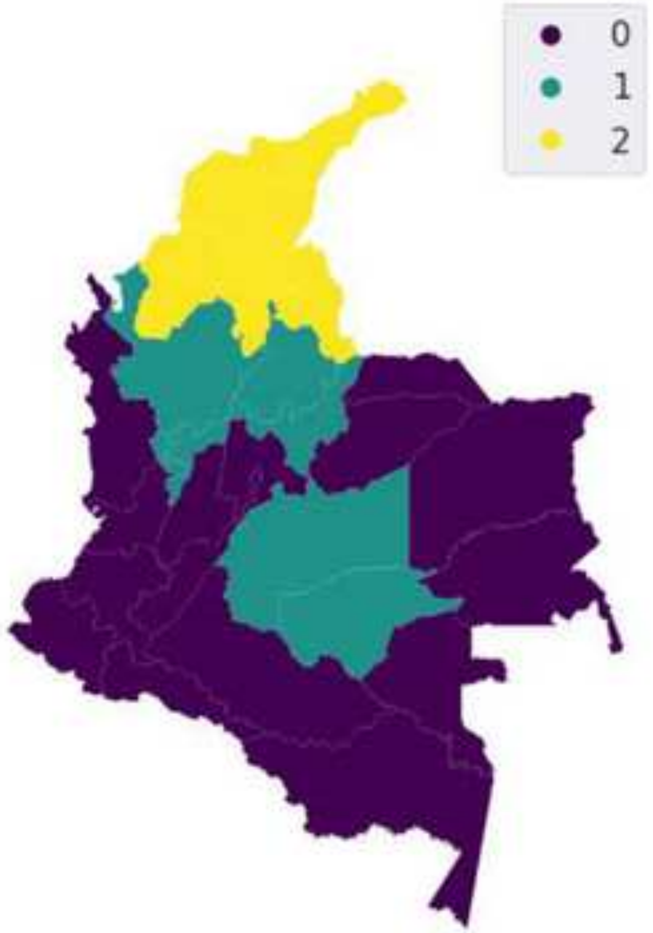

Figure

Click here to access/download;Figure;Fig1.tif

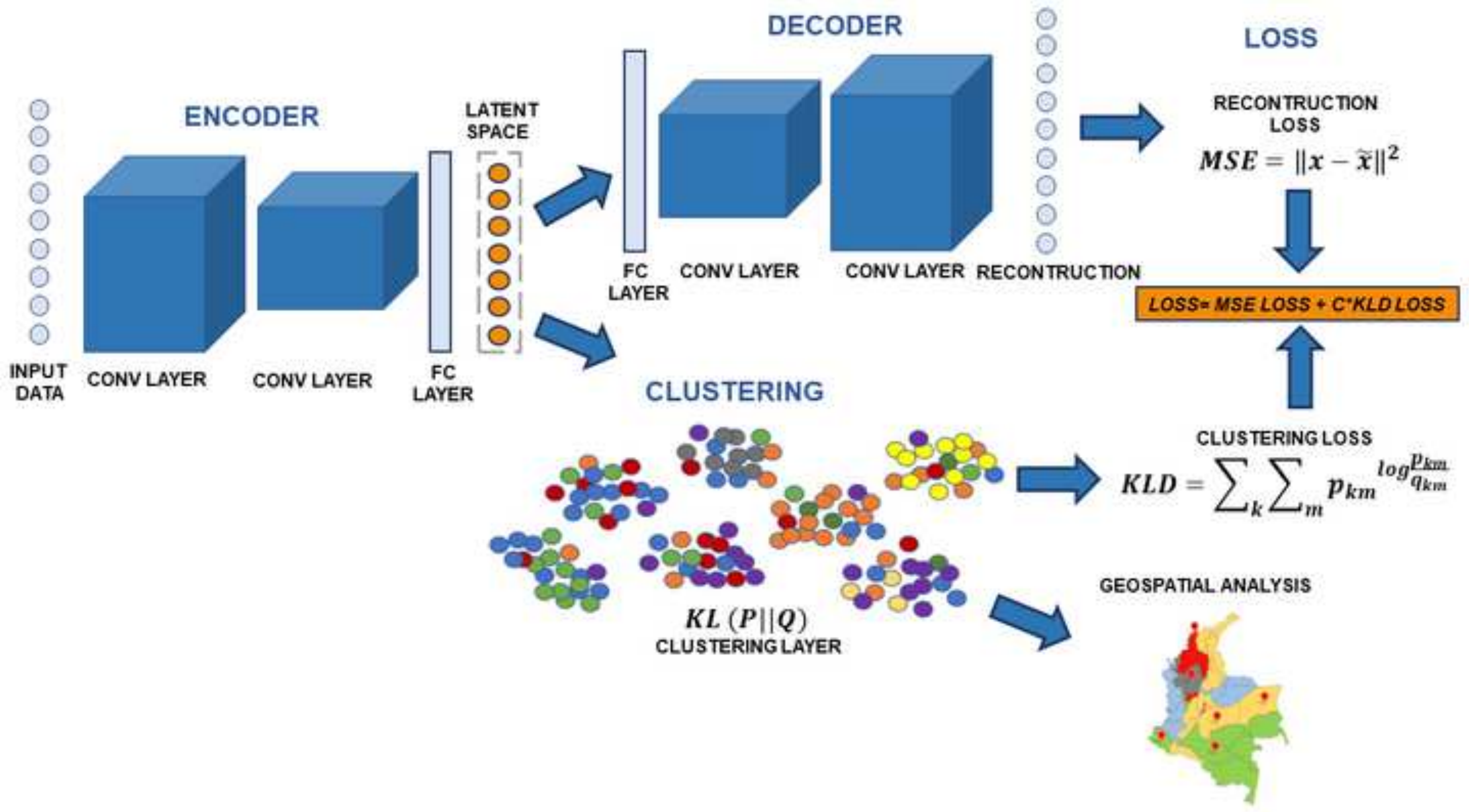

Click here to access/download
Supporting Information
S1_Fig.docx

Click here to access/download
Supporting Information
S1_Table.docx

Click here to access/download
Supporting Information
S2_Table.docx

Click here to access/download
Supporting Information
S3_Table.docx

Click here to access/download
Supporting Information
S2_Fig.docx

Leading ~~consumption~~ Consumption ~~patterns~~ Patterns of ~~psychoactive~~ Psychoactive S~~s~~ubstances in ~~c~~Colombia: ~~a~~ A ~~deep~~ Deep ~~neural~~ Neural ~~network~~Network-based ~~clustering~~Clustering-oriented ~~embedding~~ Embedding ~~approach~~ Approach

Kevin Palomino[1,*#], Carmen R. Berdugo[1], Jorge I. Vélez[1]

~~September FebruaryMarch 22381, 20232~~

[1] ~~U~~*Department of Industrial Engineering, Universidad del Norte, Barranquilla, Colombia.*

[#*] ~~Correspondence to be directed to:~~Corresponding author.

~~Mr. Kevin Palomino,~~
~~Department of Industrial Engineering,~~
~~Universidad del Norte,~~
~~Km 5 via Puerto Colombia,~~
~~Barranquilla, Colombia~~
E-mail: krpalomino@uninorte.edu.co ~~(KP)~~

ORCIDs
KP: https://orcid.org/0000-0002-2708-1096
~~JIV: https://orcid.org/0000-0002-3146-7899~~
CB: https://orcid.org/0000-0002-4803-2816
JIV: https://orcid.org/0000-0002-3146-7899

~~**Running Title:** Leading Consumption Patterns of Psychoactive Substances in Colombia~~
~~**Number of Characters:** 136 135 (title), 67 (running title)~~
~~**Number of Words:** 236 290 (abstract), 5888 5971 (main text)~~
~~**Number of Keywords:** 6~~
~~**Number of Figures:** 9~~
~~**Number of Tables:** 2~~
~~**Supplementary Material:** Figures: Fig_S1.docx, Fig_S2.docx,~~
~~Tables: Table_S1.docx, Table_S2.docx, Table_S3.docx~~

Formatted: English (Australia)
Formatted: English (Australia)
Formatted: English (Australia)
Formatted: English (United States)
Formatted: English (United States)
Formatted: English (Australia)
Formatted: Indent: First line: 0"
Formatted: Underline
Formatted: Font: Not Bold
Formatted: Font: Not Bold
Formatted: Font: Not Bold
Formatted: Font: Not Bold

~~ABSTRACT~~Abstract

The number of health-related incidents caused using illegal and legal psychoactive substances (PAS) has dramatically increased over two decades worldwide. In Colombia, the use of illicit substances has increased up to 10.3%, while the consumption alcohol and tobacco has increased to 84% and 12%, respectively. It is well-known that identifying drug consumption patterns in the general population is essential in reducing overall drug consumption. However, existing approaches do not incorporate Machine Learning and/or Deep Data Mining methods in combination with spatial techniques. To enhance our understanding of mental health issues related to PAS and assist in the development of national policies, here we present a novel Deep Neural Network-based Clustering-oriented Embedding Algorithm that incorporates an autoencoder and spatial techniques. The primary goal of our model is to identify general and spatial patterns of drug consumption and abuse, while also extracting relevant features from the input data and identifying clusters during the learning process. As a test case, we used the largest publicly available database of legal and illegal PAS consumption comprising 49,600 Colombian households. We estimated and geographically represented the prevalence of consumption and/or abuse of both PAS and non-PAS, while achieving statistically significant goodness-of-fit values. Our results indicate that region, sex, housing type, socioeconomic status, age, and variables related to household finances contribute to explaining the patterns of consumption and/or abuse of PAS. Additionally, we identified three distinct patterns of PAS consumption and/or abuse. At the spatial level, these patterns indicate concentrations of drug consumption in specific regions of the country, which are closely related to specific geographic locations and the prevailing social and environmental contexts. These findings can provide valuable insights to facilitate decision-making and develop national policies targeting specific groups given their cultural, geographic, and social conditions.

~~To contribute to the understanding of the mental health problem caused by psychoactive substances and facilitate decision-making and national policy development processes, here we propose a deep clustering model integrating an autoencoder and spatial techniques to identify general and spatial~~

Formatted: Font: 18 pt

Formatted: Font color: Text 1

Formatted: Font color: Text 1

Formatted: Font color: Text 1

Formatted: Font color: Text 1, Not Highlight

Formatted: Font color: Text 1

Formatted: Font: (Default) Times New Roman

Formatted: Font: (Default) Times New Roman

Formatted: Font: (Default) Times New Roman, English (Australia)

~~patterns of consumption and/or abuse of drugs in Colombia. This model is capable of extracting features from the input data and identifying clusters during the learning process. As a test case, we used the largest publicly available database comprising 49,600 households. Using this approach, we estimated and geographically represented the prevalence of consumption and/or abuse of both substances and non-psychoactive substances in Colombia, while achieving statistically significant goodness-of-fit values. Our results indicate that variables such as region, sex, housing type, socioeconomic status, age, and variables related to household finances, statistically significantly contribute to explain the consumption and/or abuse patterns of psychoactive substances. We also identified three differential consumption and/or abuse patterns of psychoactive substances in the Colombian territory. These patters, at the spatial level, highlight concentrations of drug consumption in particular regions of the country, and associated with specific geographic locations as well as the inherent social and environmental contexts.~~

***Keywords*:** Autoencoder, Machine Learning, Spatial analysis, ~~Clustering,~~ Drug consumption/abuse~~,~~ ~~Psychoactive substances.~~.

Formatted: Font color: Text 1

Formatted: Font: (Default) Times New Roman

Formatted: Font: (Default) Times New Roman

Formatted: Font: (Default) Times New Roman, English (Australia)

Author summary

Why was this study done?

- Mental disorders caused by psychoactive substances (PAS) have a high global disease burden and result in significant costs to society.
- Interventions for PAS consumers currently follow international protocols and do not consider the individual or regional characteristics.
- Targeted interventions for specific groups based on their cultural, geographic, and social conditions are needed.~~Mental disorders caused by psychoactive substances are among the highest global disease burdens.~~
- ~~SPA consumers constitute a high cost to society due to loss of productivity, premature mortality, increased healthcare spending, and criminal justice and social welfare costs.~~
- ~~Consumer interventions are focused on international protocols and not on the specific characteristics of the region or the individual.~~
- ~~It is necessary to design interventions targeted to specific groups given their cultural, geographic, and social conditions.~~

What did the researchers do and find?

- We developed a novel georeferenced mathematical model to identify PAS consumption patterns, including spatial analysis to emphasize the need for georeferenced interventions.
- Our model identified and characterized three groups of PAS consumers based on location and sociodemographic information.~~We developed a mathematical model that identifies psychoactive substance consumption patterns using georeferenced sociodemographic data.~~
- ~~The model includes a spatial analysis to explain why it is essential to design interventions georeferenced.~~
- ~~Using the model proposed, we were able to find three groups of SPA consumers by their sociodemographic information and location.~~

Formatted: Font: 18 pt, Bold, Underline

Formatted: Font: 18 pt, Underline

Formatted: Font: 16 pt

Formatted: English (Australia)

Formatted: English (Australia)

Formatted: List Paragraph, Bulleted + Level: 1 + Aligned at: 0.25" + Indent at: 0.5"

Formatted: Font: (Default) Times New Roman, Not Bold

Formatted: Font: Not Bold

Formatted: Font: (Default) Times New Roman, Not Bold

Formatted: Font: 16 pt

Formatted: Font: Bold, English (Australia)

Formatted: List Paragraph, Bulleted + Level: 1 + Aligned at: 0.25" + Indent at: 0.5"

Formatted: Font: (Default) Times New Roman

Formatted: Font: Bold

Formatted: Font: (Default) Times New Roman

Formatted: Font: (Default) Times New Roman

Formatted: Font: (Default) Times New Roman

Formatted: Font: (Default) Times New Roman, English (Australia)

What do these findings mean?

- Design~~ing~~ and focus interventions on identified clusters to reduce negative impact of PSA drug consumption in specific areas.~~focusing on these clusters would reduce the negative impact of having high drug consumption in a given area.~~
- ~~Similar~~Future modelling studies could explore new ~~-modelling studies could be carried out to investigate new p~~patterns related to medical information.
- Policies for treating patients with high PAS consumption should consider the identified patterns in each region~~In the future, there could be a policy for treating patients with high drug consumption. The design of this policy should have as an input the patterns found in each region~~.

Formatted: Font: 16 pt

Formatted: Font: (Default) Times New Roman, Not Bold

Formatted: Font: (Default) Times New Roman, Not Bold

Formatted: Font: (Default) Times New Roman, Not Bold

Formatted: Font: (Default) Times New Roman, Not Bold

Formatted: List Paragraph, Bulleted + Level: 1 + Aligned at: 0.25" + Indent at: 0.5"

Formatted: Font: (Default) Times New Roman

Formatted: Font: (Default) Times New Roman

Formatted: Font: (Default) Times New Roman, English (Australia)

1. ~~INTRODUCTION~~Introduction

Psychoactive substances (PAS) are chemical substances that change the function of the nervous system and cause alterations in people's ~~the~~ perception, mood, consciousness, cognition, or behavior ~~of people~~ [1]. PAS can be grouped according to their chemical structure as synthetic cannabinoids, synthetic cathinones, phenethylamines, arylcyclohexylamines, tryptamines, indo~~-~~lalkylamines, new synthetic opioids, piperazines, ketamine, and designer benzodiazepines~~;~~. They can also be grouped ~~and~~ according to their origin as ~~of~~ a natural origin or synthetic molecules [2~~]–[~~4].

The increased numbers of drug use among young people ~~is~~ are drawing the attention of national governments [5]. Because the number of health-related incidents caused by using ~~illegal~~ legal and illegal ~~psychoactive~~ PAS ~~substances~~ worldwide has dramatically increased over the last two decades [2~~]~~, ~~[~~6], this ~~phenomena~~ phenomenon has become some of the largest burdens of disease [7~~]~~, ~~[~~8]. Drug use ~~constitute~~ constitutes a ~~major~~ high cost to society due to premature mortality, increased health expenditure, criminal justice (drug and micro-trafficking), social welfare costs, and other social consequences [9~~]~~, ~~[~~10].

Colombia is ranked as one of the largest drug producers in the world [11]. Unfortunately, the production and commercialization of drugs through drug~~-trafficking~~ and micro-trafficking, ~~that~~ constantly expands in locations with high levels of poverty and ~~a~~ limited government presence ~~of the government~~ [12]. Statistics and indicators of drug consumption, production, and distribution, as well as reports from the National Statistical System (DANE) of Colombia, highlight a dramatic increase in (1) drug production [13]; (2) intern consumption of ~~psychoactive substances~~PAS at early ages; and (3) the prevalence in ~~the~~ use and/or abuse of drugs have dramatically increased over the last 20 years [14~~]~~, ~~[~~15]. Furthermore, the country also has the highest prevalence of drug use among school students in recent years ~~when~~ compared to other Latin American countries [16]. Thus, there is an urgent need to develop effective interventions to prevent the use and/or abuse of ~~psychoactive substances~~PAS.

Formatted: Font: 18 pt

Formatted: Font color: Text 1

Formatted: Font color: Text 1

Formatted: Font color: Text 1

Formatted: Font color: Text 1

Formatted: Font color: Text 1

Formatted: Font color: Text 1

Formatted: Font color: Text 1

Formatted: Font color: Text 1

Formatted: Font color: Text 1

Formatted: Font color: Text 1

Formatted: Font color: Text 1

Formatted: Font: (Default) Times New Roman

Formatted: Font: (Default) Times New Roman

Formatted: Font: (Default) Times New Roman, English (Australia)

The first step towards reducing this consumption is to identify drug consumption patterns in the general population [17]. ~~Numerous~~ Several studies have identified patterns associated with drug use and consumption [18~~]~~, ~~[~~19]. According to the Center for Disease Control and Prevention, ~~people~~ individuals who do not have their own homes and ~~live in rented~~ ~~instead~~ live in rented accommodations are more likely to use drugs [20]. Other research studies suggest that neighborhood contextual characteristics may increase the risk of substance abuse [21~~]~~–~~[~~26]. Additionally, ~~and that~~ population density may also influence substance use and overdose risk through a higher level of socialization in densely populated urban areas [27~~]~~–~~[~~29]. Other authors have identified that ~~that~~ anxiety, sleep disorders, suicide, depression, and other mental illnesses ~~constitute~~ are risk factors for the consumption and abuse of ~~psychoactive substances~~PAS [30~~]~~, ~~[~~31]. ~~In addition~~Furthermore, ~~the~~ early marijuana use has been shown to ~~use of marijuana~~ increase~~s~~ the risk of consuming other ~~psychoactive substances~~ PAS ~~[32],~~ [32]. Furthermore, ~~and~~ people involved in sports and artistic activities perceive ~~consider~~ drugs ~~as an~~as enhancers element for ~~developing~~ improving their~~such~~ performance ~~activities~~ [33].

In Colombia, drug consumption patterns and risk factors have also been identified. For instance~~Indeed~~, Kalyanam et al. ~~Kalyanam *et al.* (2007)~~[34] analyzed ~~-~~the social impact of bas~~z~~uco and inhalant use among street youths~~;~~. Narvaez-Chicaiza ~~(2020)~~ ~~Narvaez-Chicaiza (2020)~~ [35] ~~assesses~~ assessed the social factors that lead~~ing~~ to the adoption of harm reduction policies and how ~~such~~ these factors influence treatments for substance abuse disorders~~,~~. Additionally, ~~and~~ Restrepo-Escobar & Cardona ~~(2021)~~~~Restrepo-Escobar & Cardona (2021)~~[36] ~~show~~ demonstrated that ~~that~~ university students with low satisfaction in their studies ~~are~~ tend to be heavy users of alcohol, tobacco, and marijuana. However, these approaches do not consider ~~the use of~~ ~~using~~the use of Machine Learning (ML) and/or Deep Data Mining techniques in combination with spatial models to analyze drug consumption data from the general population. ~~In this sense, t~~To ~~the best of~~ our knowledge, ~~we found~~ we have not found any robust ~~no robust~~ models integrating ML and spatial

models to identify drug consumption patterns using publicly available Colombian databases~~Colombian publicly available databases~~.

Although several techniques for analyzing drug consumption patterns are currently available (i.e., ML, Bayesian, spatial, traditional multivariate, or univariate statistical models, or, in some cases, a combination of these), new trends in pattern identification and analysis techniques focus on hybrid and ensemble models [37]. Currently, the most widely used ML techniques are Support Vector Machines (SVMs), Random Forest (RF), and Natural Language Processing (NLP)[38]–[42]. Among Bayesian models, Bayesian meta-regression (DisMod-MR), Bayesian hierarchical models, and Markov Chain Monte Carlo are the most attractive methods [43]–[45]. Regarding spatial models, Spatial Distributions, Spatial Regression Models, Spatial Scan Statistics, Variograms, and Social Mapping are the most ~~used~~ frequently used techniques [46]–[49]. ~~In terms of traditional statistical~~On the other hand, ~~models,~~ logistic regression, confirmatory factor analysis, and correlational analysis are the most employed traditional statistical ~~techniques~~ models to ~~disclose~~ identify drug-associated patterns [50]–[53].

Fraley and Raftery ~~in (1998)~~ [54] suggest separating clustering approaches into ~~two groups:~~ hierarchical and partitioning techniques ~~[54]~~. Partitioning techniques are divided into density-, model-, and grid-based methods, the most popular of which are *K*-means, PAM, CLARA~~, CLARA, NSM, FCM, EM,~~ , DBSCAN~~,~~ and CLIQUE. ~~Hierarchical techniques,~~ O~~o~~n the other hand, hierarchical techniques are divided into agglomerative and divisive methods.~~; o~~ Of these, the best-known methods are BRICH, CURE, ROCK, and~~,~~ CHAMELEON (see ~~Saxena et al. en el (2017)~~[55] for further reading). Although ~~all~~ these techniques have been shown to ~~have~~ perform well ~~a good fit if not~~ when relevant features are removed *a priori*, it is well~~-~~known that~~,~~ in clustering algorithms, irrelevant and redundant features in the data may degrade the quality of clusters~~, which~~ and lead~~s~~ to high computational cost. Therefore, removing such features may alleviate these issues. Thus, ~~In this paper,~~ we focus on identifying patterns of ~~psychoactive substance~~PAS consumption using an

Formatted: Font color: Text 1
Formatted: Font color: Text 1
Formatted: Font color: Text 1
Formatted: Font color: Text 1
Formatted: Font color: Text 1
Formatted: Font color: Text 1
Formatted: Font color: Text 1
Formatted: Font color: Text 1
Formatted: Font color: Text 1, Not Expanded by / Condensed by
Formatted: Font color: Text 1
Formatted: Font color: Text 1, English (United States), Not Expanded by / Condensed by
Formatted: Font color: Text 1, Not Expanded by / Condensed by
Formatted: Font color: Text 1
Formatted: Font color: Text 1, English (United States), Not Expanded by / Condensed by
Formatted: Font color: Text 1, Not Expanded by / Condensed by
Formatted: Font color: Text 1
Formatted: Indent: First line: 0"
Formatted: Font color: Text 1, English (United States), Not Expanded by / Condensed by
Formatted: Font color: Text 1, Not Expanded by / Condensed by
Formatted: Font color: Text 1
Formatted: Font color: Text 1
Formatted: Font color: Text 1
Formatted: Font color: Text 1
Formatted: Font color: Text 1
Formatted: Font color: Text 1, Not Highlight
Formatted: Font color: Text 1
Formatted: Font color: Text 1, Not Expanded by / Condensed by
Formatted: Font color: Text 1
Formatted: Font: (Default) Times New Roman
Formatted: Font: (Default) Times New Roman
Formatted: Font: (Default) Times New Roman, English (Australia)

ensemble model ~~that integrates~~integrating an autoencoder with both a clustering algorithm and a spatial model. As part of our approach, we used the most recent and representative works for data clustering, ~~as well as~~and different dimensionality reduction and feature selection methodologies proposed in the literature.

~~F~~Feature selection approaches in clustering ~~for clustering~~ can be split into filter, wrapper, embedded, and hybrid approaches [37]. While wrappers depend on the clustering algorithms ~~of choice~~ to evaluate the clustering quality of a selected feature subset, filters are independent of the clustering algorithm. Embedded approaches also work with a clustering algorithm and, unlike wrappers, incorporate knowledge about the clustering structure. Another type of method~~s are~~ is hybrid approaches, which combine filter and wrapper approaches into a single strategy. However, studies on embedded and hybrid feature selection approaches in clustering are limited [37]. Other feature learning-based approaches using Deep Neural Networks have been shown to work well for linear and nonlinear models [56]. For instance, Xie et al. in (2016) ~~[57],~~ [57] propose to ~~simultaneously~~ work on feature extraction and clustering using pre-trained Auto-Encoders simultaneously. However, ~~Although deep clustering methods such as IDEC (Guo et al., 2017), DEPICT (Dizaji et al., 2017), DBC (F. Li et al., 2017), DualAAE (Ge et al., 2020), VAED (Lim et al., 2020), and DNC (B. Li et al., 2021) have been developed,~~ these are mainly used to work and process images. In general, deep clustering models use Auto-Encoders since they can learn input features without ~~having~~ labels on the data; performance measures show that this approach is reliable for different ~~types of~~ data types [58]. Thus, deep clustering methods have become a growing field of research for feature selection [58]. In this regard, the use of convolutional networks in autoencoders and the application of feature selection for clustering are open questions that have not been fully addressed yet, especially when dealing with data from different statistical distributions ~~In this regard, the use of convolutional networks in autoencoders and data from different statistical distributions, as well~~

Formatted: Font color: Text 1, Not Expanded by / Condensed by
Formatted: Indent: First line: 0"
Formatted: Font color: Text 1
Formatted: Font color: Text 1, English (United States), Not Expanded by / Condensed by
Formatted: Font color: Text 1
Formatted: Font color: Text 1
Formatted: Font color: Text 1
Formatted: Font color: Text 1
Formatted: Font color: Text 1
Formatted: Font color: Text 1, English (United States), Not Expanded by / Condensed by
Formatted: Font color: Text 1, English (United States)
Formatted: Font color: Text 1
Formatted: Font color: Text 1
Formatted: Font color: Text 1
Formatted: Font color: Text 1
Formatted: Font color: Text 1
Formatted: Font color: Text 1
Formatted: Font color: Text 1
Formatted: Font color: Text 1
Formatted: Font color: Text 1
Formatted: Font color: Text 1
Formatted: Font color: Text 1
Formatted: Font color: Text 1
Formatted: Font color: Text 1
Formatted: Font color: Text 1
Formatted: Font color: Text 1
Formatted: Font color: Text 1
Formatted: Font color: Text 1, English (United States), Not Expanded by / Condensed by
Formatted: Font color: Text 1
Formatted: Font color: Text 1, English (United States), Not Expanded by / Condensed by
Formatted: Font color: Text 1
Formatted: Font: (Default) Times New Roman
Formatted: Font: (Default) Times New Roman
Formatted: Font: (Default) Times New Roman, English (Australia)

~~as how to apply feature selection for clustering, remain open questions and have not yet been fully addressed~~ [37].

Here, we propose a Deep Neural Network-based Clustering-oriented Embedding Algorithm that allows us to (*i*) identify consumption patterns of ~~psychoactive~~ PAS~~substances~~; and (*ii*) build an ensemble algorithm integrating an autoencoder with a clustering algorithm and a spatial model to ~~jointly~~ deal with the feature space and cluster memberships. Our approach is based on the model proposed by Xie et al. ~~(2016)~~ [57]~~, [59]~~ and B. Li et al. ~~in (2021)~~ [59], and expands their work by creating an autoencoder from a convolutional network ~~instead of a linear one~~ to ~~both accurately~~ represent high-order interactions in the data accurately and simultaneously incorporate a spatial analysis to ~~properly~~ describe drug consumption patterns properly. Our main hypothesis is that incorporating these two critical~~key~~ elements in our proposal will help to identify and better understand drug consumption patterns and support national policy development processes.

Formatted: Font color: Text 1

Formatted: Font color: Text 1

Formatted: Font color: Text 1, English (United States), Not Expanded by / Condensed by

Formatted: Font color: Text 1

Formatted: Font color: Text 1

Formatted: Font color: Text 1

Formatted: Font color: Text 1, English (United States), Not Expanded by / Condensed by

Formatted: Font color: Text 1, English (United States)

Formatted: Font color: Text 1

Formatted: Font: (Default) Times New Roman

Formatted: Font: (Default) Times New Roman

Formatted: Font: (Default) Times New Roman, English (Australia)

2. ~~MATERIAL~~ Materials ~~AND METHODS~~and methods

2.1 Study a~~A~~rea

Located in South America, the ~~republic~~ Republic of Colombia is a diverse country with a population of over ~~48~~ 50 million people distributed ~~in~~ over a territory of 440,831 square miles [60], encompassing jungles, highlands, grasslands, deserts, coasts, and islands, distributed in six regions and 32 departments (states)[61]. It is worth noting that, unfortunately, ~~Regarding the drug situation in the study area,~~ Colombia has ~~long~~ been ~~known as~~ a major producer of illegal drugs for a long time~~.~~, which has had a significant impact on drug consumption and abuse.

According to the United Nations Office on Drugs and Crime, Colombia is the first cocaine-producing country~~,~~ and the eighth country with the highest production of cannabis [62]. In addition to this, ~~nowadays~~ ~~Regarding consumption, t~~the Colombian Drug Observatory indicates that the use of illicit substances in the territory has increased to 10.3%, with men between the ages of 18 and 24 ~~years old~~ being the heaviest consumers of these types of drugs. Reports also indicate that consumption of licit substances such as alcohol and tobacco ~~have~~ has recently ~~dramatically~~ increased dramatically [63].

2.2 Data s~~S~~ources

We used two databases ~~for~~ to identify ~~the analysis of~~ drug consumption patterns in Colombia. The first data~~-~~base was ~~obtained~~ retrieved from the 2019 National Survey of Psychoactive Substance Consumption in the General Population (DANE-DIMPE-ENCSPA-2019; URL: https://microdatos.dane.gov.co/index.php/catalog/680/data-dictionary) ~~during~~ ~~in 2019,~~ conducted by ~~Colombia's~~the ~~the~~ National Statistical System (DANE) of Colombia [64] ~~[14]~~. This ~~data~~ survey includes observations of 49,600 households, where information on housing, location, general characteristics of individuals, consumption of legal and illegal ~~psychoactive substances~~PAS, and

Formatted: Font: 18 pt
Formatted: Line spacing: Double
Formatted: Font: 16 pt
Formatted: English (United States)
Formatted: English (United States), Not Expanded by / Condensed by
Formatted: Font: 16 pt
Formatted: English (United States), Not Expanded by / Condensed by
Formatted: Font: (Default) Times New Roman
Formatted: Font: (Default) Times New Roman
Formatted: Font: (Default) Times New Roman, English (Australia)

implemented treatments is registered. ~~–~~The second data-base comes from the Colombian Drug Observatory and contains information on the production of ~~psychoactive substances~~PAS per area during 2019. All ~~T~~these databases ~~were~~are fully available and ~~fully available and~~completely anonymized. ~~were~~ In this study, we used departments (states) as ~~a~~ georeferenced areas using polygons (i.e., a shapefile) as implemented in ArcGIS Hub [65]. ~~Therefore~~Thus, ~~This~~this ~~research does not require~~ an ethics statement approved by an ethics committee is not required ~~-~~since we are using public information ~~where there is no~~without the identification or individual information of the people involved.

2.3~~3~~ Convolutional Auto-Encoder-Deep Embedded Clustering Algorithm

Formatted: Font: 16 pt

Formatted: Line spacing: Double

Fig~~ure~~ 1 presents the proposed Convolutional Auto-Encoder- Deep Embedded Clustering (CAE-DEC) framework~~, which is~~ based on the implementation presented by Xie et al. ~~(2016)~~[57]. However, unlike the Xie et al. ~~(2016)~~ model, our structure~~scheme~~ is ~~constructed~~ developed by applying convolutional layers for the ~~-~~deep autoencoder (DA) architecture instead of a linear one to represent high-order interactions in the data. In addition, a spectral clustering-based centroid estimation is proposed to achieve an improved initial centroid~~s~~ calculation. We chose the CAE-DEC- framework based on its ability to reduc~~ing~~e both the number of model parameters~~,~~ and ~~reducing~~the dimensionality, while ~~and~~ creating clusters simultaneously.

Formatted: Font: Bold, Font color: Blue

Formatted: Font color: Blue

Formatted: Font: Font color: Text 1, English (United States), Not Expanded by / Condensed by

Field Code Changed

Formatted: English (United States)

Formatted: Font: Font color: Text 1, English (United States), Not Expanded by / Condensed by

Formatted: Font: Font color: Text 1, Not Expanded by / Condensed by

Fig 1. Architecture of the proposed CAE-DEC model.

In our approach, an encoder structure is first applied to map the input vector into a lower feature space, called latent feature space (LFS). Then, the LFS is independently passed through a decoder structure and ~~through~~ a clustering layer to achieve an efficient clustering framework. The

Formatted: Font: (Default) Times New Roman

Formatted: Font: (Default) Times New Roman

Formatted: Font: (Default) Times New Roman, English (Australia)

encoder-decoder combination (~~, known as~~ DA)~~,~~ attempts to extract a LFS preserving the relevant information from the original input data. On the other hand, the clustering layer seeks to execute an improved clustering assignment by minimizing the divergence between a target distribution and a centroid-based probability distribution.

In the last stage of the framework~~As a complementary part of this research~~, a spatial analysis was performed using the feature space generated from the autoencoder as input. Here, the spatial data exploration is initially ~~explored~~ performed using ~~global~~ Global ~~spatial~~ Spatial ~~autocorrelation~~ Autocorrelation ~~in order~~ to determine to which ~~establish to which degree~~level the similarity between observations in a dataset relates to the similarity of the locations of such observations [66]. To assess ~~global spatial autocorrelation~~GSA, the Moran's I [67], Geary's *C* [68], and Getis and Ord's *G* [69] statistics are estimated. We also measure the Local Spatial Autocorrelation, which focuses on the relationships between each observation and its surroundings, rather than providing a single-number summary of these relationships across the map [70]. This is estimated based on the ability to determine whether spatial autocorrelation is present in a geographically referenced data set. Finally, we perform regionalization, which corresponds to a special kind of clustering where the objective is to group similar observations based on their statistical attributes and ~~their~~ spatial location [71]. In this sense, regionalization embeds the same logic as standard clustering techniques~~,~~ while applying a series of geographical constraints [72]. This framework was built using ~~two main librari~~the ~~es (~~TensorFlow (https://www.tensorflow.org/) and PyTorch~~)~~ (https://pytorch.org/) libraries ~~, through the~~in Python~~-programming language~~ version 3.11 ~~(V.3.11)~~ [73].

2.4~~1.~~ Convolutional Auto-Encoder (CAE)

Formatted: Font color: Text 1
Formatted: Font color: Text 1
Formatted: Font: (Default) Times New Roman
Formatted: Font color: Text 1
Formatted: Font: (Default) Times New Roman
Formatted: Font: (Default) Times New Roman
Formatted: Font color: Text 1
Formatted: Font color: Text 1
Formatted: Font color: Text 1
Formatted: Font: 16 pt
Formatted: Line spacing: Double
Formatted: Font: 16 pt
Formatted: Font: (Default) Times New Roman
Formatted: Font: (Default) Times New Roman
Formatted: Font: (Default) Times New Roman, English (Australia)

The DA is a deep neural network architecture capable of learning unsupervised representations of an input data set. Typically, DA networks are used for dimensionality reduction or denoising tasks. The structure of a DA is based on two deep networks: a network to transform the original input data into a latent feature space,~~;~~ and a network trained to reconstruct the original input data using the extracted latent space as input. The first network, used to extract the latent space, is called the encoder, while the second ~~network~~ is called the decoder. Rather than using fully connected layers, the implemented DA architecture incorporates convolutional (CONV) layers and fully connected (FC) layers for LFS extraction and reconstruction (**Fig~~ure~~ 1**). ~~The integration of~~Integrating convolutional layers in a DA is also called ~~convolutional auto-encoder (CAE)~~CAE [74]. Compared to a DA, which is built with only fully connected layers, the CAE structure can reduce the number of parameters compared to a DA [75].

2.4~~1~~.1 Convolutional l~~L~~ayer

The proposed CAE structure is designed using four convolutional layers, two CONV layers during the encoder stage, ~~and~~ two CONV layers during the decoder stage, and two fully connected layers (**Fig~~ure~~ 1**). The convolution operation can be denoted as:

$$z_{i,j,k}^{l} = W_{k}^{l^{T}} X_{i,j}^{l} + b_{k}^{l} \quad (1)$$

where $z_{i,j,k}^{l}$ is the value of each feature at the (*i*, *j*) location in the *k*-th feature map of the *l*-th layer, W_{k}^{l} and b_{k}^{l} represent the weight and bias of the *k*-th filter of the *l*-th layer, and $X_{i,j}^{l}$ denotes the input value at location (*i*, *j*) of the *l*-th layer. For non-linear mapping, an activation function *g*(.) is applied over the convolutional feature $z_{i,j,k}^{l}$ as follows:

$$a_{i,j,k}^{l} = g(z_{i,j,k}^{l}) \quad (2)$$

where $a_{i,j,k}^{l}$ is the activation value resulting from applying the activation function *g*(.). The Rectified Linear Unit (ReLU) function is set as the activation function on each CONV, except in the final decoder CONV layer where a sigmoidal activation ~~will be~~is applied.

Formatted: Font: Bold, Font color: Blue

Formatted: Font: English (United States), Not Expanded by / Condensed by

Formatted: Font: English (United States), Not Expanded by / Condensed by

Formatted: Font: 14 pt

Formatted: Font: 14 pt

Formatted: Font: Bold, Font color: Blue

Formatted: Font: Italic

Formatted: No underline

Formatted: Font: (Default) Times New Roman

Formatted: Font: (Default) Times New Roman

Formatted: Font: (Default) Times New Roman, English (Australia)

2.5~~2~~ Clustering l~~L~~ayer

The clustering layer is inspired by Xie et al. [57] ~~Xie et al. (2016)~~. Initially, a soft assignment is computed between the latent space, also known as embedded space, and the cluster centroids. Then, update steps are repeated to define the final cluster centroids and embedded space. The Kullback–Leibler (KL) divergence is used as loss function during the optimization procedure. The objective is to minimize de KL divergence between a soft clustering distribution Q and an auxiliar target distribution P. The KL loss is calculated as:

$$L_c = KL(P\|Q) = \sum_k \sum_m p_{km} log\left(\frac{p_{km}}{q_{km}}\right) ~~\frac{p_{km}}{q_{km}}~~ \quad (3)$$

where L_c is the clustering loss. To measure the similarity between embedded point z_k and the cluster centroid c_m ~~cm~~, the t Student's distribution is used as a kernel:

$$q_{km} = \frac{(1 + \|z_k - c_m\|^2/\alpha)^{-\frac{\alpha+1}{2}}}{\sum_i (1 + \|z_k - c_i\|^2/\alpha)^{-\frac{\alpha+1}{2}}} \quad (4)$$

with α the degrees of freedom of the t Student's distribution and q_{km} is a ~~a~~ soft clustering assignment distribution of each embedded point (i.e., probability of assigning point k to ~~the~~ cluster m). As in Xie et al. ~~(2016)~~, when setting α=1 the similarity function q_{km} can be calculated as:

$$q_{km} = \frac{(1 + \|z_k - c_m\|^2)^{-1}}{\sum_i (1 + \|z_k - c_i\|^2)^{-1}} \quad (5)$$

To compute the target distribution p_{km}, the second power of q_{km} ~~second power~~ is ~~defined~~calculated, and a cluster normalization is applied as follows:

$$p_{km} = \frac{q_{km}^2 / \sum_k q_{km}}{\sum_i (q_{ki}^2 / \sum_k q_{ki})} \quad (6)$$

Then, by minimizing the divergence between P and Q, the embedding learning is achieved through highly confident assignments.

2.5~~2~~.1 Center i~~I~~nitialization

Formatted: Font: 16 pt

Formatted: Indent: First line: 0"

Formatted: Indent: First line: 0"

Formatted: Font: 14 pt

Formatted: Font: 14 pt

Formatted: Font: (Default) Times New Roman

Formatted: Font: (Default) Times New Roman

Formatted: Font: (Default) Times New Roman, English (Australia)

As previously mentioned, the cluster centroids are initialized using a spectral clustering-based approach. The spectral clustering allows flexible distance metrics and provides better cluster estimations than ~~k~~K-means ~~-~~[57]~~(Xie et al. 2016)~~. However, most spectral clustering algorithms have high computational requirements. To overcome these computational requirements, random samples are ~~be~~ taken to estimate the cluster centroids. As spectral clustering does not estimate any centroid during the learning process, once the clusters are defined, the mean of each cluster is used as the centroid estimator.

2.6~~3~~ The CAE-DEC ~~M~~model

Initially, the input data ~~are~~ is normalized within the interval [0, 1]. This normalization allows the network to use the most advanced learning rate and avoid the vanishing ~~o~~ gradient problems, as well as ~~likewise to~~ alleviate over~~-~~fitting. Further, to achieve a better learning process, the last CONV layer in the decoder structure is activated by a sigmoid activation function. Then, two training steps will be executed during the CAE-DEC learning process. Firstly, a CAE model will be trained to minimize the reconstruction loss L_r ~~as~~ computed as~~follows:~~

$$L_r = \|x - \tilde{x}\|^2 \quad (7)$$

where x is the normalized input and $\tilde{x}$ is the reconstructed output. This pretrained CAE model is then used as the DA structure in the CAE-DEC model.

In the second step, the CAE-DEC model is trained to simultaneously minimize reconstruction loss and clustering loss. The total loss during this training step will be set as

$$L_t = L_r + C \cdot L_c \quad (8)$$

where L_r is the CAE-DEC reconstruction loss, L_c is the CAE-DEC clustering loss, and C is a coefficient to control the loss balance. The training process is shown in **Table 1**. The goal is to obtain a latent space that minimizes the total loss. Finally, the ~~, and the~~ label of each embedded point ~~must be established. The final label will be obtained as~~is established as~~—~~

$$Label_j = \arg max_m q_{jm} \quad (9)$$

Formatted: Font: 16 pt

Formatted: Font: 16 pt

Formatted: Font: Bold, Font color: Blue

Formatted: Font: (Default) Times New Roman

Formatted: Font: (Default) Times New Roman

Formatted: Font: (Default) Times New Roman, English (Australia)

Table 1. Pseudo code for the CAE-DEC training process.

```
Pseudo code: The CAE-DEC training process
Input data: Number of clusters n; Normalized input data x; Maximum number of iterations Mint; Balance
coefficient C; Pretrained CAE; Stop condition Stop; Target distribution P update condition P_change.
Training process:
1. Generate an initial latent space (Z) through the pre-trained CAE
2. Run spectral clustering with Z to generate the initial cluster centers (C)
3. Initialize the CAE-DEC model with the pretrained CAE.
4. Calculate soft assignment distribution Q and target distribution P based on Z and C
for epoch<Minter do:
    if epoch%P_change==0 then:
        Calculate soft assignment distribution Q and target distribution P based on Z and C
    end if
    Feed the CAE-DEC with the normalized input data x
    Calculate the reconstruction loss and the clustering loss
    Update CAE-DEC parameters. Weight, Bias, and Centers.
    if Stop==True then:
        Break
    end if
end for
Obtain the label for each data point from the las optimized Q.
Output: Latent space, labels
```

where ~~the~~ q_{jm} ~~value can be seen as~~ is the probability that point *j* belongs to a specific cluster center *m*. On the other hand, the maximum number of iterations `Mint` and the target distribution `P` update condition `P_change` ~~were~~was chosen based on multiple experiments. The final `Mint` and `P_change` values were 3000 and 5, respectively. This final `P_change` improves stability during the training process.

2.7 Framework ~~e~~Evaluation

We trained the CAE-DEC method using ~~the database~~data retrieved from the National Survey of Psychoactive Substance Consumption (DANE-DIMPE-ENCSPA-2019), which contains 49,600 observations. ~~T~~A ~~he~~ second database with ~~(psychoactive substances~~PAS production figures~~)~~, was used in the spatial analysis stage to correlate the ~~SPA~~PSA consumption and production. In order to evaluate the framework, we compared our CAE-DEC approach ~~(CAE-DEC) against~~with other approaches, including CAE, and Principal Component Analysis integrated with clustering (PCA-DEC). For evaluation and comparison purposes, ~~W~~we use ~~the intrinsic clustering metric for~~

Formatted: Indent: First line: 0"

Formatted: Indent: First line: 0.5"

Formatted: Font: (Default) Courier New

Formatted: Font: (Default) Courier New

Formatted: Font: (Default) Courier New

Formatted: Font: (Default) Courier New

Formatted: Font: (Default) Courier New

Formatted: Font: (Default) Courier New

Formatted: Font: 16 pt, Bold

Formatted: Font: 16 pt, Bold

Formatted: Font: 16 pt

Formatted: Font: 16 pt, Bold

Formatted: Normal (Web), Justified, Line spacing: Double

Formatted: Font: (Default) Times New Roman

Formatted: Font: (Default) Times New Roman

Formatted: Font: (Default) Times New Roman, English (Australia)

evaluations and comparisons to other approaches, like the Calinski-Harabasz [76], Davies-Bouldin [77], and sSilhouette [78] index as intrinsic clustering metrics. In addition, we used the chi-square (χ^2) statistic to investigate potential associations and differences among validate whether the patterns (clusters) identified found inusing our approach each cluster are related or not, i.e., they are independent.

Formatted: Font: (Default) Times New Roman

Formatted: Font: (Default) Times New Roman

Formatted: Font: (Default) Times New Roman, English (Australia)

.

Formatted: Font: (Default) Times New Roman

Formatted: Font: (Default) Times New Roman

Formatted: Font: (Default) Times New Roman, English (Australia)

3. ~~RESULTS~~Results

3. 1 Model Comparison for Identifying Drug Consumption Patterns

Fig~~ure~~ 2 depicts the LFS resulting after applying ~~both~~ the CAE and CAE-DEC models to the data. Among all individuals, we identified three different clusters; 14935 (30.19%) individuals belong to cluster 0, 11528 (23.30%) individuals belong to cluster 1, and 23005 (46.50%) individuals belong to cluster 2. Interestingly, the LFS generated with the CAE-DEC has more defined clusters ~~compared to that generated by~~than the CAE model. Although the CAE model seeks to extract a LFS that preserves the essential~~important~~ characteristics of the input data, our proposed CAE-DEC model not only preserves these important characteristics but, at the same time, also forces the encoder structure to generate representative clusters while extracting the new feature space.

On the other hand, the reconstruction loss obtained through the CAE model is higher than that of the CAE-DEC model. This result may be related to the fact that the CAE-DEC model used the pre-trained CAE model during its construction. It should be noted that the CAE model alone ~~is not able to~~cannot determine the labels of each point or define clusters in the data. Thus, clusters ~~showed~~ in **Fig~~ure~~ 2** were obtained through spectral clustering and were the bases for initializing the centroids in the CAE-DEC model.

Fig 2. Derived Latent Feature Space based on the (a) CAE and (b) CAE-DEC models.

3.2 Identification of Clusters of Psychoactive Drugs Consumption

Here we ~~analyzse~~analyse the patterns ~~within~~ in each ~~of the~~ cluster~~s~~ obtained using the CAE-DEC model. In particular, we defined a priority dummy variable Y_{ij} quantifying whether the *i*th person in household *j*th has consumed ~~psychoactive substances~~PAS; $Y_{ij} = 1$ when an individual has never consumed ~~psychoactive substances~~PAS and $Y_{ij} = 2$ ~~when the individual has consumed one or more substance~~otherwise~~s (i.e., tranquilizers, stimulants, methadone or opioid analgesics without a~~

Formatted: Font: 18 pt

Formatted: Font: Bold, Font color: Blue

Formatted: Font color: Blue

Formatted: Font: Bold, Font color: Blue

Formatted: Font color: Blue

Formatted: Cuerpo, Line spacing: 1.5 lines

Formatted: Font: Bold, German (Germany)

Formatted: Font: 16 pt

Formatted: No underline

Formatted: Font: (Default) Times New Roman

Formatted: Font: (Default) Times New Roman

Formatted: Font: (Default) Times New Roman, English (Australia)

~~medical prescription, or inhalants, DICK, popper, marijuana, cocaine, bazuco, ecstasy, heroin, methamphetamine, Lysergic acid diethylamide [LSD], mushrooms, yage, cacao sabanero, ketamine, Gamma-hydroxybutyrate [GHB], 4-Bromo-2,5-dimethoxyphenethylamine [2CB] or any other drugs)~~.

Out of the 49468 individuals in the sample, only 5514 (11.15%) consume ~~psychoactive substances~~PAS. **Fig~~ure~~ 3a** and **Fig 3b** depict, respectively, the derived cluster structure ~~derived with the CAE-DEC model~~ for individuals consuming ~~psychoactive substances~~PAS and those who reported not ~~to consume~~consuming, derived ~~with~~from the CAE-DEC model. Our results indicate that individuals ~~belonging to~~in clusters 0 and 2 are more likely to consume some ~~type of psychoactive~~ PAS~~substances~~ (**Fig~~ure~~ 3a**), while most individuals in cluster 1 do not ~~consume psychoactive substances~~ PAS (**Table ~~1~~2**). In particular, 1726 (11.56%) individuals in cluster 0, 392 (3.4%) individuals in cluster 1, and 3396 (14.76%) individuals in cluster 2 have used ~~psychoactive substances~~PAS (**Table ~~1~~2**). A χ^2-based test of independence reveals that the region where individuals are located, age (years), the type of household they live in, their socioeconomic~~al~~ status (SES), and whether they contribute to the household finances~~,~~ are statistically significantly associated ~~to~~ with the cluster they belong to (**Table 2**).

Fig 3. Resulting clusters for individuals (a) consuming and (b) not consuming psychoactive substances based on the CAE-DEC model.

Table 2. Distribution of demographic and social variables across clusters.

Variables		Cluster 0 (*n*=14935)	Cluster 1 (*n*=11528)	Cluster 2 (*n*=23005)	χ^2	*df*	*P*-value
	Caribbean	3004	2991	4075			
Region	Central-Eastern	2526	2299	6175	1472.9	10	<.0001
	Central-Southern	1843	1299	1702			

Formatted: Font: Bold, Font color: Blue
Formatted: Font color: Blue
Formatted: Font: Bold, Font color: Blue
Formatted: Font: Bold, Font color: Blue
Formatted: Font: Bold, Font color: Blue
Formatted: Font: Bold, Font color: Blue
Formatted: Font: Bold, Font color: Blue
Formatted: Font: (Default) Times New Roman
Formatted: Font: (Default) Times New Roman
Formatted: Font: (Default) Times New Roman, English (Australia)

	Eje Cafetero – Antioquia	3902	2354	6371			
	Llanos Orientales	1687	1305	1496			
	Pacific	1973	1280	3186			
Gender	Male	6606	3927	10233	386.72	2	<.0001
	Female	8329	7601	12772			
Housing type	House	8250	6538	11834	114.18	6	<.0001
	Apartment	6275	4724	10595			
	Room	395	256	543			
	Indigenous dwelling	15	10	33			
Socioeconomic status	1	3889	3626	6945	843.92	10	<.0001
	2	4627	3902	8851			
	3	4284	2867	5683			
	4	1341	718	979			
	5	510	256	362			
	6	284	159	185			
Age (years)	(0, 20]	2105	1576	2998	345.87	4	<.0001
	(20, 40]	6814	4329	10889			
	(40, 68]	6016	5623	9118			
Contribute to the household finances	Yes	10128	7473	16041	85.24	2	<.0001
	No	4807	4055	6964			

df: Degrees of freedom.

Table 3 shows the adjusted residuals for our model. According to our results, the Central-Eastern region significantly contributes to the Region variable ~~the greatest contribution of the Region variable is made by the Central-Eastern region~~. In this region, the observed value is higher than the expected value in cluster 2, while the observed value is lower than the expected value for cluster 0. Although to a lesser extent, the Llanos Orientales region also significantly contributes~~shows a large contribution to~~ the χ^2 statistic. Indeed, this region shows fewer~~shows a lower number of~~ observed individuals ~~compared to~~than the expected number of individuals in cluster 2 and a higher number observed than expected individuals in clusters 0 and 1 (**Table 3**).

Formatted: Font: (Default) Times New Roman, 12 pt, Bold, Font color: Blue

Formatted: Font: (Default) Times New Roman, 12 pt, Font color: Blue

Formatted: Font: (Default) Times New Roman, 12 pt

Formatted: Font: 12 pt

Formatted: Font: 12 pt

Formatted: Font: (Default) Times New Roman, 12 pt, Bold, Font color: Blue

Formatted: Font: (Default) Times New Roman

Formatted: Font: (Default) Times New Roman

Formatted: Font: (Default) Times New Roman, English (Australia)

Table 3. Adjusted residuals comparing the observed and expected frequencies based on the cluster analysis.

	Variables	Cluster 0 (*n*=14935)	Cluster 1 (*n*=11528)	Cluster 2 (*n*=23005)
Region	Caribbean	-0.88	17.02	-13.61
	Central-Eastern	-18.72	-6.76	22.97
	Central-Southern	12.54	6.09	-16.7
	Eje Cafetero - Antioquia	2.02	-14.36	10.31
	Llanos Orientales	11.32	9.59	-18.55
	Pacific	0.84	-6.97	5.13
Gender	Male	6.68	-19.66	10.52
	Female	-6.68	19.66	-10.52
Housing type	House	4.17	7.13	-9.88
	Apartment	-4.83	-6.61	10.05
	Room	2.2	-1.54	-0.72
	Indigenous dwelling	-0.72	-1.09	1.59
Socioeconomic status	1	-10.26	5.99	4.37
	2	-12.72	-3.3	14.51
	3	9.14	-3	-5.87
	4	17.29	0.44	-16.29
	5	11.12	-0.49	-9.82
	6	8.26	1.2	-8.62
Age (years)	(0, 20]	2.54	0.61	-2.85
	(20, 40]	3.2	-17.23	11.66
	(40, 68]	-4.98	16.93	-9.77
Contribute to the household finances	Yes	-0.61	-8.37	7.65
	No	0.61	8.37	-7.65

On the other hand, Gender has a higher-than-expected value of males in clusters 0 and 2, while it is lower in cluster 1. For females, the opposite occurs in cluster 1, and lower values are observed in clusters 0 and 2. Similarly, Housing Type has a higher-than-expected value of individuals living at houses in cluster 1~~,~~ and a lower-than-expected in cluster 2. Conversely, cluster 2 has more~~a higher number of~~ individuals living in apartments, and cluster 1 has the lowest (**Table 3**).

Formatted: Indent: First line: 0"

Formatted: Font: (Default) Times New Roman, 12 pt, Bold, Font color: Blue

Formatted: Font: (Default) Times New Roman

Formatted: Font: (Default) Times New Roman

Formatted: Font: (Default) Times New Roman, English (Australia)

Regarding ~~the SES~~SES, ~~we found ~~a higher-than-expected number of individuals in strata 3, 4, 5, and 6 in cluster 0 w~~as~~ere found (**Table 3**). We also observed a lower-than-expected number of individuals in strata 3, 4, 5, and 6 in cluster 2~~,~~ and a higher-than-expected number ~~of individuals ~~in strata 1, 4 and 6 in cluster 1 (**Table 3**). Moreover, the age variable shows a higher-than-expected observed value for the (0,20] range in cluster 0. For ages between (20,40] years, cluster 2 has a higher-than-expected number of individuals. Conversely, there is a lower number of individuals in cluster 1. Finally, ~~the results for ~~the household economy variable results show that cluster 2 has a higher-than-expected value of individuals contributing to the household finances, and cluster 1 has a lower-than-expected value of individuals not contributing to it. Comparison of Calinski-Harabasz~~Calinski~~, Davies-Bouldin~~Davies~~, and silhouette metrics between a principal component analysis (PCA)-based deep autoencoder (PCA-DEC) and our proposed CAE-DEC model indicates the superiority of the latter (~~**Table 2S, Supplementary Material**~~~~**S2**~~**Table S2, Supplementary Material**~~**Table**~~).

3.3 Spatial Analysis of Psychoactive Drugs Consumption

Different alternative classification algorithms were used to determine the number of choropleth class limits ~~To determine the number of choropleth class limits, different alternative classification algorithms were used ~~(i.e., Equal Intervals, Quantiles, Maximum Breaks, Box plot, Head-Tail Breaks, Jenks-Caspall, Fisher-Jenks, and Max-p) and compared using the absolute deviation around class medians optimization criterion (**Fig~~ure~~ 4**). According to our results, the Fisher-Jenks classifier performed better and hence was selected.

Fig 4. Absolute deviation around class medians (ADCM) statistic criterion for different alternative classifiers. Here, lower is better.

Formatted: Font: Bold, Font color: Blue

Formatted: Font: Bold, Font color: Blue

Formatted: Font color: Blue

Formatted: Font: (Default) Times New Roman, 12 pt

Formatted: Font: 16 pt

Formatted: Font: Bold, Font color: Blue

Formatted: Font: (Default) Times New Roman

Formatted: Font: (Default) Times New Roman

Formatted: Font: (Default) Times New Roman, English (Australia)

Following the same exploratory spatial analysis, we constructed a choropleth with the percentage of PAS ~~psychoactive substance~~ use for each of the 32 Colombian departments (**Fig~~ure~~ 5a**). ~~Our results indicate~~We found that~~ that~~ the departments of Arauca, Vichada, Caquetá, Chocó, Magdalena, Cesar, Bolivar, Sucre, Cordoba, and Norte de Santander have low percentages of drug use. However, some of these departments are major drug producers (i.e., Cordoba and Guaviare), according to data from the Drug Observatory of Colombia [79]. Similarly, Putumayo is the department with the highest proportions of ~~psychoactive substance~~PAS use (**Fig~~ure~~ 5a**).

Fig 5. (a) Consumption percentage of psychoactive substances according to the CAE-DEC model; (b) Moran's I statistic; (c) Moran's cluster map. Here, HH, LH, LL and ns represent high-high, low-high, low-low, and not statistically significant quadrants, respectively. This clustering pattern leads to a statistically significant Moran's I statistic of 0.2 (*P*-value <0.01).

The global Moran's I results~~Results of the global Moran's I~~ show the presence of a statistically significant positive global spatial autocorrelation (I = 0.2005, *P*<0.01). Thus, the null hypothesis that the map is random (i.e., that the map shows more spatial patterns than we would expect if the values had been randomly assigned to a location) is rejected. In addition, other global indices such as Geary's *C* (*C* = 0.693, *P* = 0.003)~~,~~ and Getis and Ord's *G* (*G* = 0.800, *P* = 0.049) confirm the presence of statistically significant global spatial autocorrelation.

To further explore the relationships between each observation and its environment, the Local Indicators of Spatial Association (LISA) were estimated (more information on LISA statistics is provided in ~~**Figure 2S** of the **Supplementary Material**~~~~S~~**Fig S42, Supplementary Material** ~~Fig~~). **Fig~~ure~~ 5b** depicts the Moran diagram, indicating each quadrant's positive (or negative) association~~the positive (or negative) association within each quadrant~~. Specifically, the high-high (HH) and low-low (LL) quadrants indicate a positive association between~~with~~ high and low drug use~~, respectively~~. On the other hand, the low-high (LH) and high-low (HL) quadrants indicate negative associations ~~of~~ with drug use (**Fig~~ure~~ 5b**). Following our results, we found that

Formatted: Font: Bold, Font color: Blue

Formatted: Font: Bold, Font color: Blue

Formatted: Indent: First line: 0.5"

Formatted: Font color: Blue

Formatted: Font: Bold, Font color: Blue

Formatted: Font color: Blue

Formatted: Font: Bold, Font color: Blue

Formatted: Font: (Default) Times New Roman

Formatted: Font: (Default) Times New Roman

Formatted: Font: (Default) Times New Roman, English (Australia)

departments such as Nariño and Cauca belong to the HH ~~(hot spot)~~ cluster. In contrast,~~, while~~ l~~L~~a Guajira, Atlántico, Magdalena, Cesar, Norte de Santander, Sucre, and Cordoba belong to the LL ~~(cold spot)~~. This clustering pattern leads to a statistically significant Moran's I statistic (*P*-value <0.01). Thus, a little over 39.4% of the departments are considered, by this analysis, to be part of a spatial cluster (i.e., statistically significant with a *P*-value <5%).

We also identified that, ~~**Figure 6** shows the illegal and legal drugs more frequently consumed in Colombia.~~ a~~A~~mong legal drugs, alcohol and tobacco are the most frequently consumed in the national territory (**Fig 6a**~~Figure 6a~~). At the same time,~~, while~~ marijuana, followed by non-prescription tranquilizers and Yagé, and a slight consumption of opioids and Poppers, are the most frequently consumed illegal drugs (**Fig~~ure~~ 6b**).

Fig 6. Frequency of consumption of (a) legal and (b) illegal drugs in Colombia.

Regarding~~In terms of~~ legal drugs, alcohol has the highest consumption rates in Bogotá, Cundinamarca, and Chocó (**Fig~~ure~~ 7**). However, there is moderately high use in Vaupés, Nariño, Bolívar, Magdalena, La Guajira, and Atlántico (**Fig~~ure~~ 8**). As for energy drinks, consumption is the highest in Casanare and Guaviare~~,~~ and has slightly high uses in Boyacá, Nariño, Risaralda, and Arauca. On the other hand, tobacco has the highest consumption in Cundinamarca~~,~~ but has moderately high uses in Bogotá, Boyacá, Nariño, Casanare, Tolima, Quindío, Risaralda, Guainía, Caldas, and Vaupés. It ~~should also~~should ~~-~~be mentioned that the use of these drugs is also present across the country~~,~~ but with a lower incidence (**Fig~~ure~~ 7**).

Fig 7. Consumption of illegal drugs by department. For interpretation purposes, number represents values scaled on a range of 0 to 1. For instance, Bogotá D.C. has the highest LSD consumption and Putumayo has the lowest.

Formatted: Font: Bold, Font color: Blue
Formatted: Font color: Blue
Formatted: Font: Bold
Formatted: Font: Bold, Font color: Blue
Formatted: Indent: First line: 0.5"
Formatted: Font: Bold, Font color: Blue
Formatted: Font: Bold, Font color: Blue
Formatted: Font: Bold, Font color: Blue
Formatted: Indent: First line: 0.5"
Formatted: Font: (Default) Times New Roman
Formatted: Font: (Default) Times New Roman
Formatted: Font: (Default) Times New Roman, English (Australia)

Concerning~~Regarding~~ illegal drugs, non-prescription tranquilizers and stimulants are most prevalent in Casanare (**Fig~~ure~~ 8**). However, the consumption of tranquilizers is slightly higher in Nariño, while inhalants have the highest consumption in ~~Quindio~~Quindío, followed by Cauca, Caldas, and Nariño. Methylene Chloride (DICK) has the highest consumption~~use~~ in Cauca and a high consumption in ~~Quindio~~Quindío and Nariño;~~ while ~~Antioquia, followed by Caldas and Risaralda, shows the highest consumption of popper. On the contrary, marijuana has its highest consumption in Risaralda and moderately high consumption in Caldas, Bogotá, Antioquia, and Quindío. As for cocaine, its consumption is the highest in Risaralda and moderately high in Antioquia (**Fig~~ure~~ 8**).

Fig 8. Consumption of legal drugs by department. Number represents values scaled on a range of 0 to 1 for psychoactive substance use. Conventions as in Fig 7.

On the other hand, bas~~z~~uco (i.e., cocaine paste) has the highest consumption rate in Guaviare, and critical consumption in Nariño, Cauca, ~~Quindio~~Quindío, Antioquia, and Amazonas; ecstasy has its highest consumption in Risaralda, followed by Bogotá and Caldas; heroin consumption is highest in Vaupes, Huila, Cauca, ~~Quindio~~Quindío, and Arauca, and is slightly higher in Casanare; methamphetamine consumption is highest in Casanare and is moderately high in Boyacá; methadone is most widely used in ~~Quindio~~Quindío, but has slightly high levels of use in Valle del Cauca and Caquetá; opioids are most prevalent in Casanare, followed by Sucre; LSD is most prevalent in Bogotá, but has high levels of use in Caldas, Risaralda, ~~Quindio~~Quindío, and Nariño; mushrooms have their highest consumption in Boyacá and have moderately high uses in ~~Quindio~~Quindío, Risaralda, Bogotá, Cauca, and Casanare; Yagé has a higher incidence in Putumayo; cacao sabanero has its highest consumption in Caldas, and has moderate consumption in Cundinamarca, Bogotá, Antioquia, and ~~Quindio~~Quindío; ketamine has the highest consumption in Casanare, followed by Antioquia; and GHB has the highest consumption in Risaralda, followed by Santander, Valle del Cauca, and Norte de Santander. Finally, 2CB has the highest consumption rate

Formatted: Font: Bold, Font color: Blue

Formatted: Font: Bold, Font color: Blue

Formatted: Indent: First line: 0.5"

Formatted: Font: (Default) Times New Roman

Formatted: Font: (Default) Times New Roman

Formatted: Font: (Default) Times New Roman, English (Australia)

in Risaralda, followed by Caldas. Although the consumption pattern of some departments is not mentioned, there is low and moderate consumption for certain drugs in some of them (**Fig~~ure~~ 8**).

3.4 Regionalization of Clusters

We applied a regionalization method as a grouping technique for imposing a spatial restriction, i.e., the result of a regionalization algorithm contains clusters with geographically coherent areas~~, in addition to having~~ and coherent data profiles. Our approach uses a spatially constrained hierarchical clustering algorithm, which identified three clusters representing the consumption of ~~psychoactive substances~~PAS in the country (**Fig~~ure~~ 9**). The number of clusters was estimated based on the average silhouette indexes, the total intra-cluster variance, and dendrograms (~~**Figure 1S, Supplementary Material**~~~~**S3**~~ **Fig S1, Supplementary Material**). Following our results, cluster 0 is comprised ~~by~~ of departments such as La Guajira, Cesar, Atlántico, Magdalena, Norte de Santander, Bolivar, Sucre, and Cordoba, all of them located ~~at~~ in the Northern region of the country; cluster 1 is comprised ~~by~~ of Antioquia, Santander, Boyacá, Caldas, Risaralda, and Quindío; and cluster 2 is integrated by the remaining departments (**Fig~~ure~~ 9**). When testing geographical coherence, which is the measure that assesses the "compactness" of a given shape, our results indicate that the clusters derived using the regionalization model represent moderately compact regions. In addition, the feature coherence (i.e., goodness-of-fit) test using different metrics showed that our 3-cluster regionalization structure properly fits the data (~~**Table 1S, Supplementary Material**~~~~**S1**~~ **Table S1, Supplementary Material**).

Fig 9. Cluster map of drug use after regionalization.

Formatted: Font: Bold, Font color: Blue
Formatted: Indent: First line: 0.5"
Formatted: Font: 16 pt
Formatted: Font: Bold, Font color: Blue
Formatted: Font color: Blue
Formatted: Font: Bold, Font color: Blue
Formatted: Font color: Blue
Formatted: Font: Not Bold, Font color: Text 1
Formatted: Indent: First line: 0.5"
Formatted: Font: (Default) Times New Roman
Formatted: Font: (Default) Times New Roman
Formatted: Font: (Default) Times New Roman, English (Australia)

4. ~~DISCUSSION~~Discussion

In this study, we ~~a~~ propose and test a Deep Neural Network-based Clustering-oriented Embedding algorithm (i.e., a ML-based model) for identifying ~~patterns of~~ psychoactive substance (PAS) use and abuse patterns in ~~the~~ Colombia~~n context~~. This model allows the automatic extraction of features from the input data (such as sex, age, socioeconomic status, and housing type) to determine whether an individual has consumed PAS. It then creates clusters in the new data space generated during the learning process, following the methods outlined in~~This model allows to automatically extract features from the input data (i.e., sex, age, socioeconomic status, housing type, among others) to determine whether an individual has consumed psychoactive substances, and creates clusters in the new data space generated during the learning process as proposed by~~ [57~~]~~, ~~[~~59] ~~B. Li et al., 2021 and Xie et al., 2016~~. After the training process, a latent feature space (LFS) is generated~~,~~ and the ~~analysis of the~~ results ~~is~~ are subsequently ~~performedz~~analysed.

We have identified clearly marked clusters where the prevalence of individuals who use or do not use PAS is notable. Additionally, we found that region, sex, housing type, socioeconomic strata, age, and whether individuals contribute to household finances have a statistically significant impact on the clustering structure. These findings are consistent with previous studies aimed at identifying PAS consumption patterns~~Among the main results, we highlight the existence of clearly marked clusters where the prevalence of individuals who use or do not use psychoactive substances is identified. In addition, we identified that region, sex, housing type, socioeconomic status, age, and whether individuals contribute to the household finances are statistically impacted to the clustering structure. This result is in line with previous studies aimed at identifying consumption patterns of psychoactive substances~~ [19~~]~~, ~~[~~80~~]~~, ~~[~~81]. Interestingly, when comparing the CAE-DEC model proposed ~~herein~~ in this study and the CAE-Spectral model using different ~~score~~ metrics (i.e., Silhouette ~~score~~statistic, which measures the internal density of each cluster and the distance that separates them from each other, the Calinski-Harabasz index and the Davies-Bouldin index [DBI]), ~~showed that t~~we found that our model ~~he our proposal model pp~~erforms better (Silhouette: 0.62 vs.

Formatted: Font: 18 pt

Formatted: Font color: Text 1, English (Australia), Not Expanded by / Condensed by

Formatted: Font color: Text 1, English (Australia), Not Expanded by / Condensed by

Formatted: Indent: First line: 0.5"

Formatted: Font color: Text 1, English (Australia), Not Expanded by / Condensed by

Formatted: Font color: Text 1, English (Australia), Not Expanded by / Condensed by

Formatted: Font: (Default) Times New Roman

Formatted: Font: (Default) Times New Roman

Formatted: Font: (Default) Times New Roman, English (Australia)

0.786; Calinski-Harabasz: 22468.26 vs. 775992.45; DBI: 0.2898 vs. 0.63; S2 **Table** ~~S2~~, **Supplementary Material**)~~, kindly see S2 table for more details.~~. ~~than the CAE-Spectral alone model.~~

~~Following~~ Based on our findings~~the results of our model~~, ~~we~~ individuals more likely to consume PAS are grouped in cluster 2, while ~~found that individuals more likely to have consumed psychoactive substances are grouped in cluster 2 (**Table 1**). On the other hand,~~ cluster 1 ~~is~~ consisted of individuals ~~comprised of indi~~who did no~~viduals that do no~~t consume ~~psychoactive substances~~PAS (**Table 1**). Not surprisingly, ~~A~~a ~~more~~ significant proportion of females characterizes ~~this~~cluster 1~~ latter cluster.~~~~This latter cluster is characterized by a greater proportion of females,~~ In addition, ~~M~~most individuals belong to socioeconomic strata 1, are 40 years old or older, and do not contribute economically to support their household~~most belong individuals belong to socioeconomical strata 1, are 40 years of age or older, and do not contribute economically to the support their household~~. In contrast, cluster 2 is characterized by a higher proportion of males aged between 20 and 40~~, a higher proportion of individuals~~ in socioeconomical strata 1 and 2, ~~with aged~~s ~~between 20 and 40 years, that~~ who do not contribute to the household ~~economy~~ finances (**Table 1**). Finally, cluster 0 is characterized by a small proportion of males, a higher proportion of individuals in strata 3, 4, 5, and 6, and individuals are ~~that are~~ more likely to contribute to the household economy (**Table 1**).

At the level of spatial statistics, we identified that legal drugs such as alcohol have a high prevalence in all regions of Colombia, with a slight tendency to more consumption in coastal areas (**Fig~~ure~~ 7**). In ~~Colombia~~our country,~~,~~ the coastal areas are often popular tourist destinations, and many tourists come to these areas ~~to~~looking for a relaxing experience~~ relax and have fun~~, which~~.~~ ~~This~~ can increase alcohol consumption~~ as people let loose and enjoy their vacation~~. Coastal areas typically have warmer temperatures and more sunshine, increasing thirst and making people more likely to consume alcohol. Additionally, ~~coastal areas may have more~~ bars, clubs, and restaurants

Formatted: Font: Bold, Font color: Blue

Formatted: Font: Not Bold, Font color: Text 1

Formatted: Font: Bold, Font color: Blue

Formatted: Font: Bold, Font color: Blue

Formatted: Font: Bold, Font color: Blue

Formatted: Font: Bold, Font color: Blue

Formatted: Font: (Default) Times New Roman

Formatted: Font: (Default) Times New Roman

Formatted: Font: (Default) Times New Roman, English (Australia)

~~that~~ serve alcoholic beverage due to the high demand from tourists and locals [82~~]~~, ~~[~~83]. ~~Coastal areas typically have warmer temperatures and more sunshine, increasing thirst and making people more likely to consume alcohol.~~ Another characteristic of this area is the fishing and maritime culture. This culture is often associated with hard work and long working hours, and alcohol may be seen as a way to relax and unwind after a tough day at the sea [84]. Finally, this region has 68% urban and 32% rural zones [60]. The level of development, as measured by gross domestic product (GDP), is the third region with significant economic development in the country [85] (S3 **Table~~ S3~~**, **Supplementary Material**)~~(Kindly see Table S5)~~. Interestingly, the consumption of illegal drugs is lower in the Northern region than in other regions of the country. However, there is a more representative consumption of non-prescription tranquilizers, opioids, ketamine, GHB, and heroin. In particular, ~~T~~the Atlántico department has the highest consumption proportion within this region (**Fig 8**).

~~Likewise, t~~Tobacco consumption is present in all regions, with a higher proportion in the Central region, where climate conditions resemble temperate weather. Also, this region has a diverse consumption pattern, where drugs such as marijuana, popper, cocaine, ecstasy, inhalants, methadone, heroin, LSD, GHB, 2CB, and mushrooms prevail. This region has Colombia’~~'~~s largest ~~big~~ cities~~,~~ (i.e., ~~such as~~ Bogota~~á~~ and Medellin); ~~.~~Bogotá has ~~a~~the highest~~ high~~ population density and is a hub for drug trafficking routes~~;~~, while Medellín has an unfortunate history of drug cartels and gang violence. Ultimately, this region ~~has~~is comprised of 77% urban ~~and 23% rural zones~~areas, and the most developed cities in the country are located there~~ are located~~ [60~~]~~, ~~[~~85] (S3 **Table~~ S3~~**, **Supplementary Material**).~~(Kindly see Table S5).Likewise, tobacco is present in all regions with a higher proportion in the Central region where climate conditions resemble temperate weather. As for the Central region, there is a more diverse consumption pattern, where the use of drugs such as marijuana, popper, cocaine, ecstasy, inhalants, methadone, heroin, LSD, GHB, 2CB, and mushrooms prevails.~~

Formatted: Font: (Default) Times New Roman

Formatted: Font: (Default) Times New Roman

Formatted: Font: (Default) Times New Roman, English (Australia)

~~Similarly, e~~Energy drinks are more frequently used in the Eastern region, ~~which is~~ characterized by a continental climate surrounded by flat territory. ~~These~~ Our results are in line with the scientific literature suggesting that the location of regions within countries is directly associated with the consumption of ~~psychoactive substances~~PAS [26~~]~~, [86~~]–[~~88]. The consumption of heroin, basuco, non-prescription tranquilizers, stimulants, methamphetamines, opioids, and ketamine characterizes this region. This zone is the second most developed region in the country, and ~~it has a high percentage~~72% of urban areas ~~(72%), with the rest corresponding to rural areas (38%)~~ [60], [85] (~~**Kindly see Table S5**~~**S3 Table ~~S3~~, Supplementary Material**).

Our findings also show that the Southern region is more likely to consume illegal drugs, ~~such~~ including ~~as bazuco~~basuco, heroin, and Yagé (**Fig~~ure~~ 8**). One of the main reasons for this result is that, unfortunately, this region has ~~favourable~~favourable environmental characteristics (i.e., majority rainforest) for their consumption and production, being the second largest illegal drug-producing region in Colombia [79]~~.~~. Furthermore, this region has the highest percentage of rurality (52%) compared to the other regions, and its level of development is low ~~given that it generates the leas~~as measured by the~~t~~ GDP [60, 85] (S3 **Table ~~S3~~, Supplementary Material**) ~~(Kindly see Table S5)~~.

In the Western region, also known as the Pacific region, consumption mostly mainly includes of Mmethylene Cchloride (DICK), GHB, heroin, opioids, and methamphetamines. This region (Pacific) is mainly characterizedknown for its geographical isolation, poverty, and ongoing conflict, which have contributed to the growth of drug production and trafficking in the area. Poverty is one of the main factors driving drug production in the Pacific region, which has led many people to turn to drug cultivation and trafficking for survival. Additionally, the region''s rugged terrain and limited infrastructure have made it difficult for the Colombian government to establish a

Formatted: Font color: Text 1, English (Australia), Not Expanded by / Condensed by

Formatted: Font color: Text 1, English (Australia), Not Expanded by / Condensed by

Formatted: Font: Bold, Font color: Blue

Formatted: Font: Bold, Font color: Blue

Formatted: Font: (Default) Times New Roman

Formatted: Font: (Default) Times New Roman

Formatted: Font: (Default) Times New Roman, English (Australia)

strong presence, allowing drug traffickers to operate with relative impunity [89]. This region has a similar similar percentage of urban (53%) and rural (47%) populations than the Southern region, and . On the other hand, this region ranks second among the regions with the lowest levels of development (S3 **Table S3**, **Supplementary Material**).(Kindly see Table S5).

In the Western region, also known as the Pacific region, consumption mostly mainly includes of Mmethylene Cchloride (DICK), GHB, heroin, opioids, and methamphetamines. This region (Pacific) is mainly characterizedknown for its geographical isolation, poverty, and ongoing conflict, which have contributed to the growth of drug production and trafficking in the area. Poverty is one of the main factors driving drug production in the Pacific region, which has led many people to turn to drug cultivation and trafficking for survival. Additionally, the region''s rugged terrain and limited infrastructure have made it difficult for the Colombian government to establish a strong presence, allowing drug traffickers to operate with relative impunity [89]. This region has a similar similar percentage of urban (53%) and rural (47%) populations than the Southern region, and . On the other hand, this region ranks second among the regions with the lowest levels of development (S3 **Table S3**, **Supplementary Material**).(Kindly see Table S5). .,and S3 **S3**

As for the Central region, there is a more diverse consumption pattern, where the use of drugs such as marijuana, popper, cocaine, ecstasy, inhalants, methadone, heroin, LSD, GHB, 2CB, and mushrooms prevails.

The Eastern region is characterized by consumption of drugs such as heroin, bazuco, non-prescription tranquilizers, stimulants, methamphetamines, opioids, and ketamine.

Interestingly, consumption of illegal drugs is lower in the Northern region compared to other regions of the country. However, there is a more representative consumption of drugs such as non-prescription tranquilizers, opioids, ketamine, GHB, and heroine, with the Atlántico department having the highest proportion of consumption within this region (**Figure 8**).

Formatted: Font: Bold, Font color: Blue

Formatted: Font: (Default) Times New Roman

Formatted: Font: (Default) Times New Roman

Formatted: Font: (Default) Times New Roman, English (Australia)

Conclusion

In summary, the proposed CAE-DEC model simultaneously integrates a feature extraction process within the clustering design, prioritizing features that improve the separation between groups, thus avoiding the manual extraction of features, which is a frequent process in traditional models. Additionally, a geospatial component is sequentially included to expand the resulting insights by considering geographic constraints. Currently, these types of architectures are scarce in ~~the~~ understanding ~~of~~ mental health problems. As part of future work, the architecture of the proposed model could be improved to integrate the automatic extraction of features while optimizing a geospatial loss. Following our experience with the proposed CAE-DEC in ~~psychoactive substance~~ PAS consumption, the application of this model to other mental health problems, such as suicide, depression, and domestic violence, among other pathologies, ~~should~~ could be ~~timely~~ explored. Based on these results, effective interventions and/or government policies to prevent and/or mitigate their impact could be promoted and evaluated, for example, by developing regional interventions based on the types of drugs most prevalent in the area and the cultural and socio-economic characteristics. This can include education, treatment, and harm reduction programs. Also, this information can be used to develop public health campaigns to raise awareness about the risks of drug use and reduce their negative impact. Furthermore, this information can be used to crack down on drug trafficking and distribution networks. On the other hand, this information can be used to alert healthcare providers and regulatory bodies to take appropriate action to prevent their use and discover new drugs.~~Based on these results, effective interventions and/or government policies to prevent and/or mitigate their impact could be promoted and evaluated.~~

Acknowledgements. K.P. is a doctoral student at Universidad del Norte, Barranquilla, Colombia, and received a Ph.D. scholarship from this institution. Some of this work is to be presented to the Ph.D. program in partial fulfillment of the requirements for the Ph.D. degree. The funders had no role in study design, data collection and analysis, decision to publish, or preparation of the manuscript~~The sponsor of the study has no~~

Formatted: Font: 18 pt, Bold

Formatted: Indent: First line: 0"

Formatted: Font color: Text 1

Formatted: Font color: Blue

Formatted: Font: (Default) Times New Roman

Formatted: Font: (Default) Times New Roman

Formatted: Font: (Default) Times New Roman, English (Australia)

role in study design; in the collection, analysis, and interpretation of data; in the writing of the report; and in the decision to submit the article for publication.

Authors' Contributors. All authors contributed to the design of the study. K.P. was responsible for data extraction and analysis; JIV and CB provided important insight and supervised the analysis. All authors reviewed the manuscript.

Declaration of Competing Interest. None to declare.

Formatted: Font: (Default) Times New Roman

Formatted: Font: (Default) Times New Roman

Formatted: Font: (Default) Times New Roman, English (Australia)

FIGURE LEGENDS

Figure 1. Architecture of the proposed CAE-DEC model.

Figure 2.: Derived Latent Feature Space based on the **(a)** CAE and **(b)** CAE-DEC models.

Figure 3. Resulting clusters for individuals **(a)** consuming and **(b)** not consuming psychoactive substances based on the CAE-DEC model.

Figure 4. Absolute deviation around class medians (ADCM) statistic criterion for different alternative classifiers. Here, lower is better.

Figure 5. (a) Consumption percentage of psychoactive substances according to the CAE-DEC model; **(b)** Moran's I statistic; **(c)** Moran's cluster map. Here, HH, LH, LL and ns represent high-high, low-high, low-low and not statistically significant quadrants, respectively. This clustering pattern leads to a statistically significant Moran's I statistic of 0.2 (*P*-value <0.01).

Figure 6. Frequency of consumption of **(a)** legal and **(b)** illegal drugs in Colombia.

Figure 7. Consumption of illegal drugs by department. For interpretation purposes, number represents values scaled on a range of 0 to 1. For instance, Bogotá D.C. has the highest LSD consumption and Putumayo has the lowest.

Figure 8. Consumption of legal drugs by department. Number represents values scaled on a range of 0 to 1 for psychoactive substance use. Conventions as in **Figure Fig** 7.

Figure 9. Cluster map of drug use after regionalization.

Formatted: Font color: Blue

Formatted: Font color: Blue

Formatted: Font color: Blue

Formatted: Font color: Blue

Formatted: Font color: Blue

Formatted: Font color: Blue

Formatted: Font color: Blue

Formatted: Font color: Blue

Formatted: Font: Bold, Font color: Blue

Formatted: Font color: Blue

Formatted: Font: (Default) Times New Roman

Formatted: Font: (Default) Times New Roman

Formatted: Font: (Default) Times New Roman, English (Australia)

TABLES

Table 1. Pseudo code for the CAE-DEC training process.

Pseudo code: The CAE-DEC training process
Input data: Number of clusters n; Normalized input data x; Maximum number of iterations Mint; Balance coefficient C; Pretrained CAE; Stop condition Stop; Target distribution P update condition P_change.
Training process:
1. Generate an initial latent space (Z) through the pre-trained CAE
2. Run spectral clustering with Z to generate the initial cluster centers (C)
3. Initialize the CAE-DEC model with the pretrained CAE.
4. Calculate soft assignment distribution Q and target distribution P based on *Z* and *C*
for epoch<Minter do:
if epoch%P_change==0 then:
Calculate soft assignment distribution Q and target distribution P based on Z and *C*
end if
Feed the CAE-DEC with the normalized input data x
Calculate the reconstruction loss and the clustering loss
Update CAE-DEC parameters. Weight, Bias, and Centers.
if Stop==True then:
Break
end if
end for
Obtain the label for each data point from the las optimized Q.
Output: Latent space, labels

Formatted: Font color: Blue

Formatted: Font color: Blue

Formatted: Font: (Default) Times New Roman

Formatted: Font: (Default) Times New Roman

Formatted: Font: (Default) Times New Roman, English (Australia)

Table 2. Distribution of demographic and social variables across clusters.

Variables		Cluster 0 (*n*=14935)	Cluster 1 (*n*=11528)	Cluster 2 (*n*=23005)	χ^2	*df*	*P*-value
Region	Caribbean	3004	2991	4075	1472.9	10	<.0001
	Central-Eastern	2526	2299	6175			
	Central-Southern	1843	1299	1702			
	Eje Cafetero – Antioquia	3902	2354	6371			
	Llanos Orientales	1687	1305	1496			
	Pacific	1973	1280	3186			
Gender	Male	6606	3927	10233	386.72	2	<.0001
	Female	8329	7601	12772			
Housing type	House	8250	6538	11834	114.18	6	<.0001
	Apartment	6275	4724	10595			
	Room	395	256	543			
	Indigenous dwelling	15	10	33			
Socioeconomic status	1	3889	3626	6945	843.92	10	<.0001
	2	4627	3902	8851			
	3	4284	2867	5683			
	4	1341	718	979			
	5	510	256	362			
	6	284	159	185			
Age (years)	(0, 20]	2105	1576	2998	345.87	4	<.0001
	(20, 40]	6814	4329	10889			
	(40, 68]	6016	5623	9118			
Contribute to the household finances	Yes	10128	7473	16041	85.24	2	<.0001
	No	4807	4055	6964			

df: Degrees of freedom.

Formatted: Font color: Blue

Formatted: Font color: Blue

Formatted: Font: (Default) Times New Roman

Formatted: Font: (Default) Times New Roman

Formatted: Font: (Default) Times New Roman, English (Australia)

Table 3. Adjusted residuals comparing the observed and expected frequencies based on the cluster analysis.

	**Variables**	**Cluster 0 (*n*=14935)**	**Cluster 1 (*n*=11528)**	**Cluster 2 (*n*=23005)**
Region	Caribbean	-0.88	17.02	-13.61
	Central-Eastern	-18.72	-6.76	22.97
	Central-Southern	12.54	6.09	-16.7
	Eje Cafetero - Antioquia	2.02	-14.36	10.31
	Llanos Orientales	11.32	9.59	-18.55
	Pacific	0.84	-6.97	5.13
Gender	Male	6.68	-19.66	10.52
	Female	-6.68	19.66	-10.52
Housing type	House	4.17	7.13	-9.88
	Apartment	-4.83	-6.61	10.05
	Room	2.2	-1.54	-0.72
	Indigenous dwelling	-0.72	-1.09	1.59
Socioeconomic status	1	-10.26	5.99	4.37
	2	-12.72	-3.3	14.51
	3	9.14	-3	-5.87
	4	17.29	0.44	-16.29
	5	11.12	-0.49	-9.82
	6	8.26	1.2	-8.62
Age (years)	(0, 20]	2.54	0.61	-2.85
	(20, 40]	3.2	-17.23	11.66
	(40, 68]	-4.98	16.93	-9.77
Contribute to the household finances	Yes	-0.61	-8.37	7.65
	No	0.61	8.37	-7.65

Formatted: Font color: Blue

Formatted: Font color: Blue

Formatted: Font: (Default) Times New Roman

Formatted: Font: (Default) Times New Roman

Formatted: Font: (Default) Times New Roman, English (Australia)

References

[1] OPS, "Abuso de sustancias ," *Organización Panamericana de la Salud*, 2022. https://www.paho.org/es/temas/abuso-sustancias (accessed Jan. 25, 2022).

[2] C. Heesun, L. Jaesin, and K. Eunmi, "Trends of novel psychoactive substances (NPSs) and their fatal cases," *Forensic Toxicol.*, vol. 34, no. 1, pp. 1–11, 2016, Accessed: Jan. 20, 2022. [Online]. Available: https://jglobal.jst.go.jp/en/detail?JGLOBAL_ID=201602283742633549

[3] A. L. Riley *et al.*, "Abuse potential and toxicity of the synthetic cathinones (i.e., 'Bath salts')," *Neurosci. Biobehav. Rev.*, vol. 110, pp. 150–173, Mar. 2020, doi: 10.1016/J.NEUBIOREV.2018.07.015.

[4] S. Assi, N. Gulyamova, P. Kneller, and D. Osselton, "The effects and toxicity of cathinones from the users' perspectives: A qualitative study," *Hum. Psychopharmacol. Clin. Exp.*, vol. 32, no. 3, p. e2610, May 2017, doi: 10.1002/HUP.2610.

[5] V. Lukić, R. Micić, B. Arsić, B. Nedović, and Ž. Radosavljević, "Overview of the major classes of new psychoactive substances, psychoactive effects, analytical determination and conformational analysis of selected illegal drugs," *Open Chem.*, vol. 19, no. 1, pp. 60–106, Jan. 2021, doi: 10.1515/CHEM-2021-0196/XML.

[6] N. Uchiyama, S. Matsuda, M. Kawamura, R. Kikura-Hanajiri, and Y. Goda, "Two new-type cannabimimetic quinolinyl carboxylates, QUPIC and QUCHIC, two new cannabimimetic carboxamide derivatives, ADB-FUBINACA and ADBICA, and five synthetic cannabinoids detected with a thiophene derivative α-PVT and an opioid receptor agonist AH-7921 identified in illegal products," *Forensic Toxicol. 2013 312*, vol. 31, no. 2, pp. 223–240, Mar. 2013, doi: 10.1007/S11419-013-0182-9.

[7] B. F. Grant *et al.*, "Prevalence and co-occurrence of substance use disorders and independent mood and anxiety disorders - Results from the national epidemiologic survey on alcohol and related conditions," *Arch. Gen. Psychiatry*, vol. 61, no. 8, pp. 807–816, 2004, doi: 10.1001/archpsyc.61.8.807.

Formatted: Font: 18 pt

Field Code Changed

Formatted: Font: (Default) Times New Roman

Formatted: Font: (Default) Times New Roman

Formatted: Font: (Default) Times New Roman, English (Australia)

[8] CDC, "Understanding the Epidemic," 2020. https://www.cdc.gov/opioids/basics/epidemic.html (accessed Jan. 21, 2022).

[9] R. Z. Goetzel, K. Hawkins, R. J. Ozminkowski, and S. H. Wang, "The health and productivity cost burden of the 'top 10' physical and mental health conditions affecting six large US employers in 1999," *J. Occup. Environ. Med.*, vol. 45, no. 1, pp. 5–14, 2003, doi: 10.1097/00043764-200301000-00007.

[10] W. F. Stewart, J. A. Ricci, E. Chee, S. R. Hahn, and D. Morganstein, "Cost of lost productive work time among US workers with depression," *JAMA-JOURNAL Am. Med. Assoc.*, vol. 289, no. 23, pp. 3135–3144, Jun. 2003, doi: 10.1001/jama.289.23.3135.

[11] F. L. G. Garcia and J. C. A. Murillo, "The United Nations and 21st century security challenges in Colombia," *Rev. Cient. Gen. Jose Maria Cordova*, vol. 19, no. 36, pp. 929–940, 2021, doi: 10.21830/19006586.875.

[12] J. P. Aschner and J. C. Montero, "Architectures, spaces, and territories of illicit drug trafficking in Colombia and Mexico:," *https://doi.org/10.1177/1741659020910212*, vol. 17, no. 3, pp. 327–351, Mar. 2020, doi: 10.1177/1741659020910212.

[13] ODC, "Observatorio de drogas de Colombia," 2022. https://www.minjusticia.gov.co/programas-co/ODC/Paginas/SIDCO-departamento-municipio.aspx (accessed Jun. 09, 2022).

[14] DANE, "Encuesta Nacional de Consumo de Sustancias Psicoactivas," 2020. Accessed: Apr. 23, 2021. [Online]. Available: https://www.dane.gov.co/files/investigaciones/boletines/encspa/comunicado-encspa-2019.pdf

[15] DANE, "Estudio nacional de consumo de sustancias psicoactivas en Colombia," Bogotá, 2014. Accessed: Jan. 17, 2022. [Online]. Available: https://www.unodc.org/documents/colombia/2014/Julio/Estudio_de_Consumo_UNODC.pdf

[16] UNODC, "Drogas sintéticas y nuevas sustancias psicoactivas en América Latina y el Caribe

Formatted: Font: (Default) Times New Roman
Formatted: Font: (Default) Times New Roman
Formatted: Font: (Default) Times New Roman, English (Australia)

2021," Viena, 2021. Accessed: Jan. 21, 2022. [Online]. Available: https://www.minjusticia.gov.co/programas-co/ODC/Documents/Publicaciones/GlobalSmartLA (1).pdf?csf=1&e=MH9EHg

[17] P. Griffiths and R. Mcketin, "Developing a global perspective on drug consumption patterns and trends-the challenge for drug epidemiology," *Bull. Narcotics*, vol. 5, no. 1, 2003.

[18] W. A. Lanier, E. M. Johnson, R. T. Rolfs, M. D. Friedrichs, and T. C. Grey, "Risk factors for prescription opioid-related death, Utah, 2008-2009," *Pain Med.*, vol. 13, no. 12, pp. 1580–1589, 2012, doi: 10.1111/J.1526-4637.2012.01518.X.

[19] S. S. Martins, L. Sampson, M. Cerdá, and S. Galea, "Worldwide Prevalence and Trends in Unintentional Drug Overdose: A Systematic Review of the Literature," *Am. J. Public Health*, vol. 105, no. 11, pp. e29–e49, Nov. 2015, doi: 10.2105/AJPH.2015.302843.

[20] CDC, "Today's Heroin Epidemic ," *Centers for Disease Control and Prevention*, 2015. https://www.cdc.gov/vitalsigns/heroin/index.html (accessed Jan. 16, 2022).

[21] C. M. Fuller *et al.*, "Effects of race, neighborhood, and social network on age at initiation of injection drug use," *Am. J. Public Health*, vol. 95, no. 4, pp. 689–695, Apr. 2005, doi: 10.2105/AJPH.2003.02178.

[22] P. J. Fite, P. Wynn, J. E. Lochman, and K. C. Wells, "The Influence of Neighborhood Disadvantage and Perceived Disapproval on Early Substance Use Initiation," *Addict. Behav.*, vol. 34, no. 9, p. 769, Sep. 2009, doi: 10.1016/J.ADDBEH.2009.05.002.

[23] S. R. Friedman *et al.*, "Income inequality, drug-related arrests, and the health of people who inject drugs: Reflections on seventeen years of research," *Int. J. Drug Policy*, vol. 32, pp. 11–16, Jun. 2016, doi: 10.1016/J.DRUGPO.2016.03.003.

[24] M. Jensen, L. Chassin, and N. A. Gonzales, "Neighborhood Moderation of Sensation Seeking Effects on Adolescent Substance Use Initiation," *J. Youth Adolesc.*, vol. 46, no. 9, p. 1953, Sep. 2017, doi: 10.1007/S10964-017-0647-Y.

[25] C. Sarah and J. Leonard A, "Contextual Perspectives on Heroin Addiction and Recovery:

Formatted: Font: (Default) Times New Roman

Formatted: Font: (Default) Times New Roman

Formatted: Font: (Default) Times New Roman, English (Australia)

Classic and Contemporary Theories," *Int. Arch. Public Heal. Community Med.*, vol. 2, no. 1, Dec. 2018, doi: 10.23937/IAPHCM-2017/1710009.

[26] P. Bozorgi, D. E. Porter, J. M. Eberth, J. P. Eidson, and A. Karami, "The leading neighborhood-level predictors of drug overdose: A mixed machine learning and spatial approach," *Drug Alcohol Depend.*, vol. 229, p. 109143, Dec. 2021, doi: 10.1016/J.DRUGALCDEP.2021.109143.

[27] S. Galea, S. Rudenstine, and D. Vlahov, "Drug use, misuse, and the urban environment," *Drug Alcohol Rev.*, vol. 24, no. 2, pp. 127–136, Mar. 2005, doi: 10.1080/09595230500102509.

[28] C. A. Latkin, V. Forman, A. Knowlton, and S. Sherman, "Norms, social networks, and HIV-related risk behaviors among urban disadvantaged drug users," *Soc. Sci. Med.*, vol. 56, no. 3, pp. 465–476, Feb. 2003, doi: 10.1016/S0277-9536(02)00047-3.

[29] J. R. Schroeder, C. A. Latkin, D. R. Hoover, A. D. Curry, A. R. Knowlton, and D. D. Celentano, "Illicit drug use in one's social network and in one's neighborhood predicts individual heroin and cocaine use," *Ann. Epidemiol.*, vol. 11, no. 6, pp. 389–394, 2001, doi: 10.1016/S1047-2797(01)00225-3.

[30] A. Campo-Arias, Y. P. Suárez-Colorado, and C. C. Caballero- Domínguez, "Asociación entre el consumo de Cannabis y el riesgo de suicidio en adolescentes escolarizados de Santa Marta, Colombia," *Biomédica*, vol. 40, no. 3, p. 569, 2020, doi: 10.7705/BIOMEDICA.4988.

[31] A. L. Fajardo, "Consumption of psychopharmaceuticals in the city of Bogota (Colombia): a new reality," *Arch. Med.*, vol. 18, no. 2, 2018, doi: 10.30554/archmed.18.2.2743.2018.

[32] O. Scoppetta and G. A. Castaño, "Early drug consumption and subsequent risk of illicit drug use in Colombia," *Addict. Disord. their Treat.*, vol. 18, no. 1, pp. 10–14, Mar. 2019, doi: 10.1097/ADT.0000000000000144.

[33] C. Scheuer *et al.*, "El consumo de sustancias psicoactivas en jóvenes estudiantes de una institución educativa del municipio de Neira (Caldas): un estudio de caso desde la mirada de

la educación inclusiva," *Cult. y Drog.*, vol. 23, no. 26, pp. 343–354, Jul. 2018, doi: 10.2/JQUERY.MIN.JS.

[34] J. Kalyanam, T. Katsuki, G. R.G. Lanckriet, and T. K. Mackey, "Exploring trends of nonmedical use of prescription drugs and polydrug abuse in the Twittersphere using unsupervised machine learning," *Addict. Behav.*, vol. 65, pp. 289–295, Feb. 2017, doi: 10.1016/j.addbeh.2016.08.019.

[35] M. A. Narvaez-Chicaiza, "Harm Reduction Policies Where Drugs Constitute a Security Issue," *Heal. Care Anal.*, vol. 28, no. 4, pp. 382–390, Dec. 2020, doi: 10.1007/S10728-020-00415-9.

[36] S. M. Restrepo-Escobar and E. A. S. Cardona, "Educational and prevention campaigns. A review on the use of psychoactive substances in Colombian university students," *Interdisciplinaria*, vol. 38, no. 2, pp. 199–208, 2021, doi: 10.16888/INTERD.2021.38.2.13.

[37] E. Hancer, B. Xue, and M. Zhang, "A survey on feature selection approaches for clustering," *Artif. Intell. Rev. 2020 536*, vol. 53, no. 6, pp. 4519–4545, Jan. 2020, doi: 10.1007/S10462-019-09800-W.

[38] T. D. Wager, L. Y. Atlas, M. A. Lindquist, M. Roy, C.-W. Woo, and E. Kross, "An fMRI-Based Neurologic Signature of Physical Pain," *N. Engl. J. Med.*, vol. 368, no. 15, pp. 1388–1397, 2013, doi: 10.1056/NEJMoa1204471.

[39] A. Henriksson, M. Kvist, H. Dalianis, and M. Duneld, "Identifying adverse drug event information in clinical notes with distributional semantic representations of context," *J. Biomed. Inform.*, vol. 57, pp. 333–349, Oct. 2015, doi: 10.1016/j.jbi.2015.08.013.

[40] L. M. Squeglia *et al.*, "Neural Predictors of Initiating Alcohol Use During Adolescence," *Am. J. Psychiatry*, vol. 174, no. 2, pp. 172–185, Feb. 2017, doi: 10.1176/appi.ajp.2016.15121587.

[41] M. Conway and D. O'Connor, "Social media, big data, and mental health: current advances and ethical implications," *Curr. Opin. Psychol.*, vol. 9, pp. 77–82, Jun. 2016, doi: 10.1016/j.copsyc.2016.01.004.

Formatted: Font: (Default) Times New Roman

Formatted: Font: (Default) Times New Roman

Formatted: Font: (Default) Times New Roman, English (Australia)

[42] T. Katsuki, T. K. Mackey, and R. Cuomo, “Establishing a Link Between Prescription Drug Abuse and Illicit Online Pharmacies: Analysis of Twitter Data,” *J. Med. INTERNET Res.*, vol. 17, no. 12, 2015, doi: 10.2196/jmir.5144.

[43] L. Degenhardt *et al.*, “The global epidemiology and burden of psychostimulant dependence: Findings from the Global Burden of Disease Study 2010,” *Drug Alcohol Depend.*, vol. 137, pp. 36–47, 2014, doi: 10.1016/j.drugalcdep.2013.12.025.

[44] H. A. Whiteford *et al.*, “Global burden of disease attributable to mental and substance use disorders: findings from the Global Burden of Disease Study 2010,” *Lancet*, vol. 382, no. 9904, pp. 1575–1586, Nov. 2013, doi: 10.1016/S0140-6736(13)61611-6.

[45] F. D. Bowman, B. Caffo, S. S. Bassett, and C. Kilts, “A Bayesian hierarchical framework for spatial modeling of fMRI data,” *Neuroimage*, vol. 39, no. 1, pp. 146–156, 2008, doi: 10.1016/j.neuroimage.2007.08.012.

[46] K. Shannon, M. Rusch, J. Shoveller, D. Alexson, K. Gibson, and M. W. Tyndall, “Mapping violence and policing as an environmental-structural barrier to health service and syringe availability among substance-using women in street-level sex work,” *Int. J. DRUG POLICY*, vol. 19, no. 2, pp. 140–147, 2008, doi: 10.1016/j.drugpo.2007.11.024.

[47] B. Freisthler, B. Needell, and P. J. Gruenewald, “Is the physical availability of alcohol and illicit drugs related to neighborhood rates of child maltreatment?,” *Child Abuse Negl.*, vol. 29, no. 9, pp. 1049–1060, Sep. 2005, doi: 10.1016/j.chiabu.2004.12.014.

[48] J. K. Bass and S. F. Lambert, “Urban adolescents’ perceptions of their neighborhoods: An examination of spatial dependence,” *J. Community Psychol.*, vol. 32, no. 3, pp. 277–293, May 2004, doi: 10.1002/jcop.20005.

[49] B. Chaix *et al.*, “Spatial clustering of mental disorders and associated characteristics of the neighbourhood context in Malmo, Sweden, in 2001,” *J. Epidemiol. Community Health*, vol. 60, no. 5, pp. 427–435, May 2006, doi: 10.1136/jech.2005.040360.

[50] C. T. Mowbray, M. C. Holter, G. B. Teague, and D. Bybee, “Fidelity criteria: Development,

Formatted: Font: (Default) Times New Roman

Formatted: Font: (Default) Times New Roman

Formatted: Font: (Default) Times New Roman, English (Australia)

measurement, and validation,” *Am. J. Eval.*, vol. 24, no. 3, pp. 315–340, 2003, doi: 10.1177/109821400302400303.

[51] M. Peet and C. Stokes, “Omega-3 fatty acids in the treatment of psychiatric disorders,” *Drugs*, vol. 65, no. 8, pp. 1051–1059, 2005, doi: 10.2165/00003495-200565080-00002.

[52] K. Chichester *et al.*, “Pharmacies and features of the built environment associated with opioid overdose: A geospatial comparison of rural and urban regions in Alabama, USA,” *Int. J. Drug Policy*, vol. 79, May 2020, doi: 10.1016/J.DRUGPO.2020.102736.

[53] P. Geissert *et al.*, “High-risk prescribing and opioid overdose: prospects for prescription drug monitoring program-based proactive alerts,” *Pain*, vol. 159, no. 1, pp. 150–156, Jan. 2018, doi: 10.1097/J.PAIN.0000000000001078.

[54] C. Fraley and A. E. Raftery, “How many clusters? Which clustering method? Answers via model-based cluster analysis,” *Comput. J.*, vol. 41, no. 8, pp. 586–588, 1998, doi: 10.1093/COMJNL/41.8.578.

[55] A. Saxena *et al.*, “A review of clustering techniques and developments,” *Neurocomputing*, vol. 267, pp. 664–681, Dec. 2017, doi: 10.1016/J.NEUCOM.2017.06.053.

[56] B. Li, D. Pi, Y. Lin, and L. Cui, “DNC: A Deep Neural Network-based Clustering-oriented Network Embedding Algorithm,” *J. Netw. Comput. Appl.*, vol. 173, Jan. 2021, doi: 10.1016/J.JNCA.2020.102854.

[57] J. Xie, R. Girshick, and A. Farhadi, “Unsupervised Deep Embedding for Clustering Analysis,” *33rd Int. Conf. Mach. Learn. ICML 2016*, vol. 1, pp. 740–749, Nov. 2016, Accessed: Jan. 15, 2022. [Online]. Available: https://arxiv.org/abs/1511.06335v2

[58] S. Sharifipour, H. Fayyazi, and M. Sabokro, “Unsupervised Feature Selection using Encoder-Decoder Networks,” *6th Iran. Conf. Signal Process. Intell. Syst. ICSPIS 2020*, Dec. 2020, doi: 10.1109/ICSPIS51611.2020.9349608.

[59] B. Li, D. Pi, Y. Lin, and L. Cui, “DNC: A Deep Neural Network-based Clustering-oriented Network Embedding Algorithm,” *J. Netw. Comput. Appl.*, vol. 173, no. May 2020, p.

Formatted: Font: (Default) Times New Roman

Formatted: Font: (Default) Times New Roman

Formatted: Font: (Default) Times New Roman, English (Australia)

102854, 2021, doi: 10.1016/j.jnca.2020.102854.

[60] DANE, “Departamento Administrativo Nacional de Estadística. Censo Nacional de Población y Vivienda 2018. Proyecciones de Población 2018-2020, total municipal por área Junio 30.” Bogotá D.C, Colombia, 2018.

[61] DNP, “Avances y complementariedades estratégicas de los Distritos en el marco de los esquemas asociativos territoriales,” Bogotá D.C, 2018. [Online]. Available: https://colaboracion.dnp.gov.co/CDT/Desarrollo Territorial/Conversatorio Distrito Cali 04_10_2018 - Santiago Arroyo.pdf

[62] UNODC, “Monitoreo de territorios afectados por cultivos ilícitos 2020,” Bogotá, 2021. Accessed: Jan. 14, 2022. [Online]. Available: https://www.unodc.org/documents/crop-monitoring/Colombia/Colombia_Monitoreo_de_territorios_afectados_por_cultivos_ilicitos_2020.pdf

[63] ODC, “Estudio nacional de consumo de sustancias psicoactivas,” Bogotá, 2019. Accessed: Jan. 14, 2022. [Online]. Available: https://www.odc.gov.co/Portals/1/publicaciones/pdf/estudio Nacional de consumo 2019.pdf

[64] DANE, “Encuesta Nacional de Consumo de Sustancias Psicoactivas en Población General 2019,” *2020*. https://microdatos.dane.gov.co/index.php/catalog/680/get_microdata (accessed Jan. 14, 2022).

[65] J. Espinosa, “Shapefile,” 2022. https://hub.arcgis.com/datasets/de0e829ddbf743c895ba6dcee1b74fae/about (accessed Jun. 09, 2022).

[66] L. Anselin, *Spatial Econometrics: Methods and Models*, Springer Netherlands. 1988. doi: 10.1007/978-94-015-7799-1_1.

[67] P. Moran, *The Interpretation of Statistical Maps* , 2nd ed., vol. 10. Journal of the Royal Statistical Society, 1948. Accessed: Jan. 23, 2022. [Online]. Available: https://www.jstor.org/stable/2983777

Formatted: Font: (Default) Times New Roman

Formatted: Font: (Default) Times New Roman

Formatted: Font: (Default) Times New Roman, English (Australia)

[68] R. C. Geary, “The Contiguity Ratio and Statistical Mapping,” *Inc. Stat.*, vol. 5, no. 3, p. 115, Nov. 1954, doi: 10.2307/2986645.

[69] A. Getis and J. K. Ord, “The Analysis of Spatial Association by Use of Distance Statistics,” *Geogr. Anal.*, vol. 24, no. 3, pp. 189–206, Jul. 1992, doi: 10.1111/J.1538-4632.1992.TB00261.X.

[70] L. Anselin, “Local Indicators of Spatial Association—LISA,” *Geogr. Anal.*, vol. 27, no. 2, pp. 93–115, Apr. 1995, doi: 10.1111/J.1538-4632.1995.TB00338.X.

[71] J. C. Duque, R. Ramos, and J. Suriñach, “Supervised Regionalization Methods: A Survey:,” *http://dx.doi.org/10.1177/0160017607301605*, vol. 30, no. 3, pp. 195–220, Jul. 2016, doi: 10.1177/0160017607301605.

[72] S. Rey, D. Arribas-Bel, and L. Wolf, *Geographic Data Science with Python*. 2020. Accessed: Jan. 23, 2022. [Online]. Available: https://geographicdata.science/book/intro.html

[73] G. Van Rossum and F. L. Drake, *Python 3 Reference Manual*. Scotts Valley, CA: CreateSpace, 2009.

[74] J. Masci, U. Meier, D. Cireşan, and J. Schmidhuber, “Stacked Convolutional Auto-Encoders for Hierarchical Feature Extraction,” *Lect. Notes Comput. Sci. (including Subser. Lect. Notes Artif. Intell. Lect. Notes Bioinformatics)*, vol. 6791 LNCS, no. PART 1, pp. 52–59, 2011, doi: 10.1007/978-3-642-21735-7_7.

[75] A. Shrestha and A. Mahmood, “Review of deep learning algorithms and architectures,” *IEEE Access*, vol. 7, pp. 53040–53065, 2019, doi: 10.1109/ACCESS.2019.2912200.

[76] T. Caliñski and J. Harabasz, “A Dendrite Method Foe Cluster Analysis,” *Commun. Stat.*, vol. 3, no. 1, pp. 1–27, 1974, doi: 10.1080/03610927408827101.

[77] D. L. Davies and D. W. Bouldin, “A Cluster Separation Measure,” *IEEE Trans. Pattern Anal. Mach. Intell.*, vol. PAMI-1, no. 2, pp. 224–227, 1979, doi: 10.1109/TPAMI.1979.4766909.

[78] P. J. Rousseeuw, “Silhouettes: A graphical aid to the interpretation and validation of cluster

Formatted: Font: (Default) Times New Roman

Formatted: Font: (Default) Times New Roman

Formatted: Font: (Default) Times New Roman, English (Australia)

analysis," *J. Comput. Appl. Math.*, vol. 20, no. C, pp. 53–65, Nov. 1987, doi: 10.1016/0377-0427(87)90125-7.

[79] ODC, "Density of drug production in Colombia," 2021. https://www.datos.gov.co/d/acs4-3wgp/visualization (accessed Jul. 05, 2022).

[80] H. Clarke, N. Soneji, D. T. Ko, L. Yun, and D. N. Wijeysundera, "Rates and risk factors for prolonged opioid use after major surgery: population based cohort study," *BMJ*, vol. 348, Feb. 2014, doi: 10.1136/BMJ.G1251.

[81] Y. F. Kuo, M. A. Raji, N. W. Chen, H. Hasan, and J. S. Goodwin, "Trends in Opioid Prescriptions Among Part D Medicare Recipients From 2007 to 2012," *Am. J. Med.*, vol. 129, no. 2, pp. 221.e21-221.e30, Feb. 2016, doi: 10.1016/J.AMJMED.2015.10.002.

[82] S. Puigcorbé *et al.*, "Assessing the association between tourism and the alcohol urban environment in Barcelona: a cross-sectional study," *BMJ Open*, vol. 10, no. 9, p. e037569, Sep. 2020, doi: 10.1136/BMJOPEN-2020-037569.

[83] M. Easwaran, J. Bazroy, V. Jayaseelan, and Z. Singh, "Prevalence and determinants of alcohol consumption among adult men in a coastal area of south India," *Int. J. Med. Sci. Public Heal.*, vol. 4, no. 3, p. 360, 2015, doi: 10.5455/IJMSPH.2015.1010201479.

[84] P. Chinnakali, P. Thekkur, A. Manoj Kumar, G. Ramaswamy, B. Bharadwaj, and G. Roy, "Alarmingly high level of alcohol use among fishermen: A community based survey from a coastal area of south India," *J. Forensic Leg. Med.*, vol. 42, pp. 41–44, Aug. 2016, doi: 10.1016/J.JFLM.2016.05.006.

[85] DANE, "Producto Interno Bruto por departamento," 2021.

[86] M. C. García *et al.*, "Opioid Prescribing Rates in Nonmetropolitan and Metropolitan Counties Among Primary Care Providers Using an Electronic Health Record System — United States, 2014–2017," *MMWR. Morb. Mortal. Wkly. Rep.*, vol. 68, no. 2, pp. 25–30, Jan. 2019, doi: 10.15585/MMWR.MM6802A1.

[87] K. M. Keyes, M. Cerdá, J. E. Brady, J. R. Havens, and S. Galea, "Understanding the Rural–

Formatted: Font: (Default) Times New Roman

Formatted: Font: (Default) Times New Roman

Formatted: Font: (Default) Times New Roman, English (Australia)

Urban Differences in Nonmedical Prescription Opioid Use and Abuse in the United States," *Am. J. Public Health*, vol. 104, no. 2, p. e52, Feb. 2014, doi: 10.2105/AJPH.2013.301709.

[88] N. B. King, V. Fraser, C. Boikos, R. Richardson, and S. Harper, "Determinants of Increased Opioid-Related Mortality in the United States and Canada, 1990–2013: A Systematic Review," *Am. J. Public Health*, vol. 104, no. 8, p. e32, 2014, doi: 10.2105/AJPH.2014.301966.

[89] UNODC, "Persistencia de los cultivos de coca en la Región Pacífica," 2010.

Supporting information

S1 Table. Feature coherence measurements

S2 Table. Model comparison

S3 Table. Characteristics of the level of development, urbanity, rurality, and drug production in the regions of Colombia.

S1~~3~~ Fig. The optimal number of clusters using Dendrogram.

S2~~4~~ Fig. Maps of Local Indicators of Spatial Association (LISA).

Formatted: Spanish (Spain)

Formatted: Font: 18 pt, Bold, Font color: Blue

Formatted: Font: 18 pt

Formatted: Font: Bold, Font color: Blue

Formatted: Font: (Default) Times New Roman

Formatted: Font: (Default) Times New Roman

Formatted: Font: (Default) Times New Roman, English (Australia)

UNIVERSIDAD DEL NORTE
Vigilada Mineducación

Barranquilla, March 31, 2023

Dr. Vinicius Silva Belo
Academic Editor, *PLOS ONE*

RE: Revised Manuscript # PONE-D-22-28262

Dear Dr. Silva,

Thank you for the opportunity to submit a revised version of our manuscript, "***Leading Consumption Patterns of Psychoactive Substances in Colombia: A Deep Neural Network-based Clustering-oriented Embedding Approach***," which now includes responses to the concerns, inquiries, comments, and suggestions raised by two anonymous reviewers. Please find below our response (in **blue**).

We very much appreciate all your efforts as Editor-in-Chief and the detailed and extraordinary revision this manuscript had. We enjoyed the thorough review of our manuscript, and it was a pleasure to respond to the reviewers. Please, Dr. Silva, allows us to mention that this exercise of the fair and exigent peer-review process is disappearing, and only good journals like *PLOS ONE* keep it. We hope that our new version might be suitable for publication in *PLOS ONE.*

Thank you very much for your time and consideration.

Yours sincerely,

Kevin R. Palomino, PhD(c)
Corresponding author

.

Comments from the Editor

1. Please ensure that your manuscript meets PLOS ONE's style requirements, including those for file naming. The PLOS ONE style templates can be found at https://journals.plos.org/plosone/s/file?id=wjVg/PLOSOne_formatting_sample_main_body.pdf and https://journals.plos.org/plosone/s/file?id=ba62/PLOSOne_formatting_sample_title_authors_affiliations.pdf

We very much appreciate your comments. We have updated our manuscript according to the journal's formatting. In particular,

- We changed the figure citation, i.e., from "Figure" to "Fig".
- We added the Numbered Equation.
- We added the supporting information captions.
- We changed the Cite references into brackets.

2. Please confirm that all data sources you used were publicly available and anonymized. Furthermore, please clarify how the data were accessed for the purpose of this study.

Thank you very much for your comments. The data is fully available and anonymized, and can be retrieved from https://microdatos.dane.gov.co/index.php/catalog/680/data-dictionary

For practicality, we downloaded the data and stored it on the cloud. The data can be retrieved from https://acortar.link/ktgCD9

3. In the ethics statement in the manuscript and in the online submission form, please provide additional information about the patient records/samples used in your retrospective study. Specifically, please ensure that you have discussed whether all data/samples were fully anonymized before you accessed them and/or whether the IRB or ethics committee waived the requirement for informed consent. If patients provided informed written consent to have data/samples from their medical records used in research, please include this information.

Thank you for your comment. As per Colombian regulations, the data were fully anonymized by the Colombian government before we could use them.

4. Thank you for stating the following financial disclosure:
"The author(s) received no specific funding for this work."

At this time, please address the following queries:

a) Please clarify the sources of funding (financial or material support) for your study. List the grants or organizations that supported your study, including funding received from your institution.

`

K.P. is a doctoral student at Universidad del Norte, Barranquilla, Colombia, and received a Ph.D. scholarship from this institution to cover tuition expenses. However, Universidad del Norte did not provide any additional funds for this study.

b) State what role the funders took in the study. If the funders had no role in your study, please state: “The funders had no role in study design, data collection and analysis, decision to publish, or preparation of the manuscript.”

Thank you for your comment. The following statement was included in the revised version of the manuscript to address this:

“The funders had no role in study design, data collection and analysis, decision to publish, or preparation of the manuscript”.

c) If any authors received a salary from any of your funders, please state which authors and which funders.

As stated previously, we did not receive any salary from organizations.

d) If you did not receive any funding for this study, please state: “The authors received no specific funding for this work.”

Done. We have added the following statement in the revised version of the manuscript:

“The authors received no specific funding for this work”.

Please include your amended statements within your cover letter; we will change the online submission form on your behalf.

4. Thank you for stating the following in the Acknowledgments Section of your manuscript:
"K.P. is a doctoral student at Universidad del Norte, Barranquilla, Colombia, and received a Ph.D. scholarship from this institution. Some of this work is to be presented to the Ph.D. program in partial fulfilment of the requirements for the Ph.D. degree. The sponsor of the study has no role in study design; in the collection, analysis, and interpretation of data; in the writing of the report; and in the decision to submit the article for publication."

We note that you have provided funding information that is not currently declared in your Funding Statement. However, funding information should not appear in the Acknowledgments section or other areas of your manuscript. We will only publish funding information present in the Funding Statement section of the online submission form.
Please remove any funding-related text from the manuscript and let us know how you would like to update your Funding Statement. Currently, your Funding Statement reads as follows:
"The author(s) received no specific funding for this work."

Please include your amended statements within your cover letter; we will change the online submission form on your behalf.

We appreciate your correction. As per your request, we have removed any funding-related text from the manuscript and clarified that Universidad del Norte provided only tuition expenses for our PhD student.

5. In your Data Availability statement, you have not specified where the minimal data set underlying the results described in your manuscript can be found. PLOS defines a study's minimal data set as the underlying data used to reach the conclusions drawn in the manuscript and any additional data required to replicate the reported study findings in their entirety. All PLOS journals require that the minimal data set be made fully available. For more information about our data policy, please see http://journals.plos.org/plosone/s/data-availability.

Upon re-submitting your revised manuscript, please upload your study's minimal underlying data set as either Supporting Information files or to a stable, public repository and include the relevant URLs, DOIs, or accession numbers within your revised cover letter. For a list of acceptable repositories, please see http://journals.plos.org/plosone/s/data-availability#loc-recommended-repositories. Any potentially identifying patient information must be fully anonymized.

Important: If there are ethical or legal restrictions to sharing your data publicly, please explain these restrictions in detail. Please see our guidelines for more information on what we consider unacceptable restrictions to publicly sharing data: http://journals.plos.org/plosone/s/data-availability#loc-unacceptable-data-access-restrictions. Note that it is not acceptable for the authors to be the sole named individuals responsible for ensuring data access.

We will update your Data Availability statement to reflect the information you provide in your cover letter.

Thank you!

6. We note that Figures 1, 5 9 and S2 in your submission contain map images which may be copyrighted. All PLOS content is published under the Creative Commons Attribution License (CC BY 4.0), which means that the manuscript, images, and Supporting Information files will be freely available online, and any third party is permitted to access, download, copy, distribute, and use these materials in any way, even commercially, with proper attribution. For these reasons, we cannot publish previously copyrighted maps or satellite images created using proprietary data, such as Google software (Google Maps, Street View, and Earth). For more information, see our copyright guidelines: http://journals.plos.org/plosone/s/licenses-and-copyright.

We require you to either (1) present written permission from the copyright holder to publish these figures specifically under the CC BY 4.0 license, or (2) remove the figures from your submission:

a. You may seek permission from the original copyright holder of Figures 1, 5, 9 and S2 to publish the content specifically under the CC BY 4.0 license.

We recommend that you contact the original copyright holder with the Content Permission Form

(http://journals.plos.org/plosone/s/file?id=7c09/content-permission-form.pdf) and the following text:
“I request permission for the open-access journal PLOS ONE to publish XXX under the Creative Commons Attribution License (CCAL) CC BY 4.0 (http://creativecommons.org/licenses/by/4.0/). Please be aware that this license allows unrestricted use and distribution, even commercially, by third parties. Please reply and provide explicit written permission to publish XXX under a CC BY license and complete the attached form.”
Please upload the completed Content Permission Form or other proof of granted permissions as an ""Other"" file with your submission.

In the figure caption of the copyrighted figure, please include the following text: “Reprinted from [ref] under a CC BY license, with permission from [name of publisher], original copyright [original copyright year].”

We are very appreciative of your comments.

Figures containing maps were created using the plot function from GeoPandas (https://geopandas.org/en/stable/), which is an open-source library in Python. This library has a permissive license similar to the BSD 2-Clause License, and it has permissions for distribution, private use, modification, and commercial use (see https://github.com/geopandas/geopandas/blob/main/LICENSE.txt).

On the other hand, when creating all maps, we used a shape file that contains the location’s geometry. This shapefile was retrieved from https://hub.arcgis.com/datasets/de0e829ddbf743c895ba6dcee1b74fae/about. According to the author, this file can be freely accessible and used, which indicates that neither a license nor permission is needed to use it (i.e., https://hub.arcgis.com/datasets/de0e829ddbf743c895ba6dcee1b74fae/about). The following image, in Spanish, was obtained from the author’s profile and reaffirms this:

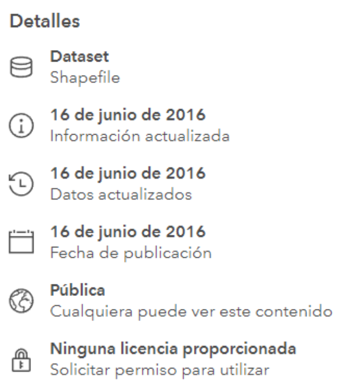

b. If you are unable to obtain permission from the original copyright holder to publish these figures under the CC BY 4.0 license or if the copyright holder’s requirements are incompatible with the CC BY 4.0 license, please either i) remove the figure or ii) supply a replacement figure that complies with the CC BY 4.0 license. Please check copyright information on all replacement figures and update the figure caption with source information. If applicable, please specify in the figure caption text when a figure is similar but not identical to the original image and is therefore for illustrative purposes only.
The following resources for replacing copyrighted map figures may be helpful:

USGS National Map Viewer (public domain): http://viewer.nationalmap.gov/viewer/
The Gateway to Astronaut Photography of Earth (public domain): http://eol.jsc.nasa.gov/sseop/clickmap/
Maps at the CIA (public domain): https://www.cia.gov/library/publications/the-world-factbook/index.html and https://www.cia.gov/library/publications/cia-maps-publications/index.html
NASA Earth Observatory (public domain): http://earthobservatory.nasa.gov/
Landsat: http://landsat.visibleearth.nasa.gov/
USGS EROS (Earth Resources Observatory and Science (EROS) Center) (public domain): http://eros.usgs.gov/#
Natural Earth (public domain): http://www.naturalearthdata.com/

Kindly see our response to Comment #6. As mentioned above, a license to use the maps is not required.

7. Please include captions for your Supporting Information files at the end of your manuscript, and update any in-text citations to match accordingly. Please see our Supporting Information guidelines for more information: http://journals.plos.org/plosone/s/supporting-information.

We very much appreciate your supporting comments. We have updated our manuscript according to the journal's formatting.

- We added the Supporting Information captions in the manuscript after references.
- We separated the supplementary materials into four (4) files: S1_Table, S2_Table2, S3_Fig, and S4_Fig.
- We changed the Supporting Information citation on the manuscript i.e. "Table 1S, supplementary material" to "S1 Table".

.

Comments from Reviewer #1

1. The manuscript was well constructed. However, the methodology seems very technical, with no explanation of what data were used, how and where the analyzes were carried out. I suggest making the methodology clearer, for example in CAE not only citing authors who used the technique but making it clear why it was chosen and not another deep learning technique.

We very much appreciate the reviewer's comments.

We used data from the Colombian National Survey on Psychoactive Substance Consumption. Data were extracted from https://microdatos.dane.gov.co/index.php/catalog/680/data-dictionary. Analyses were performed using Python's libraries GeoPandas (https://geopandas.org/en/stable/), TensorFlow (https://www.tensorflow.org/), PyTorch (https://pytorch.org/) and scikit-learn (https://scikit-learn.org/stable/), among others. All notebooks and code written for processing and analyzing the data are available from first author under reasonable request.

Following the reviewer's suggestion, we have modified the methodology in the revised version of the manuscript. We added a subtitle in the methodology denominated "Framework evaluation."

Now, the text reads:

"2.8 Framework Evaluation.

We trained the CAE-DEC method using data retrieved from the National Survey of Psychoactive Substance Consumption (DANE-DIMPE-ENCSPA-2019), which contains 49,600 observations. A second database with PAS production figures, was used in the spatial analysis stage to correlate the PSA consumption and production. In order to evaluate the framework, we compared our CAE-DEC approach with other approaches, including CAE, and Principal Component Analysis integrated with clustering (PCA-DEC). For evaluation and comparison purposes, we use the Calinski-Harabasz [76], Davies-Bouldin [77], and Silhouette [78] index as intrinsic clustering metrics. In addition, we used the χ^2 statistic to investigate potential associations and differences among the patterns (clusters) identified using our approach."

On the other hand, we rewrote a paragraph in the methodology to make clear why the model was chosen. Now, the text reads:

"Fig 1 presents the proposed Convolutional Auto-Encoder- Deep Embedded Clustering (CAE-DEC) framework based on the implementation presented by Xie et al. [57]. However, unlike the Xie et al. model, our structure is developed by applying convolutional layers for the deep autoencoder (DA) architecture instead of a linear one to represent high-order interactions in the data. In addition, a spectral clustering-based centroid estimation is proposed to achieve an improved initial centroid calculation. We chose the CAE-DEC framework based on its ability to reduce both the number of model parameters and the dimensionality, while creating clusters simultaneously."

"

2. In the results, improve the identification of tables and figures. Include subtitles to make files more understandable.

Thank you for your comment. As suggested and following the Journal's formatting for Figures and Table file naming, we have improved the tables and figures captions in the revised version of the manuscript (i.e., from "Palomino-fig-1" to "Fig1").

3. In the Discussion, explore further the impact of the results and assess the environmental issue of these sites. There was a stratification of regions and consumption profile, but there is no information about the location. The area is urban or rural, what level of development. Perhaps these and other local (environmental) aspects may be related and/or favour the consumption of these substances.

Thank you for your suggestion. We improved the discussion section accordingly in the revised version of the manuscript. The relevant text now reads:

"In this study, we propose and test a Deep Neural Network-based Clustering-oriented Embedding algorithm (i.e., a ML-based model) for identifying psychoactive substance (PAS) use and abuse patterns in Colombia. This model allows the automatic extraction of features from the input data (such as sex, age, socioeconomic status, and housing type) to determine whether an individual has consumed PAS. It then creates clusters in the new data space generated during the learning process, following the methods outlined in [57, 59]. After the training process, a latent feature space (LFS) is generated and the results are subsequently analysed. We have identified clearly marked clusters where the prevalence of individuals who use or do not use PAS is notable. Additionally, we found that region, sex, housing type, socioeconomic strata, age, and whether individuals contribute to household finances have a statistically significant impact on the clustering structure. These findings are consistent with previous studies aimed at identifying PAS consumption patterns [19, 80, 81]. Interestingly, when comparing the CAE-DEC model proposed in this study and the CAE-Spectral model using different metrics (i.e., Silhouette statistic, which measures the internal density of each cluster and the distance that separates them from each other, the Calinski-Harabasz index and the Davies-Bouldin index [DBI]), we found that our model performs better (Silhouette: 0.62 vs. 0.786; Calinski-Harabasz: 22468.26 vs. 775992.45; DBI: 0.2898 vs. 0.63; ***S2 Table*****,** ***Supplementary Material****).*

*Based on our findings, individuals more likely to consume PAS are grouped in cluster 2, while cluster 1 consisted of individuals who did not consume PAS (****Table 1****). Not surprisingly, a significant proportion of females characterizes cluster 1. In addition, most individuals belong to socioeconomic strata 1, are 40 years old or older, and do not contribute economically to support their household. In contrast, cluster 2 is characterized by a higher proportion of males aged between 20 and 40 in socioeconomical strata 1 and 2, who do not contribute to the household finances (****Table 1****). Finally, cluster 0 is characterized by a small proportion of males, a higher proportion of individuals in strata 3, 4, 5, and 6, and individuals are more likely to contribute to the household economy (****Table 1****).*

*At the level of spatial statistics, we identified that legal drugs such as alcohol have a high prevalence in all regions of Colombia, with a slight tendency to more consumption in coastal areas (****Fig 7****). In our country, the coastal areas are often popular tourist destinations, and many tourists come to these areas looking for a relaxing experience, which can increase alcohol consumption. Coastal areas typically have warmer*

*temperatures and more sunshine, increasing thirst and making people more likely to consume alcohol. Additionally, bars, clubs, and restaurants serve alcoholic beverage due to the high demand from tourists and locals [82, 83]. Another characteristic of this area is the fishing and maritime culture. This culture is often associated with hard work and long working hours, and alcohol may be seen as a way to relax and unwind after a tough day at the sea [84]. Finally, this region has 68% urban and 32% rural zones [60]. The level of development, as measured by gross domestic product (GDP), is the third region with significant economic development in the country [85] (**S3 Table**, **Supplementary Material**). Interestingly, the consumption of illegal drugs is lower in the Northern region than in other regions of the country. However, there is a more representative consumption of non-prescription tranquilizers, opioids, ketamine, GHB, and heroin. In particular, the Atlántico department has the highest consumption proportion within this region (**Fig 8**).*

*Tobacco consumption is present in all regions, with a higher proportion in the Central region, where climate conditions resemble temperate weather. Also, this region has a diverse consumption pattern, where drugs such as marijuana, popper, cocaine, ecstasy, inhalants, methadone, heroin, LSD, GHB, 2CB, and mushrooms prevail. This region has Colombia's largest cities (i.e., Bogotá and Medellin); Bogotá has the highest population density and is a hub for drug trafficking routes, while Medellín has an unfortunate history of drug cartels and gang violence. Ultimately, this region is comprised of 77% urban areas, and the most developed cities in the country are located there [60, 85] (**S3 Table**, **Supplementary Material**). Energy drinks are more frequently used in the Eastern region, characterized by a continental climate surrounded by flat territory. Our results are in line with the scientific literature suggesting that the location of regions within countries is directly associated with the consumption of PAS [26, [86–88]. The consumption of heroin, basuco, non-prescription tranquilizers, stimulants, methamphetamines, opioids, and ketamine characterizes this region. This zone is the second most developed region in the country, and 72% of urban areas [60], [85](**S3 Table**, **Supplementary Material**).Our findings also show that the Southern region is more likely to consume illegal drugs, including basuco, heroin, and Yagé (**Fig 8**). One of the main reasons for this result is that, unfortunately, this region has favourable environmental characteristics (i.e., majority rainforest) for their consumption and production, being the second largest illegal drug-producing region in Colombia [79]. Furthermore, this region has the highest percentage of rurality (52%) compared to the other regions, and its level of development is low as measured by the GDP [60, 85] (**S3 Table**, **Supplementary Material**).*

*In the Western region, also known as the Pacific region, consumption mostly mainly includes of Methylene Chloride (DICK), GHB, heroin, opioids, and methamphetamines. This region (Pacific) is mainly characterized known for its geographical isolation, poverty, and ongoing conflict, which have contributed to the growth of drug production and trafficking in the area. Poverty is one of the main factors driving drug production in the Pacific region, which has led many people to turn to drug cultivation and trafficking for survival. Additionally, the region's rugged terrain and limited infrastructure have made it difficult for the Colombian government to establish a strong presence, allowing drug traffickers to operate with relative impunity [89]. This region has a similar percentage of urban (53%) and rural (47%) populations than the Southern region, and ranks second among the regions with the lowest levels of development (**S3 Table**, **Supplementary Material**). In the Western region, also known as the Pacific region, consumption mainly includes methylene chloride, GHB, heroin, opioids, and methamphetamines. This region is mainly characterized for its geographical isolation, poverty, and ongoing conflict, which have contributed to the growth of drug production and trafficking in the area. Poverty is one of the main factors driving drug*

.

*production in the Pacific region, which has led many people to turn to drug cultivation and trafficking for survival. Additionally, the region's rugged terrain and limited infrastructure have made it difficult for the Colombian government to establish a strong presence, allowing drug traffickers to operate with relative impunity [89]. This region has a similar percentage of urban (53%) and rural (47%) populations than the Southern region, and ranks second among the regions with the lowest levels of development (**S3 Table**, **Supplementary Material**).[89]. This region has a similar percentage of urban (53%) and rural (47%) populations than the Southern region. On the other hand, this region ranks second among the regions with the lowest levels of development (**S3 Table**, **Supplementary Material**)."*

.

Comments from Reviewer #2

1. Abstract should rewrite and included technical approach more.

Done.

2. page 5 "Feature selection approaches for clustering can be split into filter, wrapper, embedded," are these methods just for clustering!!?

Thank you for your comment.

No, feature selection approaches do not only apply for clustering algorithms.

Feature selection is the process of selecting a subset of relevant features (variables and/or attributes) to be used in a supervised or unsupervised model and constitutes an important step in Machine Learning as including irrelevant or redundant features in a model can lead to overfitting, decreased model performance, and increased computational complexity. In this sense, feature selection approaches can be useful for classification, clustering, or regression.

3. In the Introduction, please give problem, challenges clearly. Introduction is written separately.

Thank you for your input. Following your advice, several changes have been made in the Introduction of the revised version of the manuscript to address this.

4. In introduction did not cite many reverences together" IDEC (Guo et al., 2017), DEPICT (Dizaji et al., 2017), DBC (Li et al., 2017), DualAAE (Ge et al., 2020), VAED (Lim et al., 2020), and DNC (B. Li et al., 2021) "

Done.

5. What is difference between convolutional auto-encoder (CAE) and stacked space auto encoder?

Both Convolutional Autoencoder (CAE) and Stacked Autoencoder (SAE) are types of autoencoders, a type of neural network architecture that is used for unsupervised learning and data compression.

The main difference between CAE and SAE is the way they handle the input data. A CAE is typically used for processing image data. It uses convolutional layers to extract spatial features from the input image and then uses deconvolutional layers to reconstruct the image. CAEs are well-suited for image data because they can capture the spatial relationships between pixels in an image and can learn to recognize visual patterns and shapes. On the other hand, SAE is typically used for processing structured or unstructured data. It consists of multiple layers of neural networks that encode the input data into a lower-dimensional

representation and then decode it back to the original dimensions. SAEs are useful for feature learning and data compression in many different types of data, including text, audio, and structured data.

In summary, while CAE is focused on processing image data using convolutional layers, SAE can be applied to various types of data and uses multiple layers of neural networks for encoding and decoding.

6. Did not compare other previous works.

Thank you for the comments. In the supplementary section, **S2 Table** shows the comparison of our model with PCA-*K*-means, and CAE-Spectral.

Table S2. Performance metrics for different models.

Performance metric	CAE-DEC	PCA-*K*-means	CAE-Spectral
Calinski-Harabasz	775992.45	128651.83	22468.26
Davies-Bouldin	0.2898	0.567	0.63
Silhouette	0.786	0.6061	0.62

In this sense, **S2 Table** shows that our CAE-DEC model results in the highest Calinski-Harabasz score, which means that identified clusters are dense and well separated. On the other hand, our model has the smallest Davies-Bouldin score, which indicates that identified clusters groups have a better partition. Regarding the Silhouette index, our model gives the highest value, which implies that clusters are highly dense.

Performance metric interpretation

- Calinski-Harabasz score: A high score indicates that clusters are dense and well separated.
- Davies-Bouldin score: Lower is better. Lower values indicating better clustering.
- Silhouette index: The best value is 1 and the worst value is -1. Values near 0 indicate overlapping clusters. Negative values generally indicate that a sample has been assigned to the wrong cluster, as a different cluster is more similar.

7. Your problem is classification or clustering?

We appreciate your question.

The problem we are addressing is this study is a clustering problem, as we aim to identify consumption patterns of psychoactive substances (PAS) in the Colombian territory. In particular, we used clustering techniques (i.e., an ensemble model that integrates an autoencoder and cluster method) to find the different patterns of PAS consumptions in Colombian citizens considering the consumers' location.

.

8. Please give your performance metrics

The performance metrics are stated in the Discussion of the manuscript. The relevant text reads:

"Interestingly, when comparing the CAE-DEC model proposed herein and the CAE-Spectral model using different score metrics (i.e., Silhouette score, which measures the internal density of each cluster and the distance that separates them from each other, the Calinski-Harabasz index and the Davies-Bouldin index [DBI]) showed that the our proposal model performs better (Silhouette: 0.62 vs. 0.786; Calinski-Harabasz: 22468.26 vs. 775992.45; DBI: 0.2898 vs. 0.63) than the CAE-Spectral alone model"

More information about performance metrics can be found in **S2 Table** of the **Supplementary Material**. According to our results, the proposed CAE-DEC model shows a well-separated and highly dense cluster, meaning we can define better groups and identify PAS consumer patterns more precisely.
