# Supplementary material for: Leading consumption patterns of psychoactive substances in Colombia: A deep neural network-based clustering-oriented embedding approach
Source: PLoS One. 2023 Aug 18;18(8):e0290098. doi: 10.1371/journal.pone.0290098 (PMC10438020; doi:10.1371/journal.pone.0290098)
Supplement: Attachment — Submitted filename: Response to Reviewers.docx [file pone.0290098.s010.docx]

Barranquilla, July 20, 2023

Dr. Vinicius Silva Belo

Academic Editor, *PLOS ONE*

RE: Revised Manuscript # PONE-D-22-28262

Dear Dr. Silva,

Thank you for the opportunity to submit a revised version of our manuscript, "***Leading Consumption Patterns of Psychoactive Substances in Colombia: A Deep Neural Network-based Clustering-oriented Embedding Approach***,” which now includes responses to the concerns, inquiries, comments, and suggestions raised by two anonymous reviewers. Please find below our response (in **blue**).

We very much appreciate all your efforts as Editor-in-Chief and the detailed and extraordinary revision this manuscript had. We enjoyed the thorough review of our manuscript, and it was a pleasure to respond to the reviewers. Please, Dr. Silva, allows us to mention that this exercise of the fair and exigent peer-review process is disappearing, and only good journals like *PLOS ONE* keep it. We hope that our new version might be suitable for publication in *PLOS ONE.*

Thank you very much for your time and consideration.

Yours sincerely,

______________________________

Kevin R. Palomino, PhD(c)

Corresponding author

**Comments from the Editor**

1. I suggest including a location map in the methodology section. The map will offer a clear spatial reference, enabling readers to visualize precise locations of study points or mentioned areas. The inclusion of a location map can improve overall clarity and comprehension, making the article more accessible to a broad audience, including non-specialist readers.

We very much appreciate your comments. We have added a locations map in the methodology section. The relevant text now reads:

*“Located in South America, the Republic of Colombia is a diverse country with a population of over 50 million people distributed over a territory of 440,831 square miles [60], encompassing jungles, highlands, grasslands, deserts, coasts, and islands, distributed in six regions and 32 departments (states)[61], (see S1 Fig). It is worth noting that, unfortunately, Colombia has been a major producer of illegal drugs for a long time, which has had a significant impact on drug consumption and abuse.”*

2. On page 25, Line 7, I suggest improving the following sentence: *“Coastal areas are known to have warmer temperatures and higher levels of sunshine, which can contribute to increased thirst and a higher likelihood of alcohol consumption among people.”* The increase in temperature contributes to increased beverage consumption, but not necessarily alcoholic beverages.

Thank you for your suggestion. We improved the discussion section accordingly in the revised version of the manuscript. The relevant text now reads:

In our country, the coastal areas are often popular tourist destinations, and many tourists come to these areas looking for a relaxing experience, which can increase alcohol consumption. Coastal areas typically have warmer temperatures and more sunshine, increasing thirst and making people more likely to consume beverage. Additionally, bars, clubs, and restaurants serve alcoholic beverage due to the high demand from tourists and locals.
